# Supplementary material for: An organic proton cage that is ultra-resistant to hydroxide-promoted degradation
Source: Nat Commun. 2024 Apr 22;15:3395. doi: 10.1038/s41467-024-47809-0 (PMC11035699; doi:10.1038/s41467-024-47809-0)
Supplement: Supplementary file 1 — Supplementary Information [file 41467_2024_47809_MOESM1_ESM.pdf]

Supplementary Materials for

An Organic Proton Cage that is Ultra-Resistant to Hydroxide-Promoted Degradation

Chase L. Radford, Torben Saatkamp, Andrew J. Bennet*, Steven Holdcroft*
Corresponding author: holdcrof@sfu.ca
bennet@sfu.ca

The PDF file includes:

Supplementary Text
Supplementary Figs 1 to 78
Supplementary Tabs 1 to 2
Characterization Data
References

Contents

Contents 2
Supplementary methods: 3
Synthesis 3
1,6-diazabicyclo[4.4.0]dec-3,8-diene-2,5-dione:[2] 4
1,6-diazabicyclo[4.4.0]decan-2,5-dione:[2] 5
1,6-diazabicyclo[4.4.0]decane:[3] 6
1,6-diaza-1-(4-bromobutyl)bicyclo[4.4.0]decan-1-ium bromide:[4] 7
1,6-diazatricyclo[4.4.4.0^{1,6}]tetradecan-1,6-diylium tetrafluoroborate:[4] 8
1,6-diazabicyclo[4.4.4]tetradecan-1-ium [outside proton] tetrafluoroborate:[4] 9
1,6-diazabicyclo[4.4.4]tetradecan-1,6-ium [inside proton] tetrafluoroborate:[1] 10
1,6-diazabicyclo[4.4.4]tetradecan-1,6-ium [inside proton] chloride: 13
6-azaspiro[5.5]tetradec-6-ium bromide:[5] 16
N,N’-dimethyl piperidinium iodide:[5] 17
2-mesityl-4,5-diphenyl-1H-imidazole:[6] 18
1,3-dimethyl-2-mesityl-4,5-diphenyl-1H-imidazol-3-ium iodide:[7] 18
NMR degradation study: 19
DVS degradation studies: 44
DFT calculations: 64
Supplementary Discussion 65
Supplementary References 65

Supplementary methods:

<u>Synthesis</u>

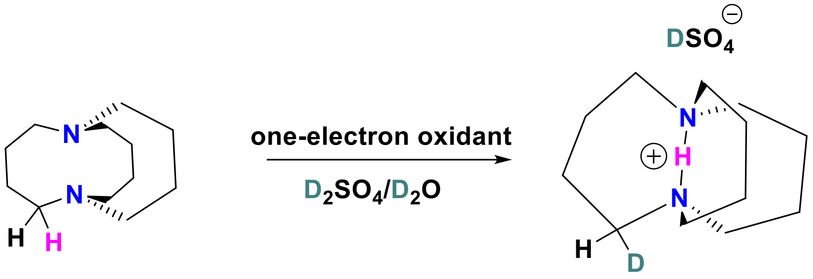

Supplementary Fig 1. Proton insertion mechanism as reported by Alder et al.[1]

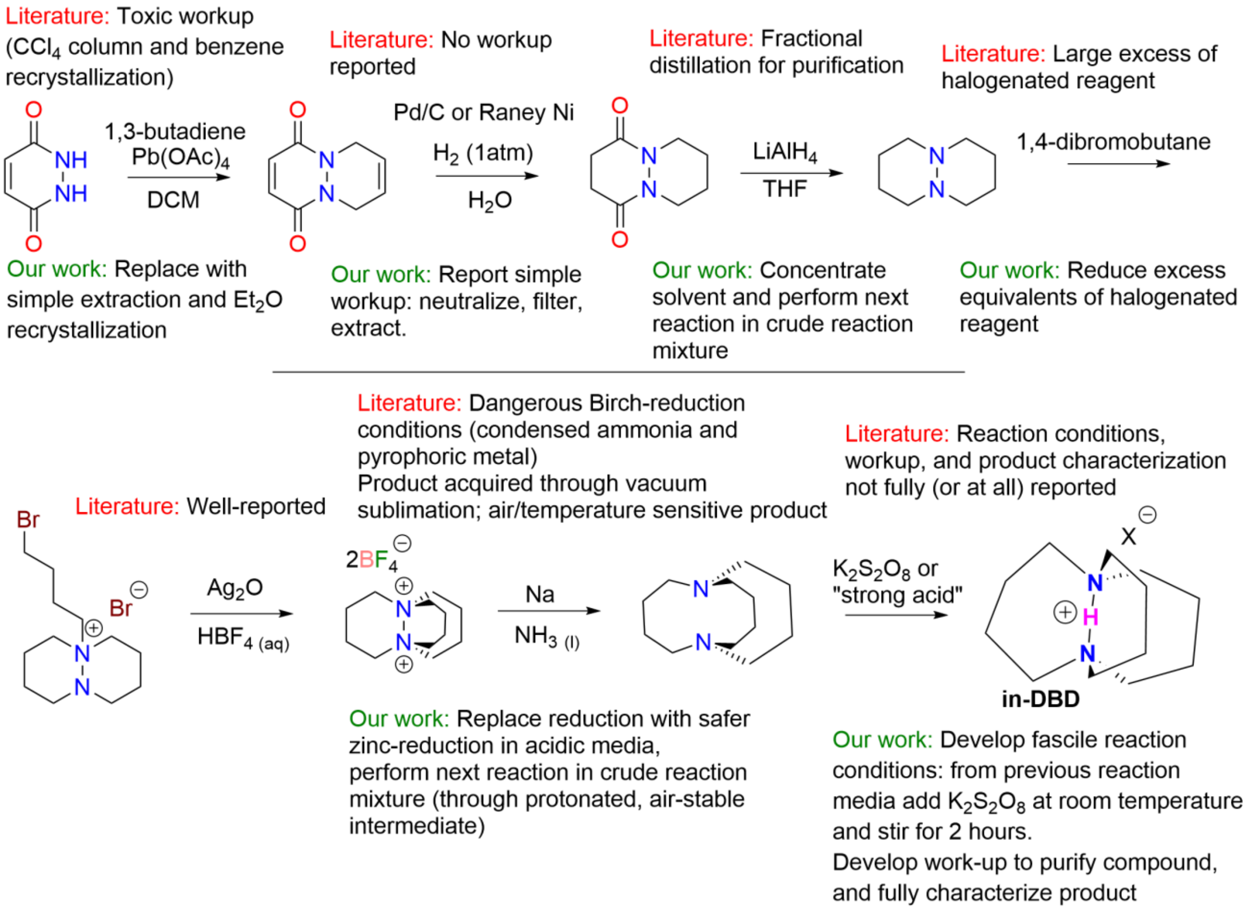

Supplementary Fig 2. Literature-reported synthesis of in-DBD (from multiple reports, cited below) with the reported reaction conditions. Reported issues with the literature synthesis are given, and our work to remove these constraints to safety, scalability, and lack of reporting.

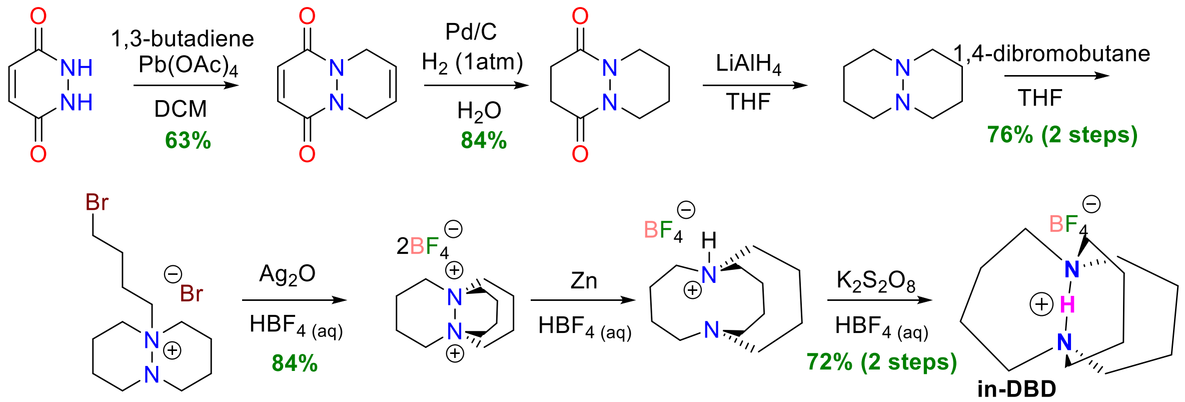

1,6-diazabicyclo[4.4.4]tetradecan-1,6-ium tetrafluoroborate

Supplementary Fig 3. Our synthetic pathway to 1,6-diazabicyclo[4.4.4]tetradecan-1,6-ium [inside proton] tetrafluoroborate.

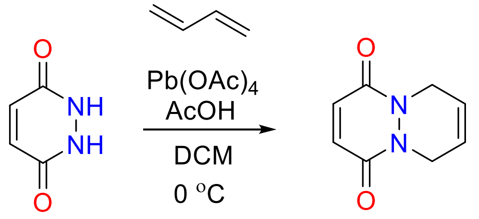

<u>1,6-diazabicyclo[4.4.0]dec-3,8-diene-2,5-dione:[2]</u>

In a round-bottom flask, DCM (250 mL), and acetic acid (5 mL) were cooled to 0 °C, and added maleic hydrazide (5.75 g, 51.3 mmol) and a solution of 20 wt% 1,3-butadiene in toluene (34.40 mL, 102.5 mmol). In roughly 25 portions, lead tetraacetate (25.00 g, 56.38 mmol) was added over the course of roughly 6 hours at 0 °C, allowing each portion to fully react*. The mixture was allowed to warm to room temperature overnight, then quenched with water. The organic layer was extracted, washing with water, and dried with MgSO4. The solvent was removed under reduced pressure, then the product was triturated in diethyl ether (~ 100 mL) and cooled to -20 °C overnight. The product was filtered, collecting product as a yellow powder (5.33 g, 32.5 mmol, 63% yield). 1H NMR (400 MHz, $CDCl_3$): δ 6.92 (s, 2H), 6.03 (t, *J* = 1.4 Hz, 2H), 4.49 (d, *J* = 1.1 Hz, 4H).

* to test for consumption of $Pb(OAc)_4$, a glass pipette tip was dipped into the solution and the sample was dropped onto a wetted paper towel. If a brown drop was observed, the reaction was allowed to continue, if the drop remained a pale yellow, more $Pb(OAc)_4$ was added to the reaction.

SAFETY NOTE: lead tetraacetate is highly toxic and should be handled with care to avoid contact with skin or through inhalation. After hydrolysis the lead oxide still presents a significant danger to inhalation and to aquatic releases. Care when handling and disposing should be taken.

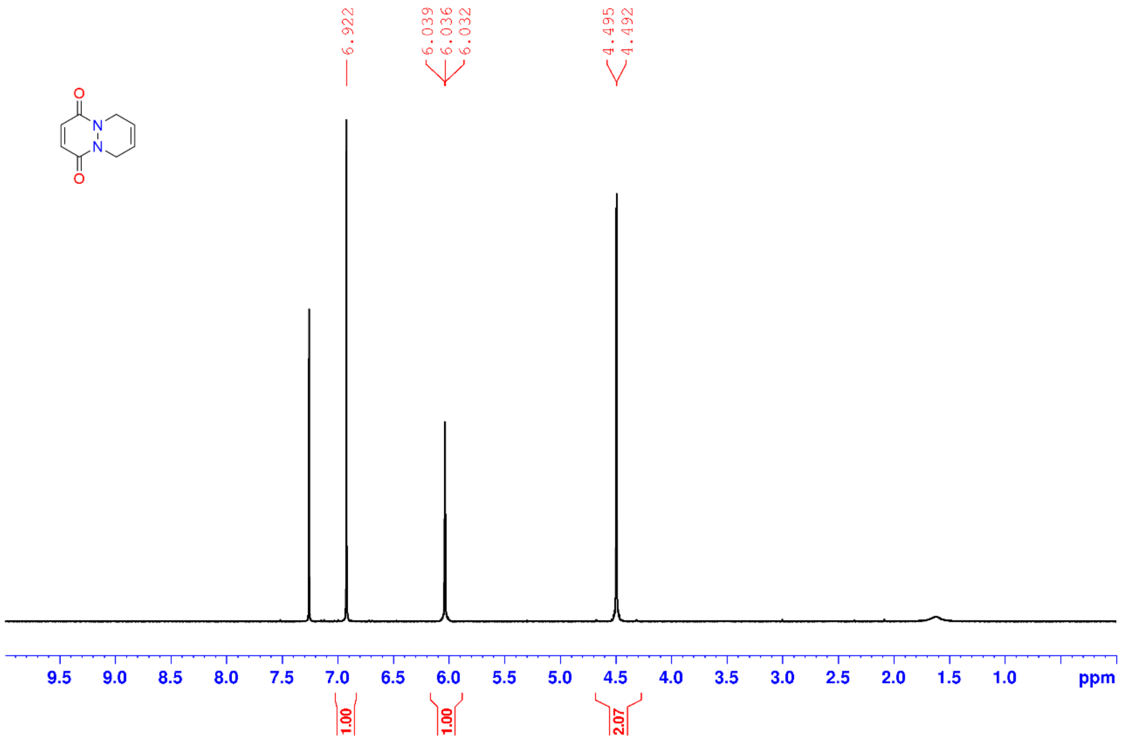

Supplementary Fig 4. ^{1}H NMR spectrum (400 MHz) of 1,6-diazabicyclo[4.4.0]dec-3,8-diene-2,5-dione in $CDCl_3$.

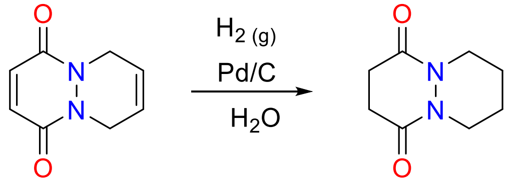

1,6-diazabicyclo[4.4.0]decan-2,5-dione:[2]

In a round-bottom flask, 5% Pd/C (1.70 g) was suspended in deionized water (150 mL). 6,9-1,6-diazabicyclo[4.4.0]dec-3,8-diene-2,5-dione (4.30 g, 26.2 mmol) was added and the headspace of the flask was evacuated using a water aspirator, re-filling the atmosphere with a balloon of $H_{2(g)}$ three times. The mixture was then stirred at room temperature under a H_2 atmosphere for 2 days, re-filling the hydrogen balloon when nearly consumed. When complete, the hydrogen was removed slowly under a stream of argon, then celite (15 g) was added and stirred for ~ 10 minutes. The mix was filtered, washing with 0.5 M K_2CO_3, then water, then ethyl acetate, then DCM. The rinsings were all collected and combined, and the product was extracted with DCM ×3, the organic layer was dried with $MgSO_4$ and solvent was removed to obtain the product as an off-white powder (3.68 g, 21.9 mmol, 84% yield). 1H NMR (400 MHz, $CDCl_3$): δ 3.74 (m, 4H), 2.60 (s, 4H), 1.71 (p, J = 2.90 Hz, 4H).

SAFETY NOTE: Hydrogen gas presents an explosion risk, especially when mixed with oxygen. Palladium on carbon can cause ignition of organic solvents upon contact, especially methanol, and should be handled with care.

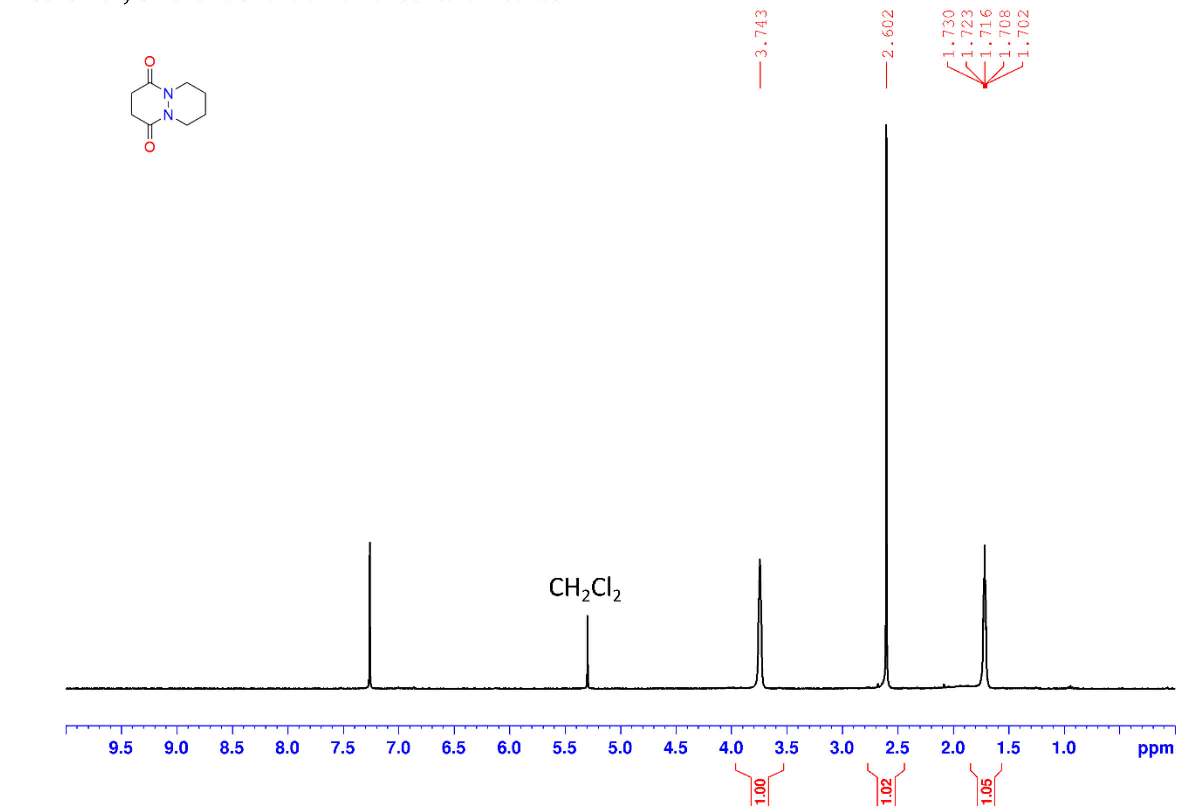

Supplementary Fig 5. ^{1}H NMR spectrum (400 MHz) of 1,6-diazabicyclo[4.4.0]decan-2,5-dione in $CDCl_3$. Residual dichloromethane solvent signal marked by CH_2Cl_2.

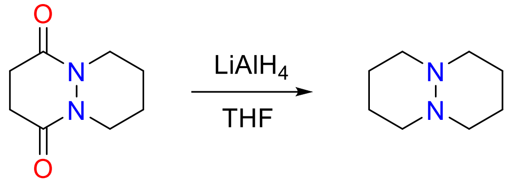

1,6-diazabicyclo[4.4.0]decane:[3]

In a flame-dried 2-neck flask affixed with a reflux condenser, dry THF (60 mL) and 1,6-diazabicyclo[4.4.0]decan-2,5-dione (3.50 g, 20.8 mmol) were mixed under argon atmosphere, sparging with argon for ~ 30 minutes. To the mixture, solid $LiAlH_4$ (3.16 g, 83.2 mmol) was added slowly in portions, maintaining a soft reflux. The mixture was then refluxed under an argon atmosphere for 3 days. The mixture was then cooled, quenched dropwise with water (6.00 mL, 333 mmol), then filtered the solid. The solid was collected and triturated 2 times in additional THF (20 mL), recombining all organic fractions and concentrating on rotary evaporator at 330 mbar in 40 °C water to ~ 25 mL volume. This solution was used without further purification for the next reaction.

SAFETY NOTE: Solid $LiAlH_4$ is a pyrophoric solid that produces hydrogen gas upon ignition. It should be handled under argon at all times.

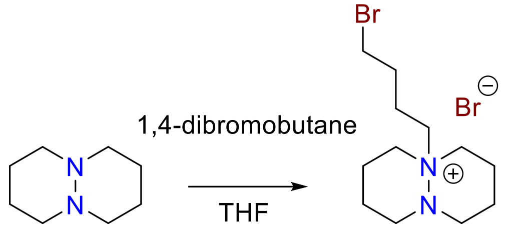

1,6-diaza-1-(4-bromobutyl)bicyclo[4.4.0]decan-1-ium bromide:[4]

In a 150 mL thick-walled glass pressure vessel, 1,6-diazabicyclo[4.4.0]decane from the previous reaction (nominally 20.8 mmol) in THF (25 mL) was sparged with argon for ~ 10 minutes. 1,4-dibromobutane (14.91 mL, 124.9 mmol) was added, and the flask was sealed under argon, heating the vessel to 90 °C overnight. The reaction was cooled, then carefully opened the flask and precipitated with diethyl ether (~50 mL). The mixture was allowed to cool to -20 °C overnight, then filtered, rinsing with cold diethyl ether to obtain the product as a tan powder (5.66 g, 15.9 mmol, 76% yield). 1H NMR (400 MHz, D_2O): δ 3.73 (d, J=13.00 Hz, 2H), 3.66 (t, J=8.54 Hz, 2H), 3.55 (t, J=6.32 Hz, 2H), 3.32 (m, J=4.74 Hz, 2H), 3.11 (m, J=4.92 Hz, 2H), 2.90 (d, J=13.47 Hz, 2H), 1.919-2.037 (m, 4H), 1.764-1.895 (m, 8H).

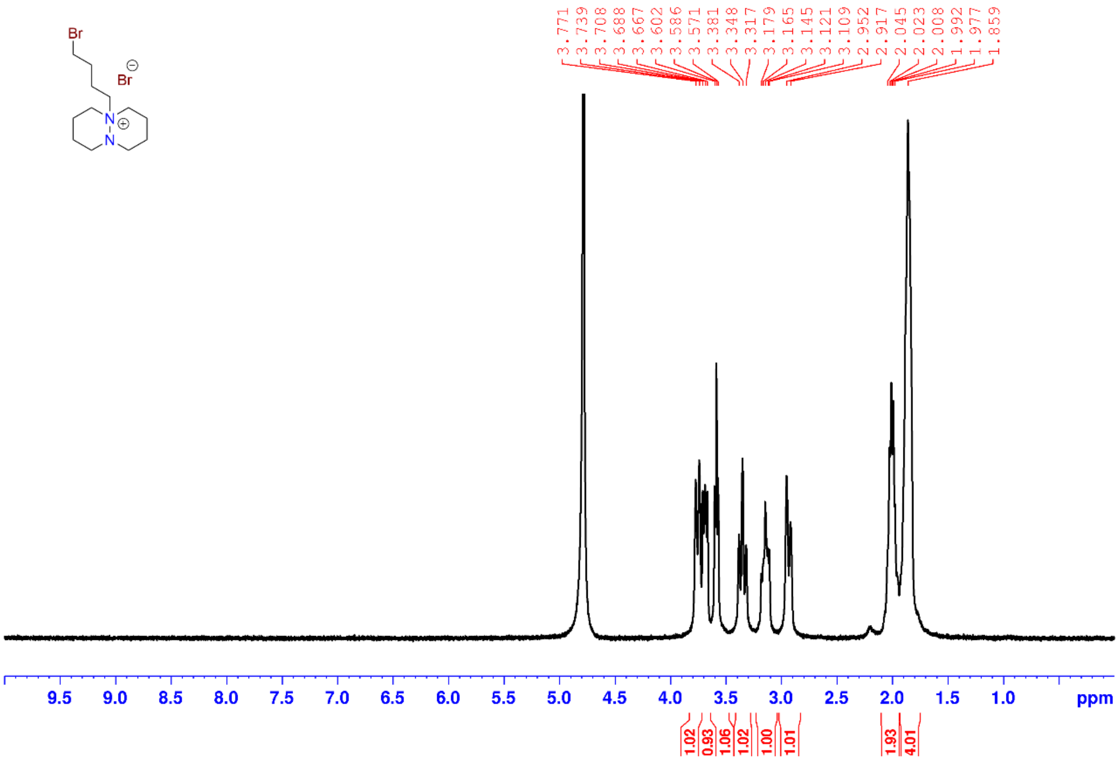

Supplementary Fig 6. ^{1}H NMR spectrum (400 MHz) of 1,6-diaza-1-(4-bromobutyl)bicyclo[4.4.0]decan-1-ium bromide in D_2O

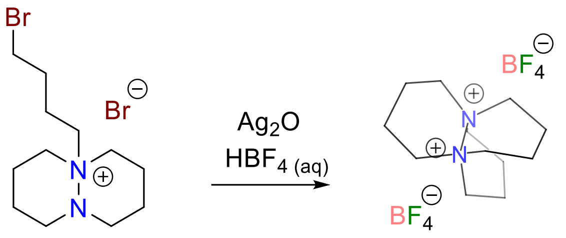

1,6-diazatricyclo[4.4.4.0^{1,6}]tetradecan-1,6-diylium tetrafluoroborate:[4]

In a round-bottom flask, Ag_2O (3.72 g, 16.1 mmol) was dissolved in 48% $HBF_{4\ (aq)}$ (25 mL). 1,6-diaza-1-(4-bromobutyl)bicyclo[4.4.0]decan-1-ium bromide (5.20 g, 14.6 mmol) was added to the mixture in portions. The mixture was then heated in a pre-heated 100 °C oil bath for 15 minutes, then filtered through a glass fritted filter, rinsing with 48% HBF_4 (10 mL). Ethanol (200 mL) was slowly added to the filtrate, and the mixture was cooled to -20 °C overnight to precipitate. The product was filtered, washing with cold ethanol, to obtain the product as a pale brown powder (4.56 g, 12.3 mmol, 84% yield). 1H NMR (400 MHz, D_2O): δ 5.03 (t, J=13.32 Hz, 6H), 3.82 (d, J=14.23 Hz, 6H), 2.49 (t, J=11.41 Hz, 6H), 2.20 (d, J=10.51 Hz, 6H). HRMS (m/z) [M]2+ calcd. for C12H24N2: 98.0964, found: 98.0965.

SAFETY NOTE: HBF_4 can contain small amounts of HF, and as such should be handled in a similar fashion.

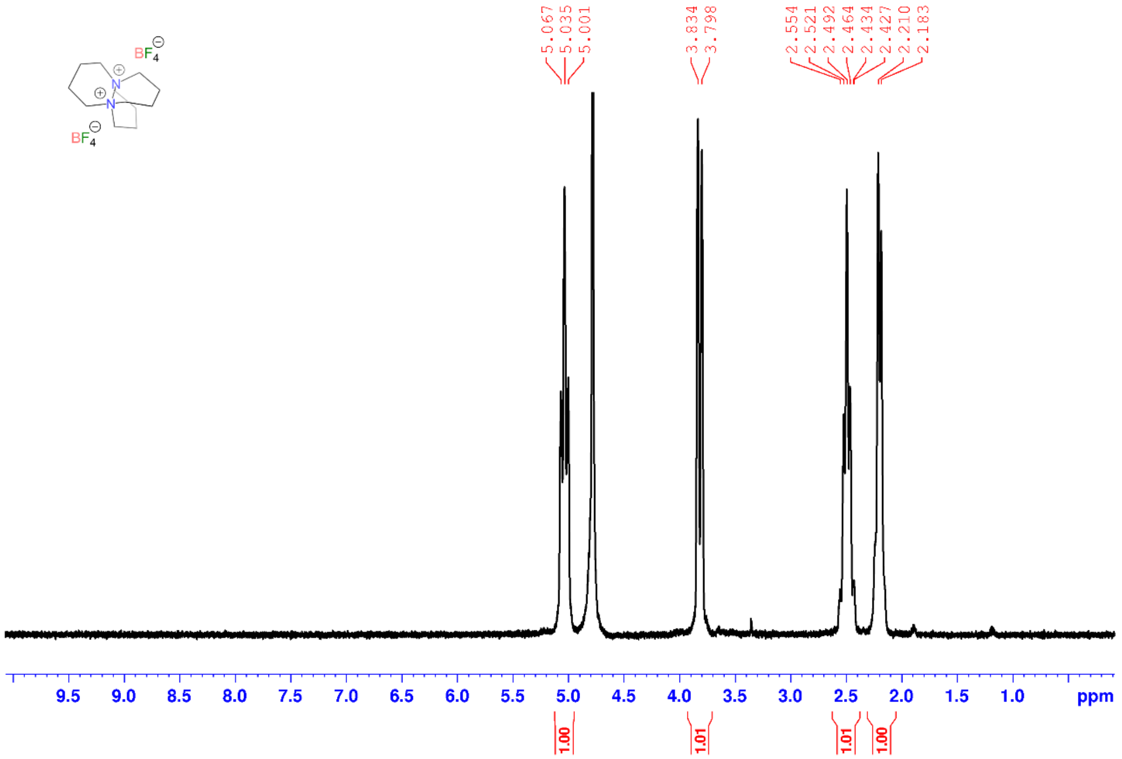

Supplementary Fig 7. ^{1}H NMR spectrum (400 MHz) of 1,6-diazatricyclo[4.4.4.0^{1,6}]tetradecan-1,6-diylium tetrafluoroborate in D_2O.

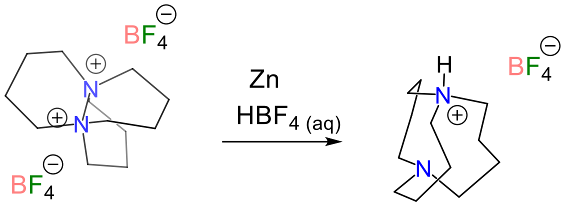

<u>1,6-diazabicyclo[4.4.4]tetradecan-1-ium [outside proton] tetrafluoroborate:</u>[4]

In a Schlenk flask, 48% HBF_4 (10 mL) was sparged with argon for ~ 10 minutes, then added 1,6-diazoniatricyclo[4.4.4.$0^{1,6}$]tetradecane tetrafluoroborate (2.00 g, 5.41 mmol), stirring until dissolved. Zinc shavings (1.77 g, 27.0 mmol) were added, and the mixture was stirred vigorously for 1 hour, under argon. The mixture was filtered, rinsing with minimal 48% HBF_4 (5 mL) and used for the next reaction.

Alternatively to isolate the product, to the mixture was slowly added degassed 5 M KOH under argon* until basic, then extracted with degassed diethyl ether ×3, adding the extracted organic layer quickly and directly into a solution of 1:8 48% HBF_4 to diethyl ether. The precipitate was filtered, washing with diethyl ether to obtain the product as a pale pink powder (81% isolated yield). 1H NMR (400 MHz, D_2O): δ 3.40 (t, J=4.97 Hz, 6H), 2.57 (t, J=5.32 Hz, 6H), 1.97 (p, J=5.54 Hz, 6H), 1.72 (p, J=5.95 Hz, 6H). HRMS (m/z) [M]+ calcd. for C12H25N2: 197.2012, found: 197.2017.

* the neutral (un-protonated) diamine is not air stable and forms an insoluble polymer upon exposure to air, so it must be handled under argon. Once protonated it becomes more stable, but gradually gains a pink colour.

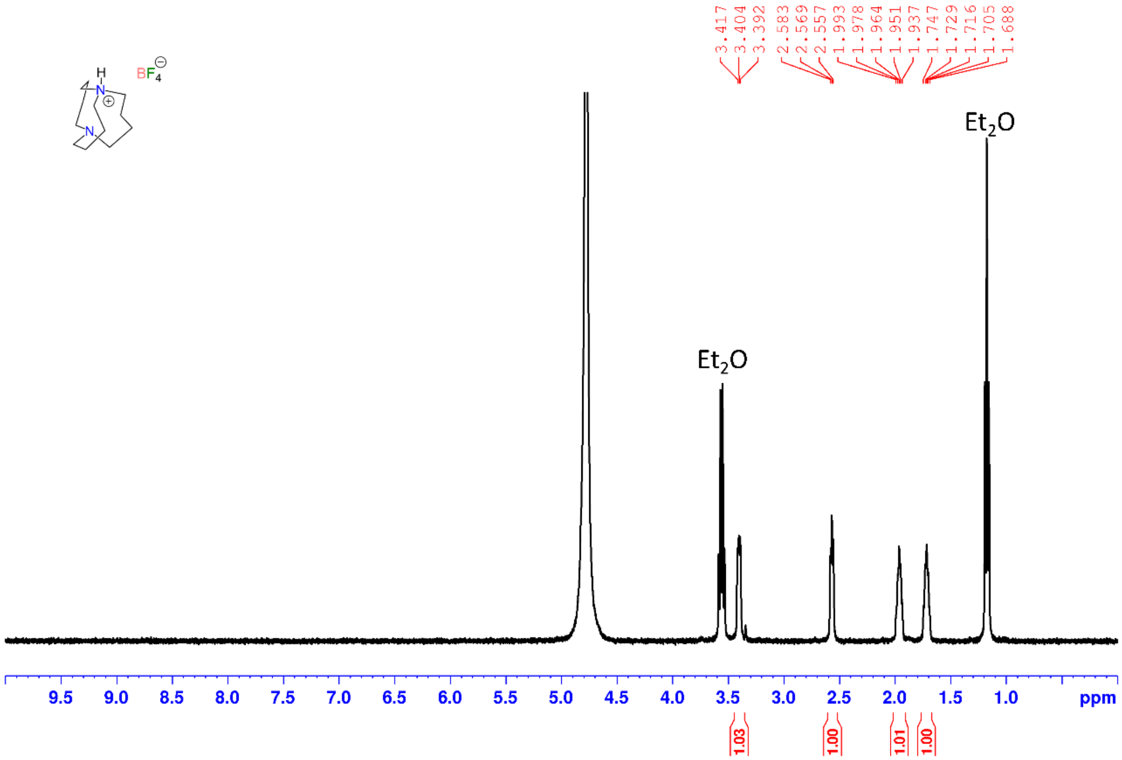

Supplementary Fig 8. ^{1}H NMR spectrum (400 MHz) of 1,6-diazabicyclo[4.4.4]tetradecan-1-ium tetrafluoroborate in D_2O. Residual diethyl ether solvent signals marked by Et_2O.

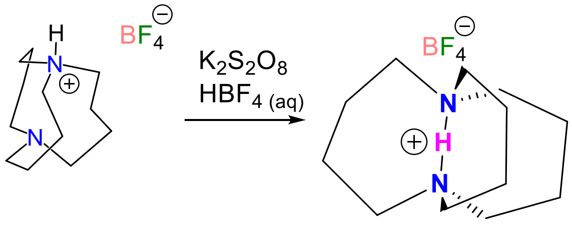

<u>1,6-diazabicyclo[4.4.4]tetradecan-1,6-ium [inside proton] tetrafluoroborate:</u>[1]

In a Schlenk flask, the filtered crude mixture from the previous reaction, nominally the outside protonated BF_4 salt (1.54 g, 5.41 mmol) and 48% HBF_4 (15 mL), was sparged with argon for ~ 10 minutes. Potassium persulfate (1.46 g, 5.41 mmol) was then added and stirred vigorously for 2 hours under argon. The mixture was diluted with diethyl ether (96 mL), filtering the precipitated salts, and removing the solvent under reduced pressure. The mixture was then cooled, adding $KHCO_3$ (12.00 g, 119.9 mmol) to precipitate KBF_4, then diluted the mixture in anhydrous ethanol (50 mL) and filtered. The solvent was removed, extracting the product in anhydrous ethanol and removing the solvent under reduced pressure. The bicarbonate salt was too hygroscopic to conveniently handle, so it was converted back into the BF_4 form by dissolving the product in anhydrous ethanol (1 mL) and adding 48% HBF_4 (0.7 mL), stirring for 1 h. The product was diluted with diethyl ether (50 mL) and decanted the liquid. The product was finally recrystallized from minimal boiling anhydrous ethanol (~ 8 mL), cooling to -20 °C overnight.

The precipitate was filtered, washing with minimal cold ethanol, then diethyl ether to obtain the product as a white powder (1.10 g, 3.89 mmol, 72% yield). 1H NMR (400 MHz, D_2O): δ 16.98 (s, 1H), 2.67 (broad s, 12H), 1.87 (broad s, 12H). 13C NMR (101 MHz, D_2O): δ 51.71, 25.95. 19F NMR (376 MHz, D_2O): δ -150.43 (s, 0.2F; 10B abundance), -150.48 (s, 0.8F; 11B abundance). HRMS (m/z) [M]+ calcd. for C12H25N2: 197.2012, found: 197.2011.

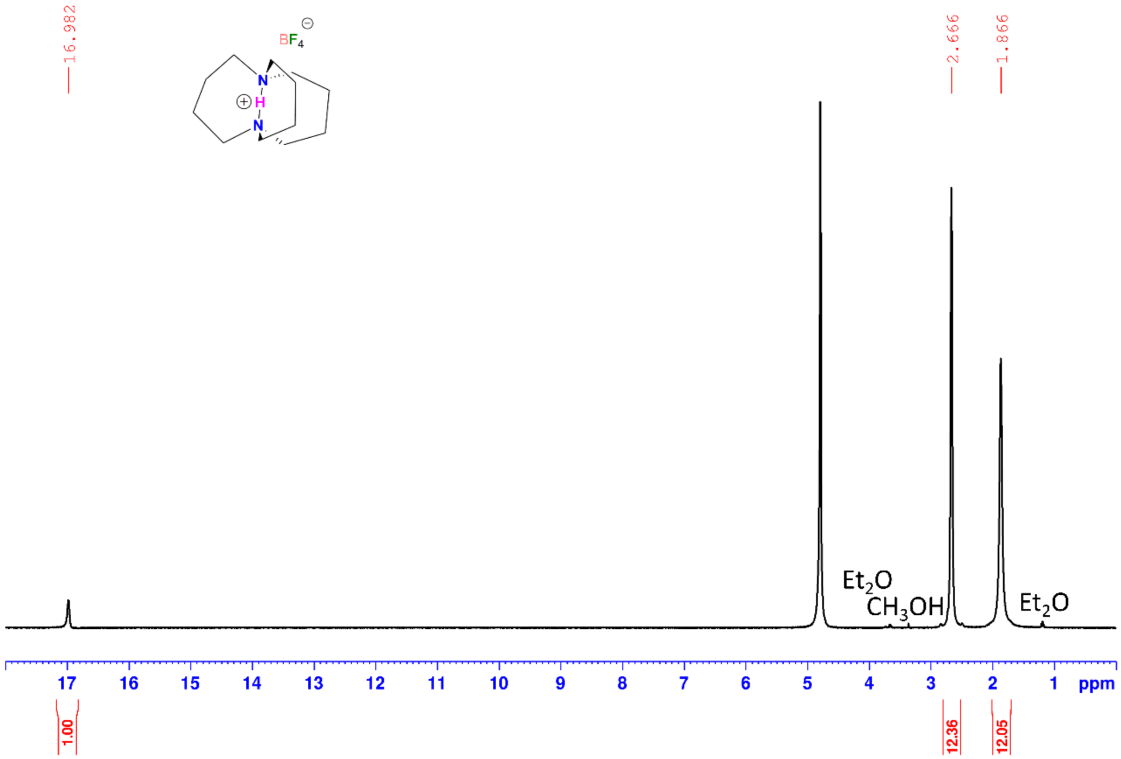

Supplementary Fig 9. ^{1}H NMR spectrum (400 MHz) of 1,6-diazabicyclo[4.4.4]tetradecan-1,6-ium [inside proton] tetrafluoroborate in D_2O. Residual diethyl ether and methanol solvent signals marked by Et_2O and CH_3OH, respectively.

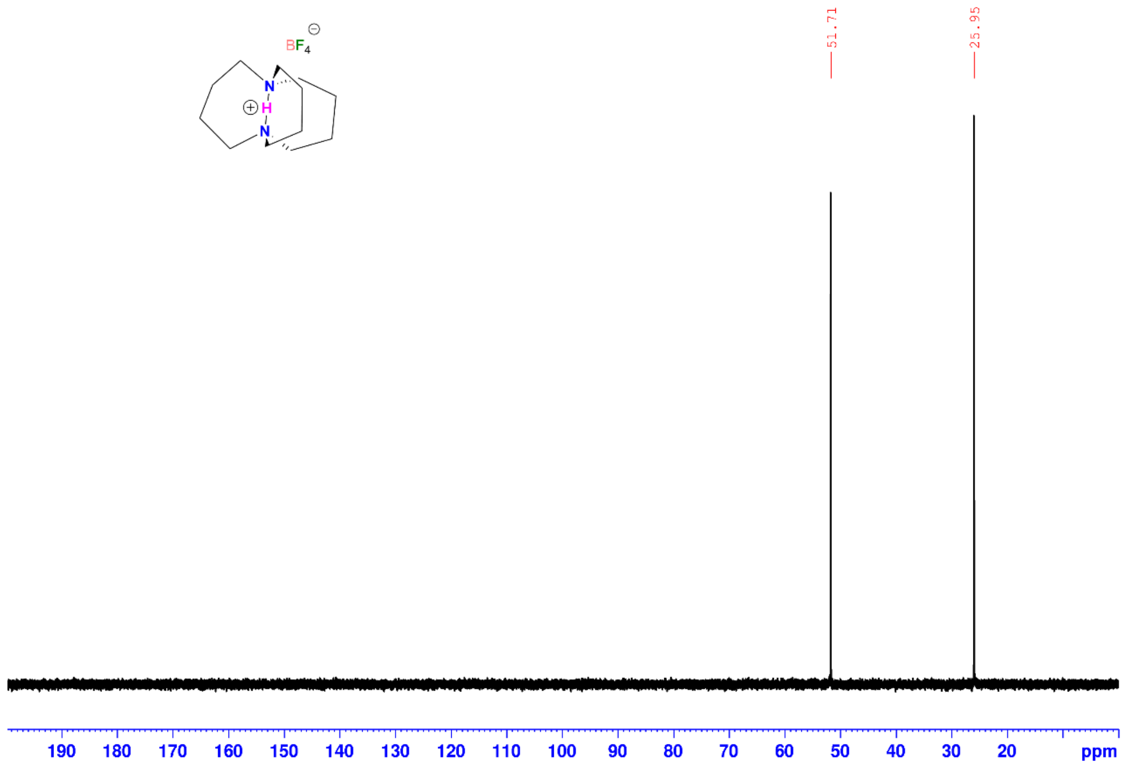

Supplementary Fig 10. ^{13}C {^{1}H} NMR spectrum (101 MHz) of 1,6-diazabicyclo[4.4.4]tetradecan-1,6-ium [inside proton] tetrafluoroborate in D_2O.

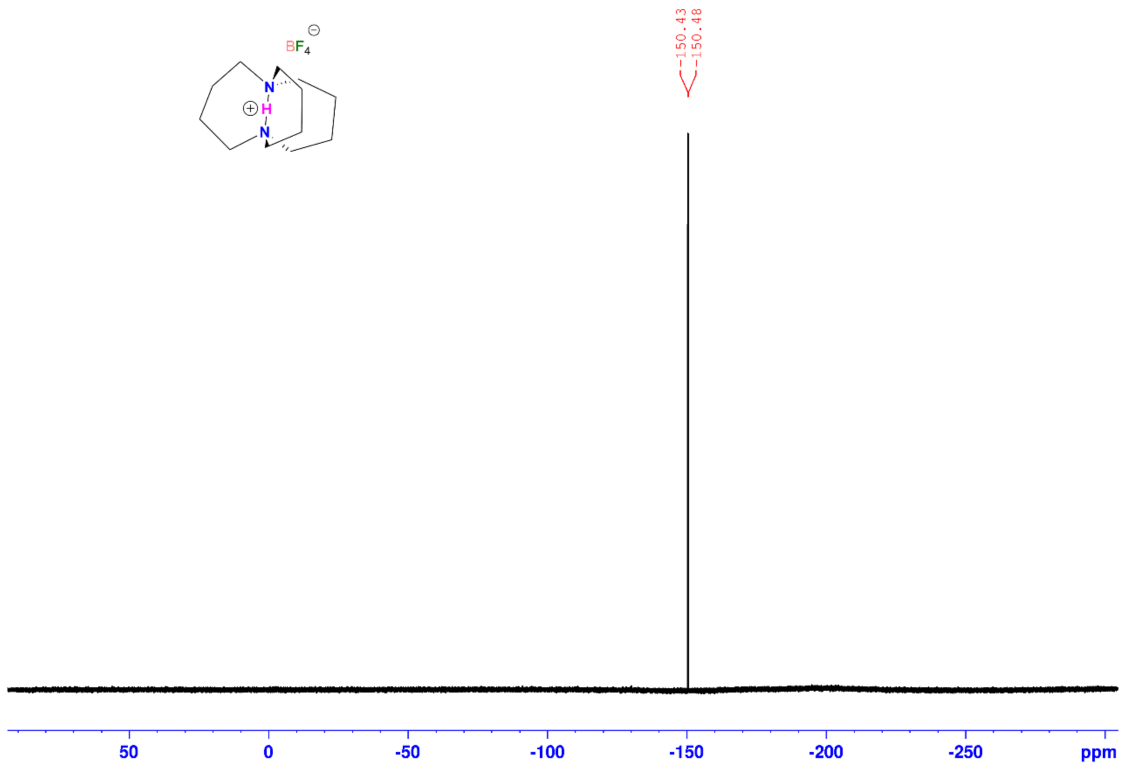

Supplementary Fig 11. ^{19}F NMR spectrum (376 MHz) of 1,6-diazabicyclo[4.4.4]tetradecan-1,6-ium [inside proton] tetrafluoroborate in D_2O.

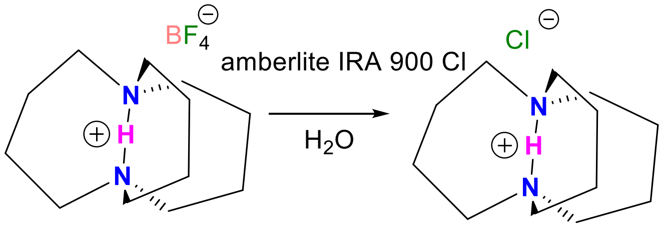

<u>1,6-diazabicyclo[4.4.4]tetradecan-1,6-ium [inside proton] chloride:</u>

1,6-diazabicyclo[4.4.4]tetradecan-1,6-ium [inside proton] tetrafluoroborate (0.50 g, 1.76 mmol) was dissolved in Milli-Q water (10 mL) and passed through a column of amberlite IRA 900-Cl form ion exchange resin (3.5 g), recycling the solution 3 times through the column, until no more BF_4 signals were observed by ^{19}F NMR. The solvent was removed under reduced pressure, extracted in ethanol, and filtered, removing the solvent under reduced pressure. The solid was triturated in diethyl ether, and filtered, collecting the product as a white powder (0.35 g, 1.5 mmol, 85% yield). 1H NMR (400 MHz, CD_3OD): δ 16.95 (s, 1H), 2.70 (broad s, 12H), 1.91 (broad s, 12H). 13C NMR (101 MHz, D_2O): δ 53.08, 27.32.

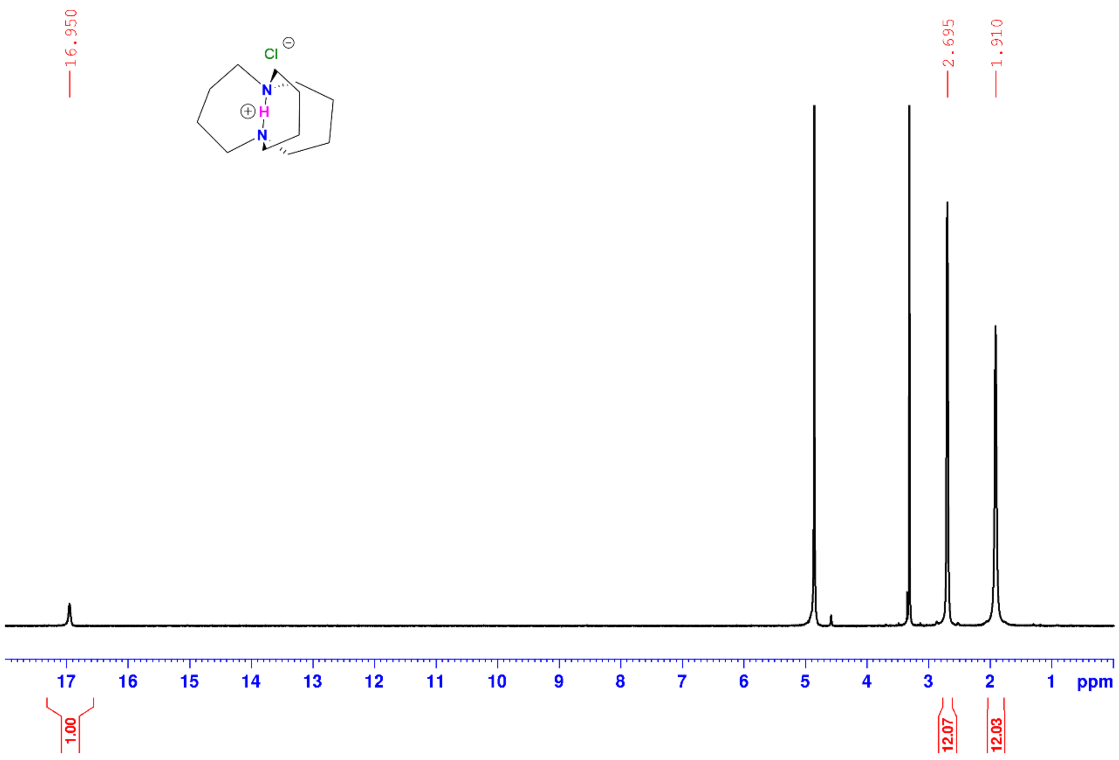

Supplementary Fig 12. ^{1}H NMR spectrum (500 MHz) of 1,6-diazabicyclo[4.4.4]tetradecan-1,6-ium [inside proton] chloride in CD_3OD.

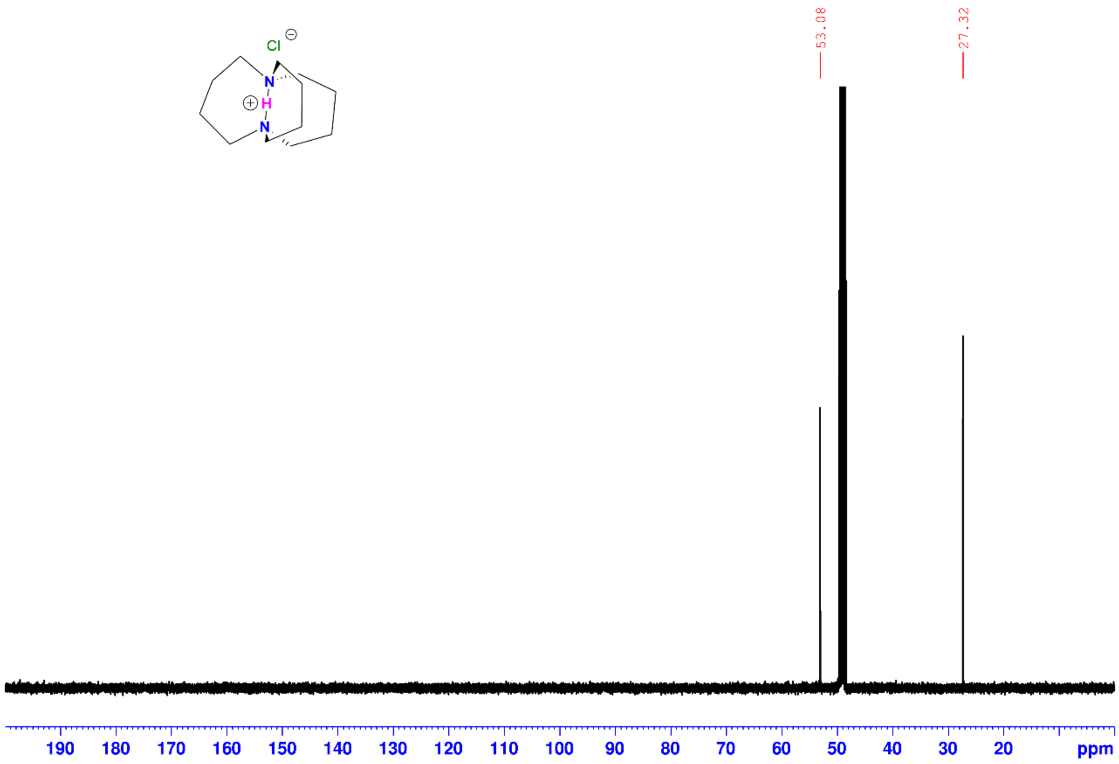

Supplementary Fig 13. ^{13}C {^{1}H} NMR spectrum (101 MHz) of 1,6-diazabicyclo[4.4.4]tetradecan-1,6-ium [inside proton] chloride in CD_3OD.

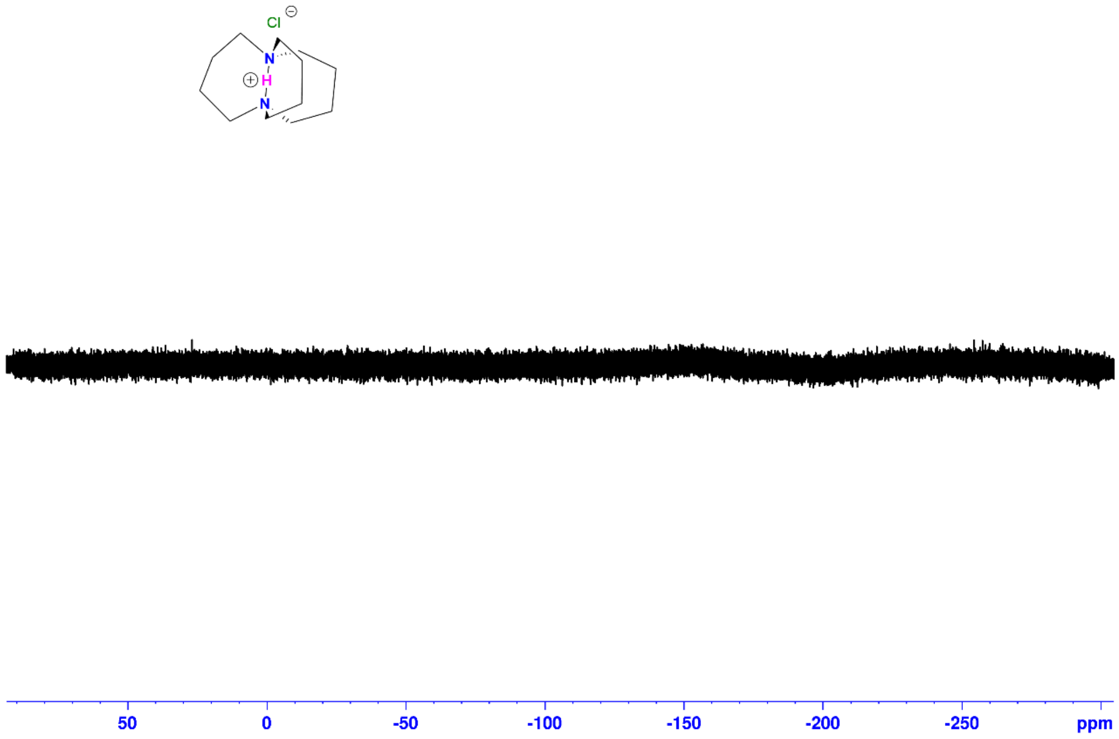

Supplementary Fig 14. ^{19}F NMR spectrum (376 MHz) of 1,6-diazabicyclo[4.4.4]tetradecan-1,6-ium [inside proton] chloride in CD_3OD.

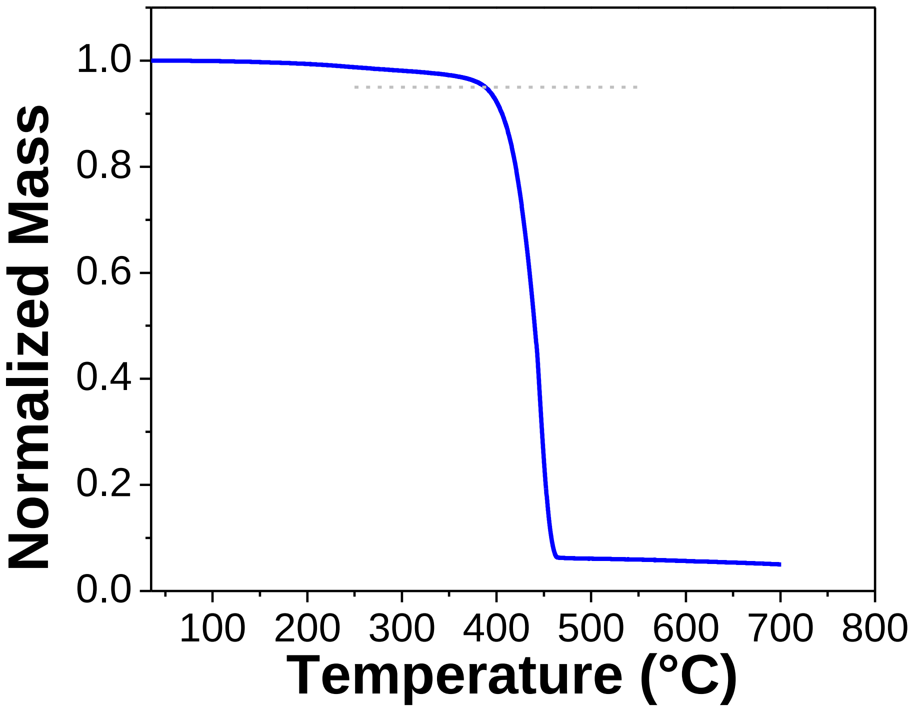

Supplementary Fig 15. Thermogravimetric analysis of **in-DBD** Cl under nitrogen atmosphere at a ramp speed of 10 °C per minute. Grey line indicates 95% of the original mass, which defines the approximated degradation temperature of 388 °C.

<u>6-azaspiro[5.5]tetradec-6-ium bromide:</u>[5]

In a 2-neck RBF, THF (100 ml) was sparged with argon, then added sodium hydroxide (1.93 g, 33.40 mmol) and piperidine (3.00 mL, 30.31 mmol) and heated to 60 °C while stirring under argon. 1,5-dibromopentane (4.14 mL, 30.37 mmol) was added dropwise, then stirred at 60 °C for 18 hours under argon. The product was cooled, solvent was removed under reduced pressure, and the product was extracted with boiling isopropanol and hot filtered, then slowly cooled to -20 °C, filtering to obtain the product as colourless crystals (5.65 g, 24.13 mmol, 79% yield). 1H NMR (400 MHz, CD_3OD): δ 3.44 (t, J = 5.83 Hz, 8H), 1.89 (m, 8H), 1.72 (p, J = 5.92 Hz, 4H)

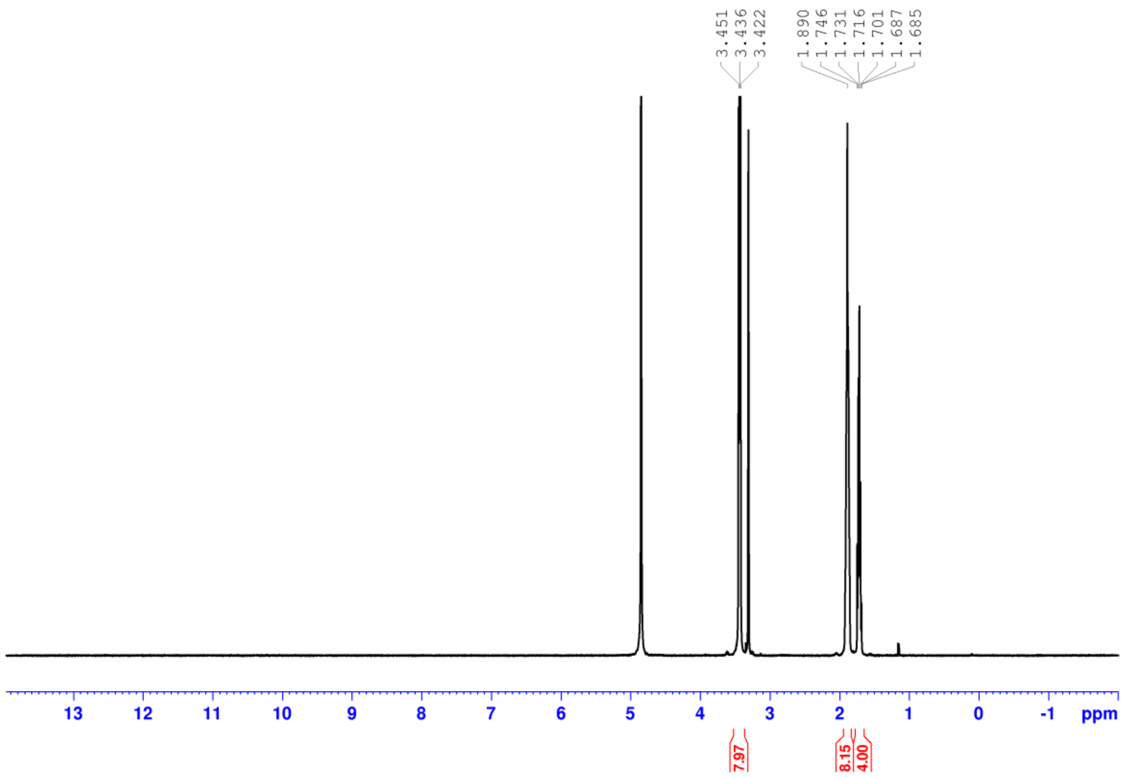

Supplementary Fig 16. ^{1}H NMR spectrum (400 MHz) of 6-azaspiro[5.5]tetradec-6-ium bromide in CD_3OD.

N,N'-dimethyl piperidinium iodide:[5]

In a thick-walled pressure vessel, acetonitrile (40 mL), piperidine (5.00 mL, 50.62 mmol), and potassium carbonate (13.99 g, 101.23 mmol) were stirred for 15 minutes. Methyl iodide (3.23 mL, 51.88 mmol) was added, and the vessel was sealed and heated to 80 °C for 18 hours. The mixture was cooled, and filtered, washing with minimal acetonitrile, then added to a thick-walled pressure vessel. Methyl iodide (3.23 mL, 51.88 mmol) was added and the vessel was sealed, heating to 80 °C for another 6 hours. The solution was cooled, solvent was removed under reduced pressure, and the mixture was triturated in boiling THF. The mixture was slowly cooled to 0 °C and filtered, washing with diethyl ether, then drying in an oven set to 80 °C for 18 hours to obtain the product as a white powder (7.24 g, 30.03 mmol, 59% yield). 1H NMR (400 MHz, CD_3OD): δ 3.36 (t, J = 5.86 Hz, 2H), 3.12 (s, 6H), 1.91 (m, 3H), 1.68 (p, J = 6.08 Hz, 2H).

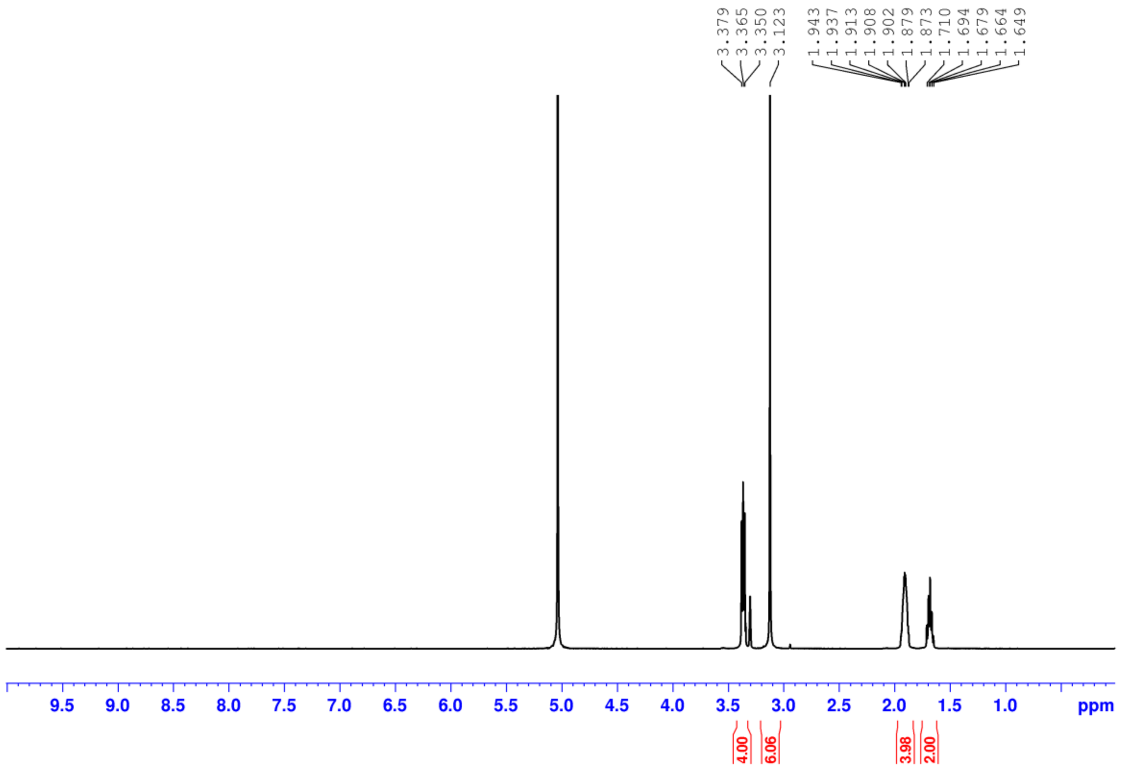

Supplementary Fig 17. ^{1}H NMR spectrum (400 MHz) of N,N'-dimethyl piperidinium iodide in CD_3OD.

2-mesityl-4,5-diphenyl-1H-imidazole:[6]

In thick-walled pressure vessel, benzil (5.00 g, 23.78 mmol), acetic acid (50 mL) and ammonium acetate (18.33 g, 237.80 mmol) was stirred together until mixed, then added mesitaldehyde (4.22 g, 28.54 mmol) and sealed the vessel. The mixture was heated to 120 °C, stirring for 18 hours. The mixture was cooled, and precipitated into ice-cold water (2 L) containing an excess of ammonium hydroxide, stirring for 1 hour. The precipitate was filtered, washing with water. The solid was collected, and triturated with 200 mL of 1M KOH to ensure neutralization and removal of acetate. The solution was again filtered, washing with water and drying overnight to obtain the product as a white powder (7.00 g, 20.68 mmol, 87% yield). 1H NMR (400 MHz, CD_3OD): δ 7.48 (d, J = 7.3 Hz, 4H), 7.38–7.21 (m, 6H), 6.98 (s, 2H), 2.32 (s, 3H), 2.22 (s, 6H).

1,3-dimethyl-2-mesityl-4,5-diphenyl-1H-imidazol-3-ium iodide:[7]

In thick-walled pressure vessel, 2-mesityl-4,5-diphenyl-1H-imidazole (1.00 g, 2.95 mmol) was dissolved in DMSO (10 mL) and added 5M KOH (1 mL), stirring for 10 minutes to yield a

deep red solution. Methyl iodide (0.45 mL, 7.2 mmol) was added, the vessel was sealed, and heated to 80 °C, stirring for 18 hours. The solution was cooled, and precipitated into diethyl ether (1L) under vigorous stirring. The product was filtered and recrystallized from ethanol and water to obtain the product as pale yellow crystals (1.02 g, 2.06 mmol, 69.8 % yield). 1H NMR (400 MHz, CD_3OD): δ 7.56-7.45 (m, 10H), 7.26 (s, 2H), 3.53 (s, 3H), 2.24 (s, 6H).

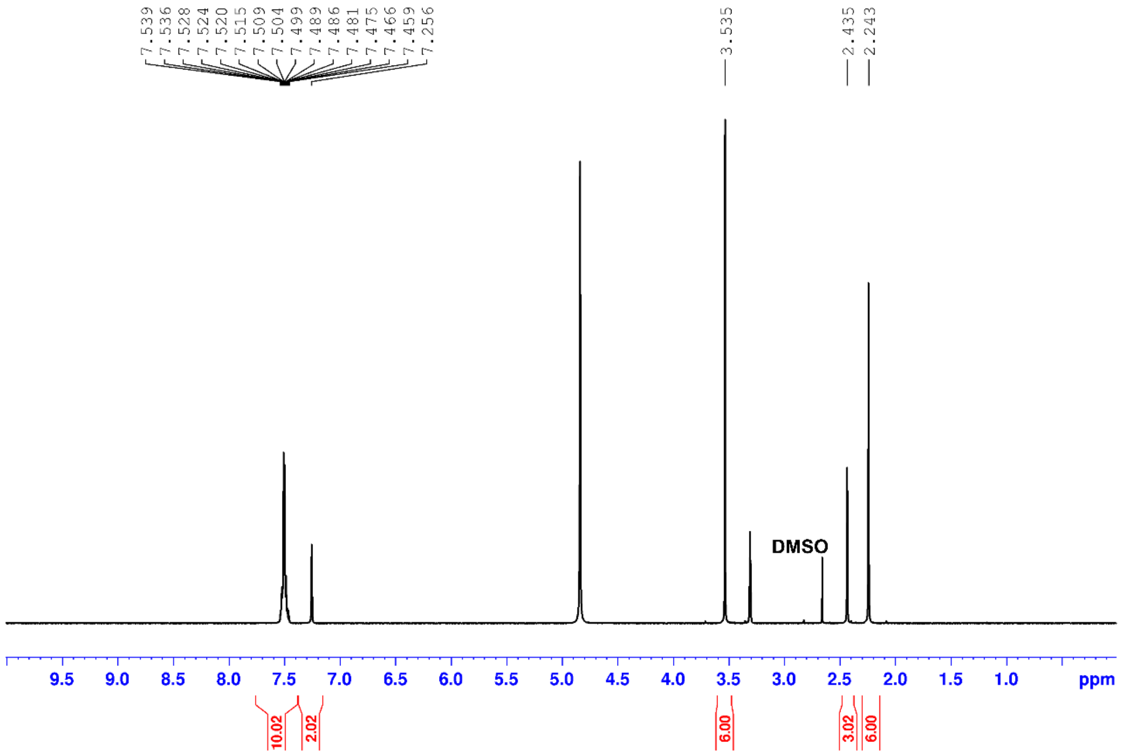

Supplementary Fig 18. ^{1}H NMR spectrum (400 MHz) of 1,3-dimethyl-2-mesityl-4,5-diphenyl-1H-imidazol-3-ium iodide in CD_3OD. Residual dimethyl sulfoxide solvent signal is labeled as DMSO.

<u>NMR degradation study:</u>

The NMR study was adapted from the Coates group.[8] In a nitrogen-atmosphere glovebox*, potassium hydroxide 85% (56 mg), sodium 3-(trimethylsilyl)-1-propanesulfonate (3 mg), and the studied organic cation salt (0.015 mmol) were combined, and then taken out of the glovebox in a sealed air-tight vial. Under a flow of argon*, degassed CD_3OD (0.5 mL) was added to the vial and the vial was sealed under argon and sonicated until all solid dissolved. The resulting solution was transferred under argon to an argon-filled NMR tube, and flame-sealed under a flow of argon. The ^{1}H NMR was then collected for the sample, and then placed in an 80 °C oil bath, removing once every five days to record the next ^{1}H NMR. Samples were allowed to cool for one hour before recording ^{1}H NMR data.

*NMR samples were prepared under argon to limit the water present in the sample to that of the native water present in the KOH pellets (85% m/m, $\lambda \approx 0.55$) and commercial CD_3OD (99%), as well as to exclude CO_2 to limit carbonation of the hydroxide. Under these strictly controlled conditions, we observe much higher rates of decomposition than the previously published studies,[8,9] likely due to a much lower hydration number in this study. Rough estimates for the hydration number in these conditions range from $\lambda \approx 0.88$ (if no excess hydration or carbonation was present) to $\lambda \approx 1.75$ (if KOH was twice as hydrated as expected); this is a similar hydration number to the critical conditions in the DVS around 10% RH.

NMR were referenced to the trimethylsilyl signal of the internal standard, NaDSS, at 0 ppm. All signals of the NaDSS were integrated, setting the signal at 0.595 ppm to 100. All signal intensities were divided by the number of protons represented, for example the signal at 0.595 ppm is a methylene substituent and was therefore divided by 2, resulting in a defined per-proton integration intensity of 50. The integrations of the analyte were also performed, only on signals that were present in the initial NMR (Day 0); these were also divided by the number of protons represented, resulting in a per proton intensity of the analyte. Each signals per-proton intensity was averaged amongst the molecule, and outliers were tested by measuring if the integrated intensity was more than 20% less intense than the average (without the suspected outlier). Outliers were assumed to have considerable deuterium exchange, and were rejected.

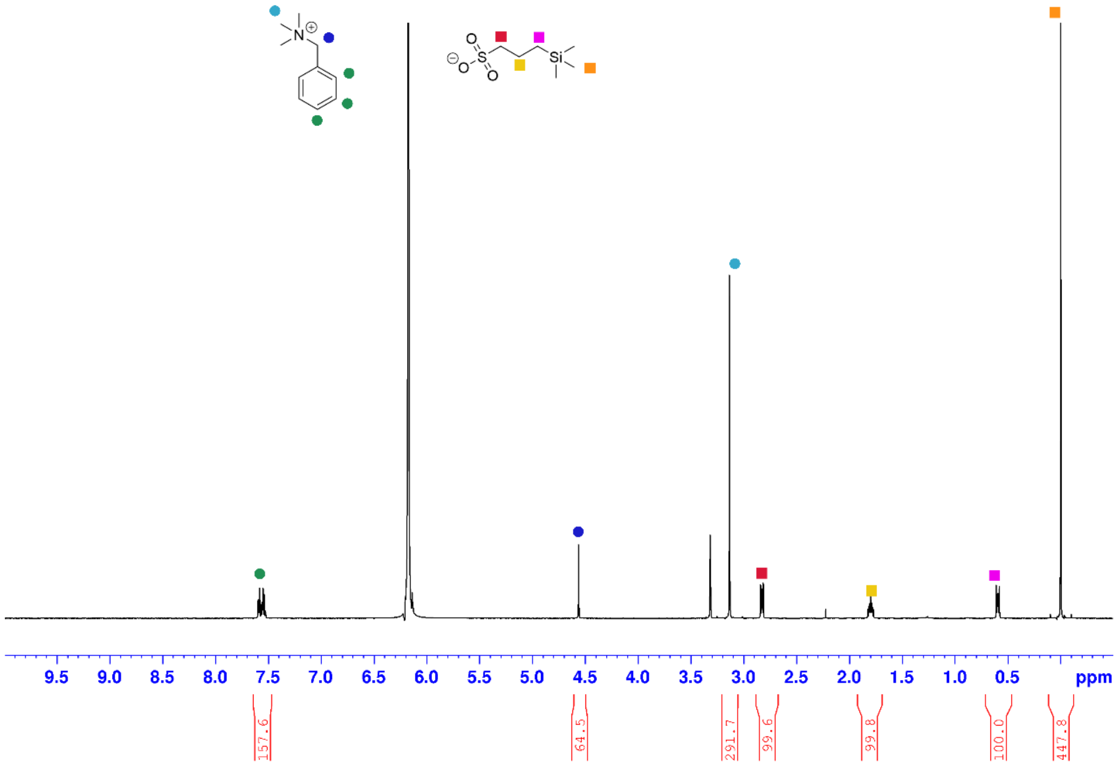

Supplementary Fig 19. ^{1}H NMR spectra (600 MHz) of BTMA on day 0 of the NMR degradation test. Colored circles show proton signals from BTMA and the colored squares show proton signals from the NaDSS internal standard.

Supplementary Tab 1. NMR hydroxide degradation study example integration and averaging to obtain per-proton and molecular unit integration averages.

**BTMA**			**NaDSS**		
Integration	protons	Unit integration	Integration	protons	Unit integration
299	9	33.22222	453	9	50.33333
64	2	32	100	2	50
159	5	31.8	100	2	50
			100	2	50
Average:		32.34	Average		50.08

The total averaged per-proton intensity for the analyte molecule was then divided by the total averaged per-proton intensity of the NaDSS internal standard; this number is the relative equivalents of the analyte to NaDSS.

Relative equivalents = 32.34/50.08 = 0.6457

This number was then divided by the day 0 relative equivalents to get a percent of analyte remaining over time.

Supplementary Tab 2. NMR hydroxide stability test example cation remaining calculation.

**Day**	**Relative equivalents**	**Percent cation remaining**
0	0.6457	100
5	0.2577	39.9
10	0.1006	15.6
15	0.0561	8.7
20	0.026295	4.1
25	0.02429	3.8
29	0.007407	1.1

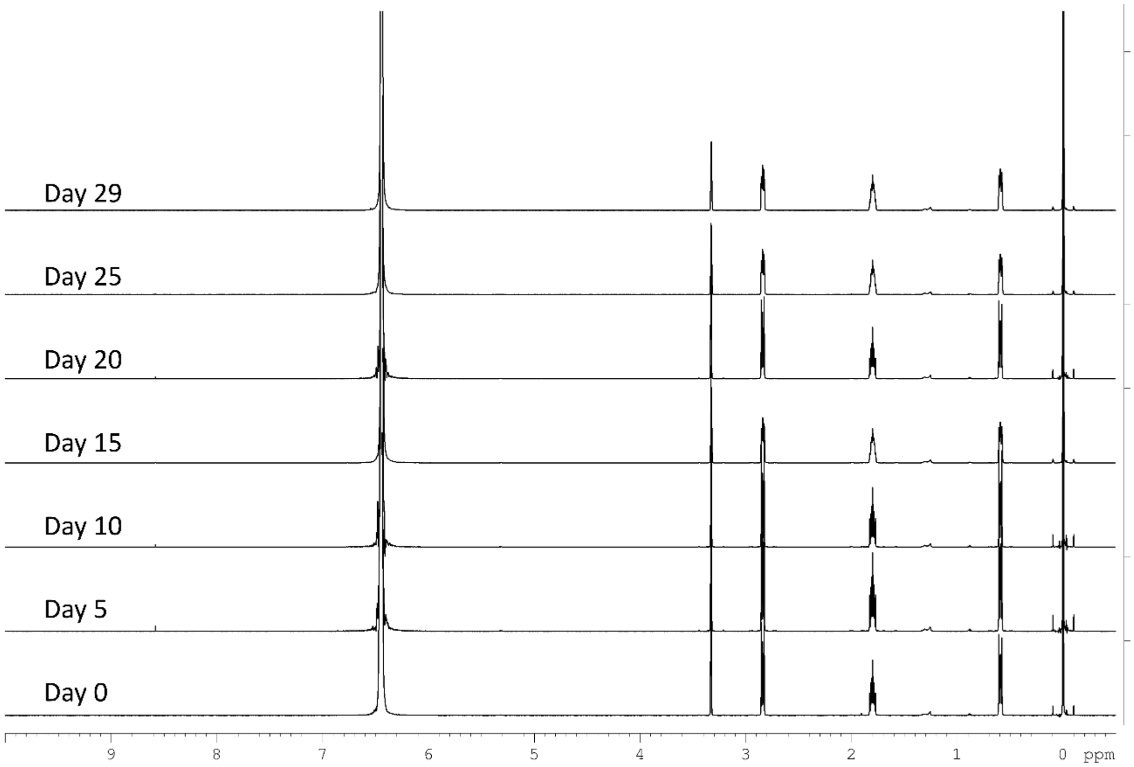

Supplementary Fig 20. Stacked ^{1}H NMR spectra (600 MHz) of negative control experiment; 2M KOH (CD_3OD) with sodium 3-(trimethylsilyl)-1-propanesulfonate (0.03 M) internal standard. NMR tube was heated to 80 °C between samples, allowing to cool before the next spectrum was taken. Peaks around 1.2 ppm are a contaminant found in all tested commercial sources of KOH and NaOH (multiple batches and suppliers), and were found to not change over the course of the experiment and did not appear to interfere with the study.

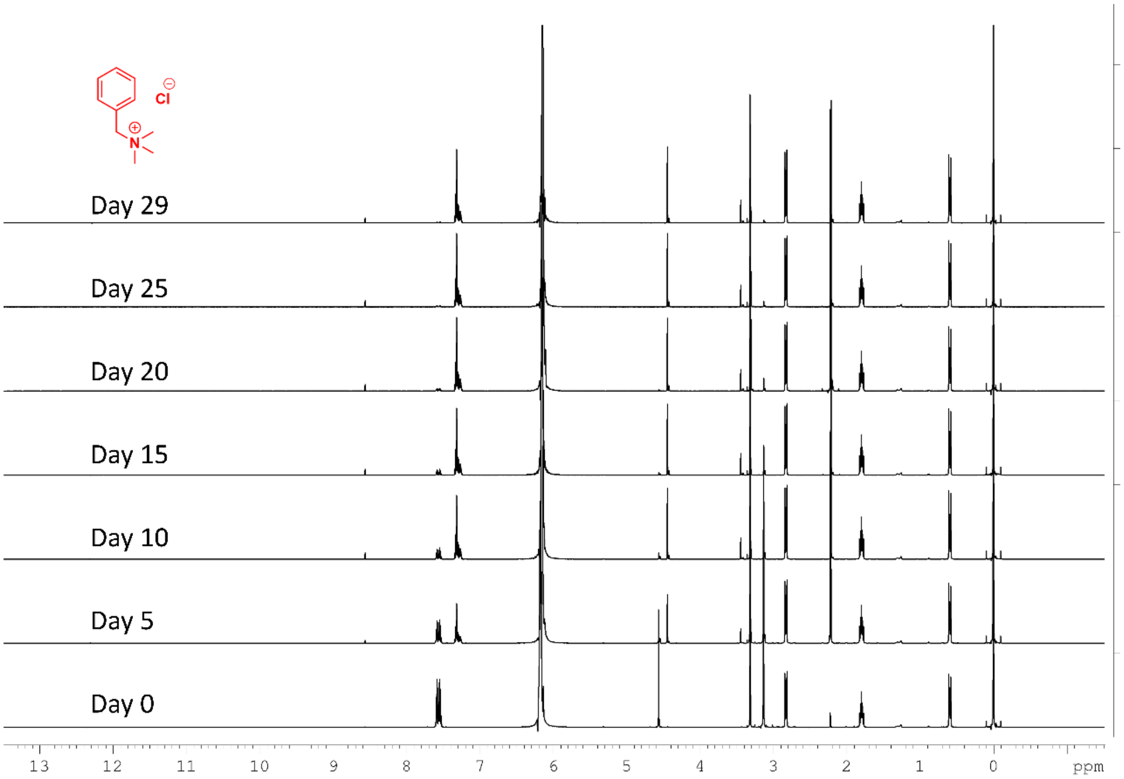

Supplementary Fig 21. Stacked ^{1}H NMR spectra (600 MHz) of benzyltrimethylammonium chloride (0.03 M) in 2M KOH (CD_3OD) with sodium 3-(trimethylsilyl)-1-propanesulfonate (0.03 M) internal standard. NMR tube was heated to 80 °C between samples, allowing to cool before the next spectrum was taken.

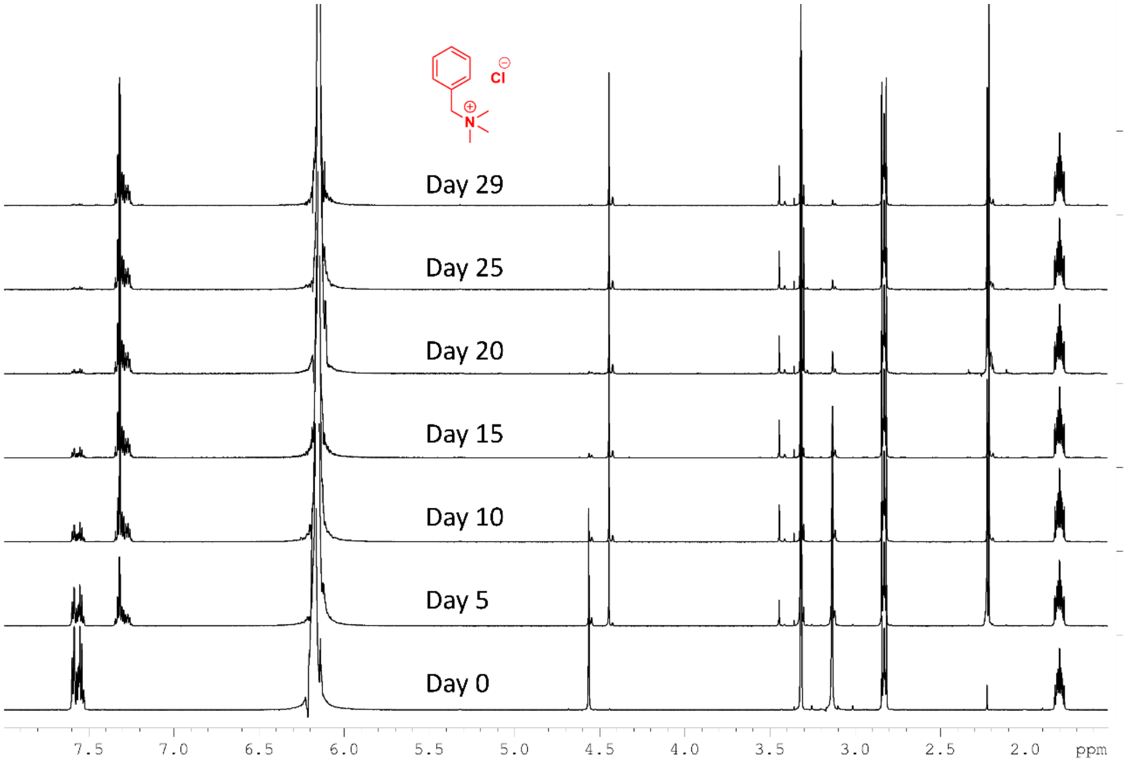

Supplementary Fig 22. Expanded, stacked ^{1}H NMR spectra (600 MHz) of benzyltrimethylammonium chloride (0.03 M) in 2M KOH (CD_3OD) with sodium 3-(trimethylsilyl)-1-propanesulfonate (0.03 M) internal standard. NMR tube was heated to 80 °C between samples, allowing to cool before the next spectrum was taken.

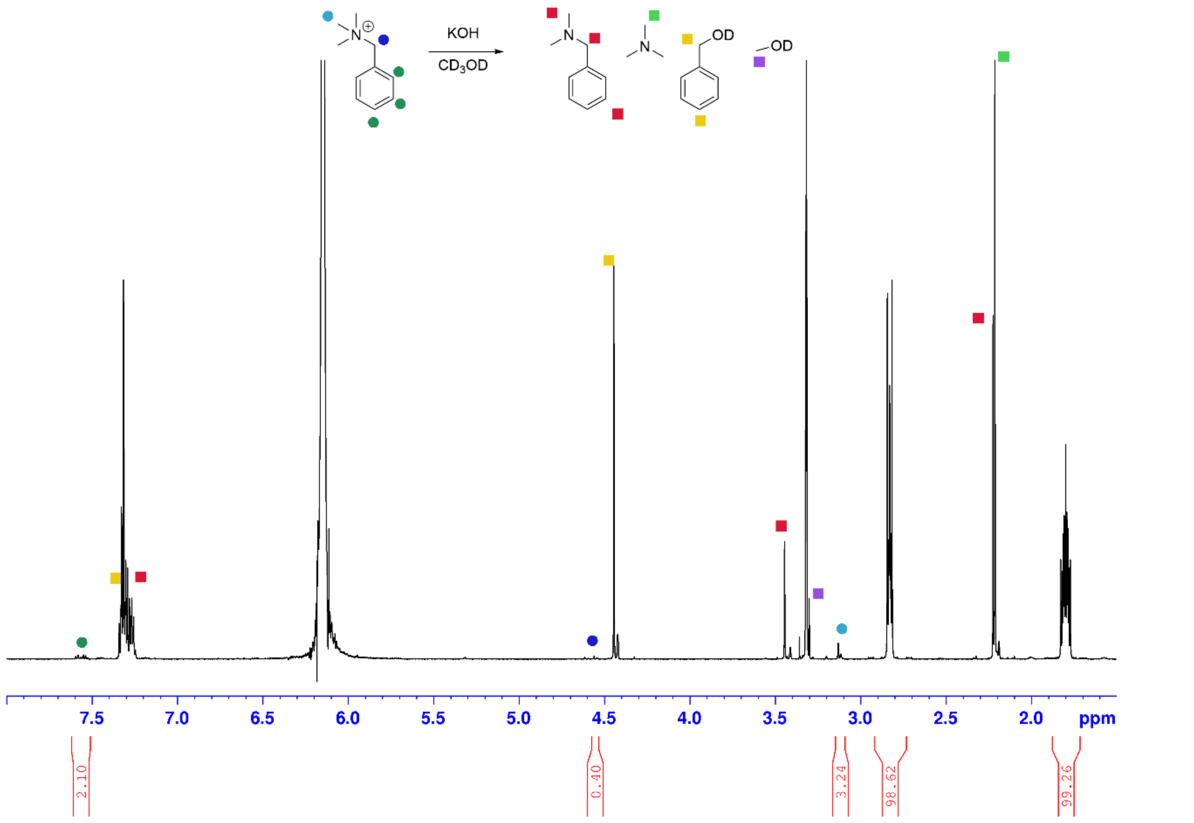

Supplementary Fig 23. Day 29 ^{1}H NMR spectra (600 MHz) of benzyltrimethylammonium chloride (0.03 M) in 2M KOH (CD_3OD) with sodium 3-(trimethylsilyl)-1-propanesulfonate (0.03 M) internal standard. NMR tube was heated to 80 °C for the 29 days. Overlay shows the remaining analyte peaks (colored circles) and the degradation product peaks (colored squares).

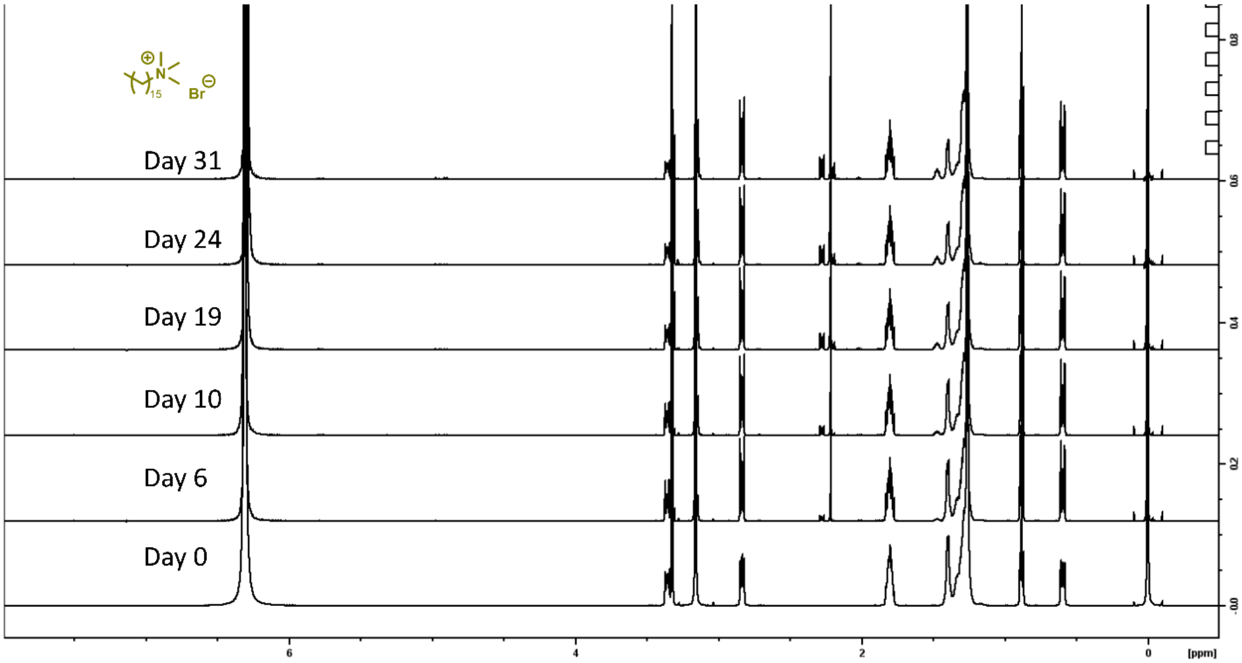

Supplementary Fig 24. Stacked ^{1}H NMR spectra (600 MHz) of hexadecyltrimethylammonium bromide (0.03 M) in 2M KOH (CD_3OD) with sodium 3-(trimethylsilyl)-1-propanesulfonate (0.03 M) internal standard. NMR tube was heated to 80 °C between samples, allowing to cool before the next spectrum was taken.

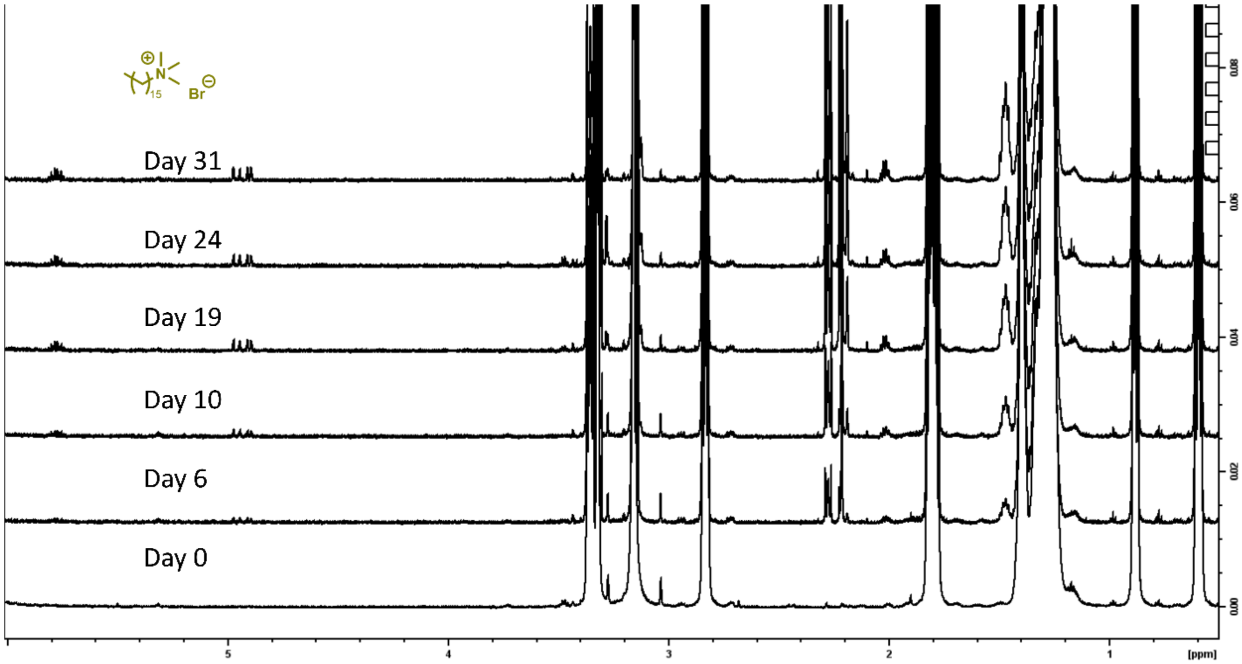

Supplementary Fig 25. Expanded, stacked ^{1}H NMR spectra (600 MHz) of hexadecyltrimethylammonium bromide (0.03 M) in 2M KOH (CD_3OD) with sodium 3-(trimethylsilyl)-1-propanesulfonate (0.03 M) internal standard. NMR tube was heated to 80 °C between samples, allowing to cool before the next spectrum was taken.

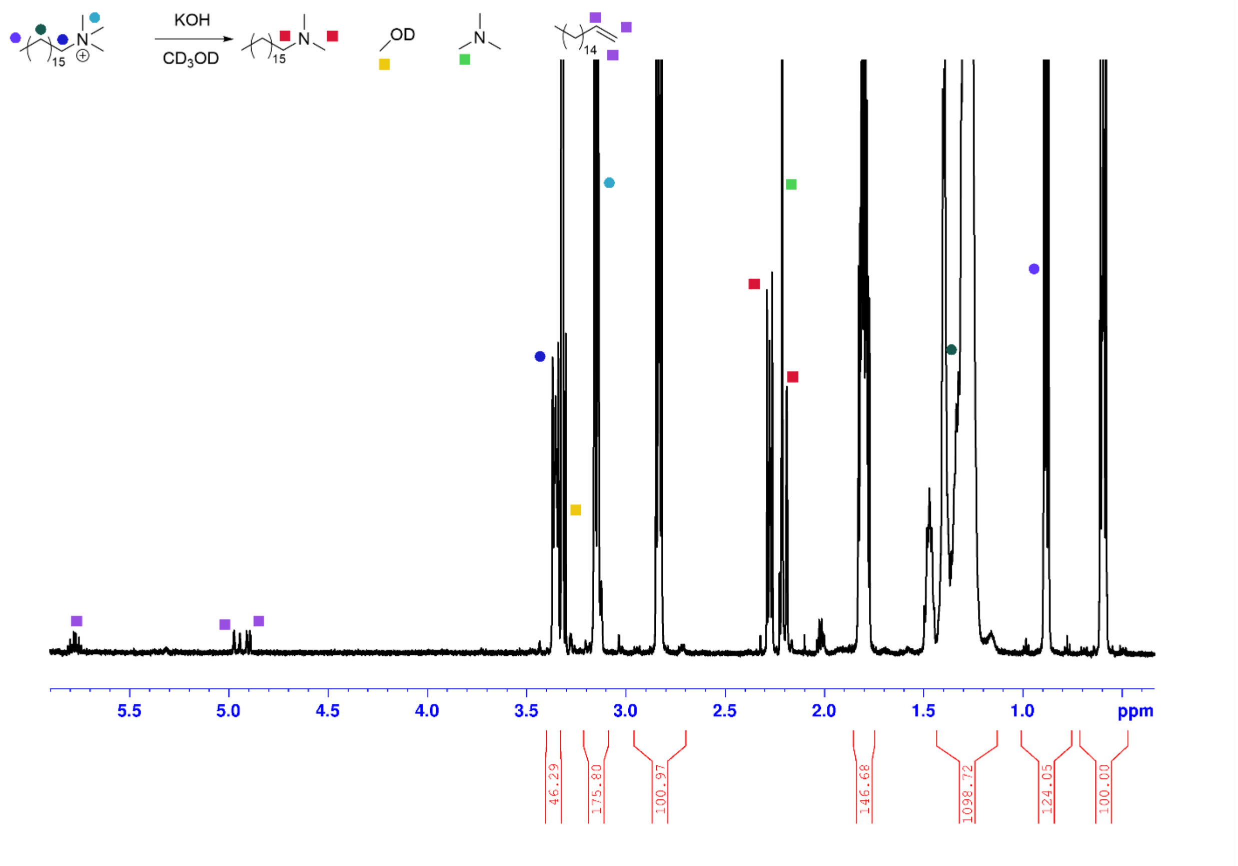

Supplementary Fig 26. Day 31 ^{1}H NMR spectra (600 MHz) of hexadecyltrimethylammonium bromide (0.03 M) in 2M KOH (CD_3OD) with sodium 3-(trimethylsilyl)-1-propanesulfonate (0.03 M) internal standard. NMR tube was heated to 80 °C for the 31 days. Overlay shows the remaining analyte peaks (colored circles) and the degradation product peaks (colored squares).

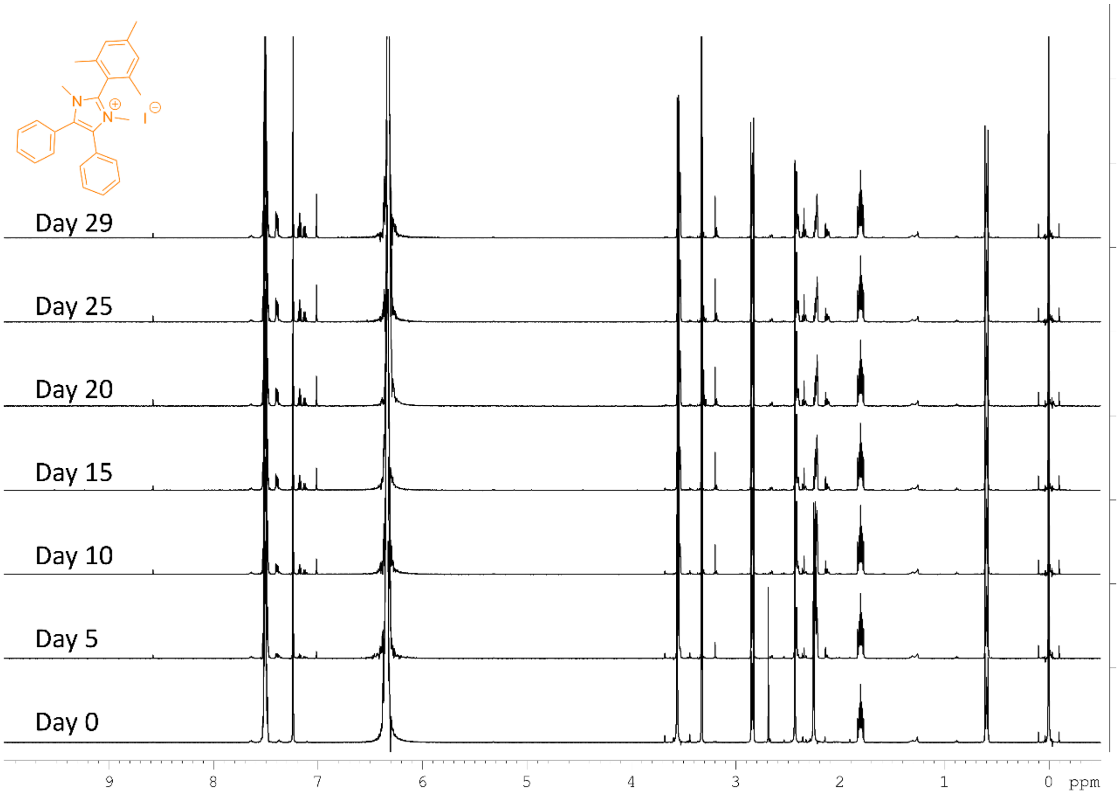

Supplementary Fig 27. Stacked ^{1}H NMR spectra (600 MHz) of 1,3-dimethyl-2-mesityl-4,5-diphenyl-1H-imidazol-3-ium iodide (0.03 M) in 2M KOH (CD_3OD) with sodium 3-(trimethylsilyl)-1-propanesulfonate (0.03 M) internal standard. NMR tube was heated to 80 °C between samples, allowing to cool before the next spectrum was taken.

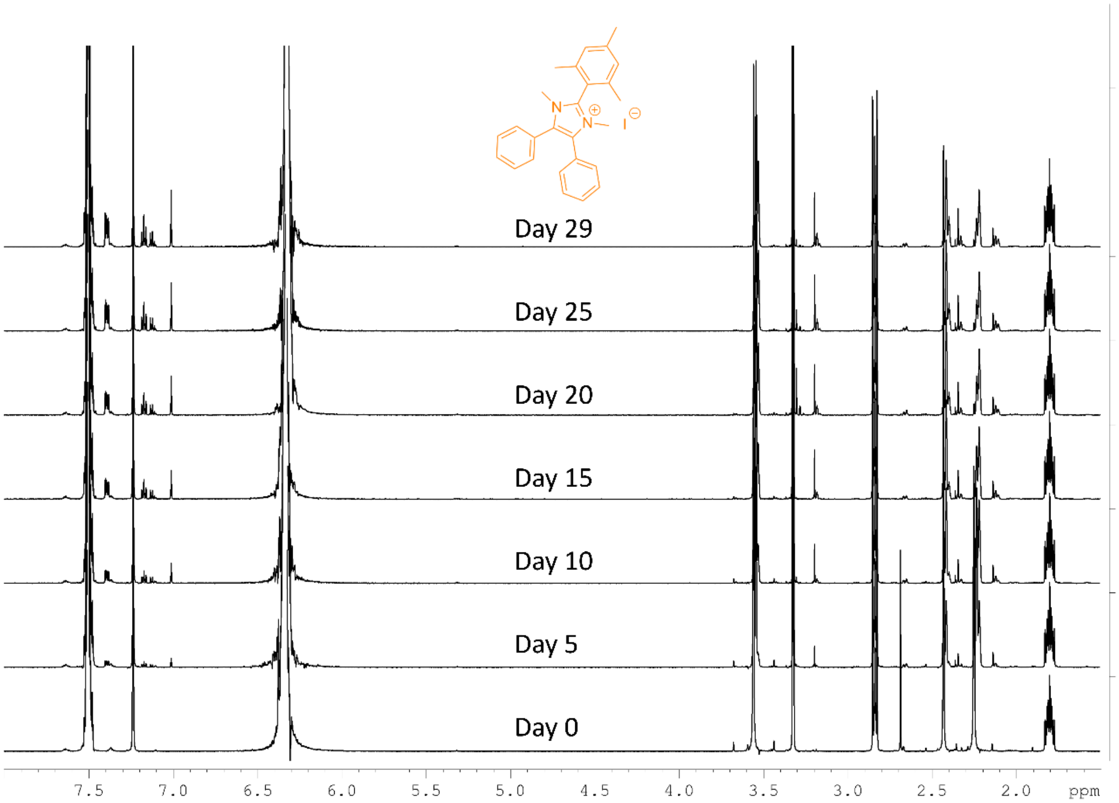

Supplementary Fig 28. Expanded, stacked ^{1}H NMR spectra (600 MHz) of 1,3-dimethyl-2-mesityl-4,5-diphenyl-1H-imidazol-3-ium iodide (0.03 M) in 2M KOH (CD_3OD) with sodium 3-(trimethylsilyl)-1-propanesulfonate (0.03 M) internal standard. NMR tube was heated to 80 °C between samples, allowing to cool before the next spectrum was taken.

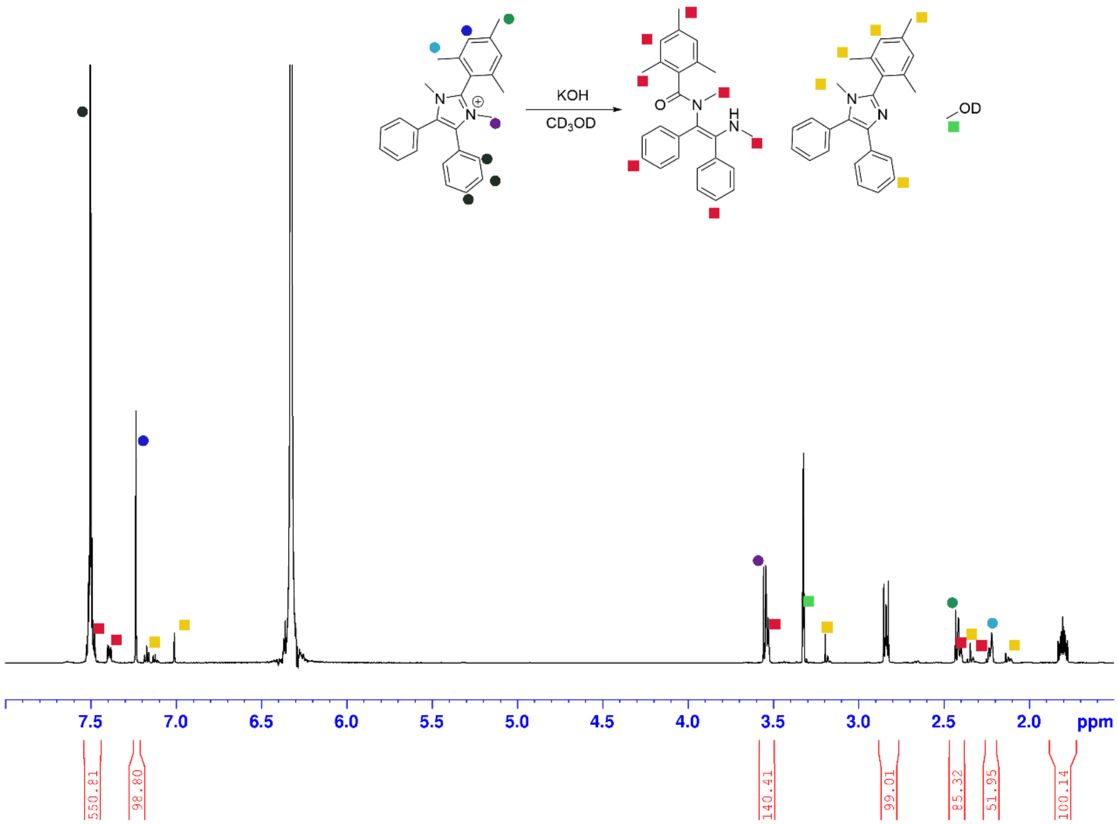

Supplementary Fig 29. Day 29 ^{1}H NMR spectra (600 MHz) of 1,3-dimethyl-2-mesityl-4,5-diphenyl-1H-imidazol-3-ium iodide (0.03 M) in 2M KOH (CD_3OD) with sodium 3-(trimethylsilyl)-1-propanesulfonate (0.03 M) internal standard. NMR tube was heated to 80 °C for the 29 days. Overlay shows the remaining analyte peaks (colored circles) and the degradation product peaks (colored squares).

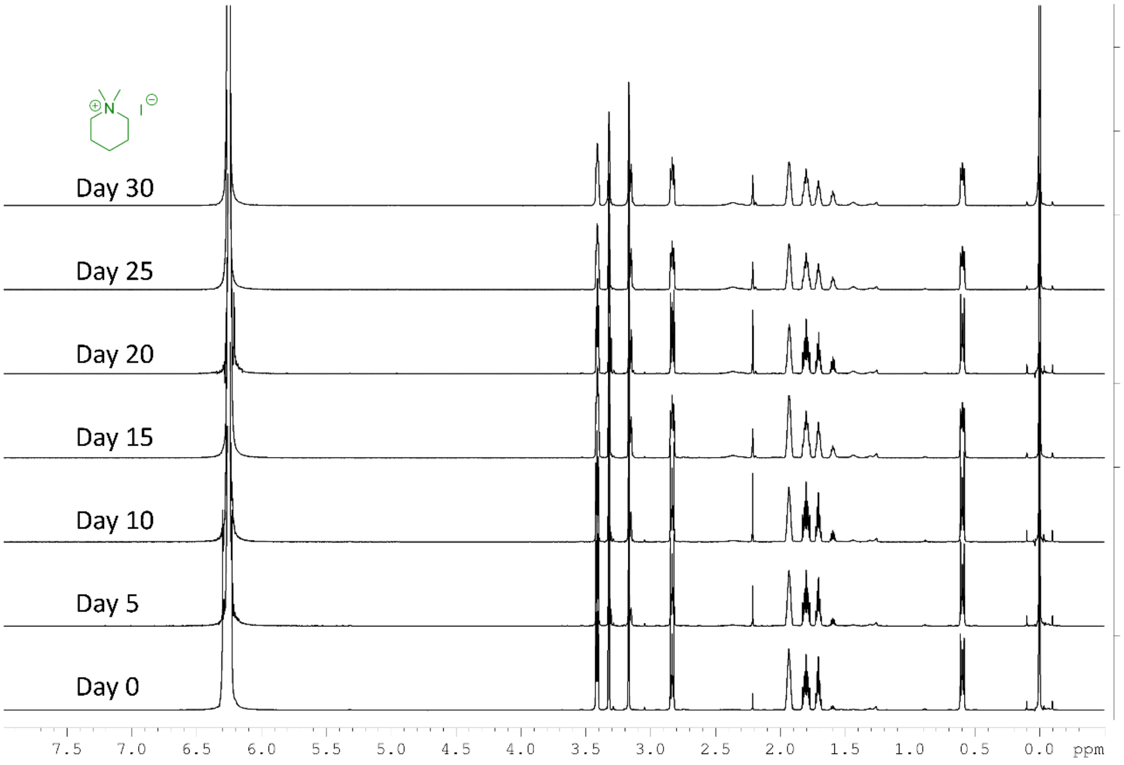

Supplementary Fig 30. Stacked ^{1}H NMR spectra (600 MHz) of 1,1-dimethylpiperidinium iodide (0.03 M) in 2M KOH (CD_3OD) with sodium 3-(trimethylsilyl)-1-propanesulfonate (0.03 M) internal standard. NMR tube was heated to 80 °C between samples, allowing to cool before the next spectrum was taken.

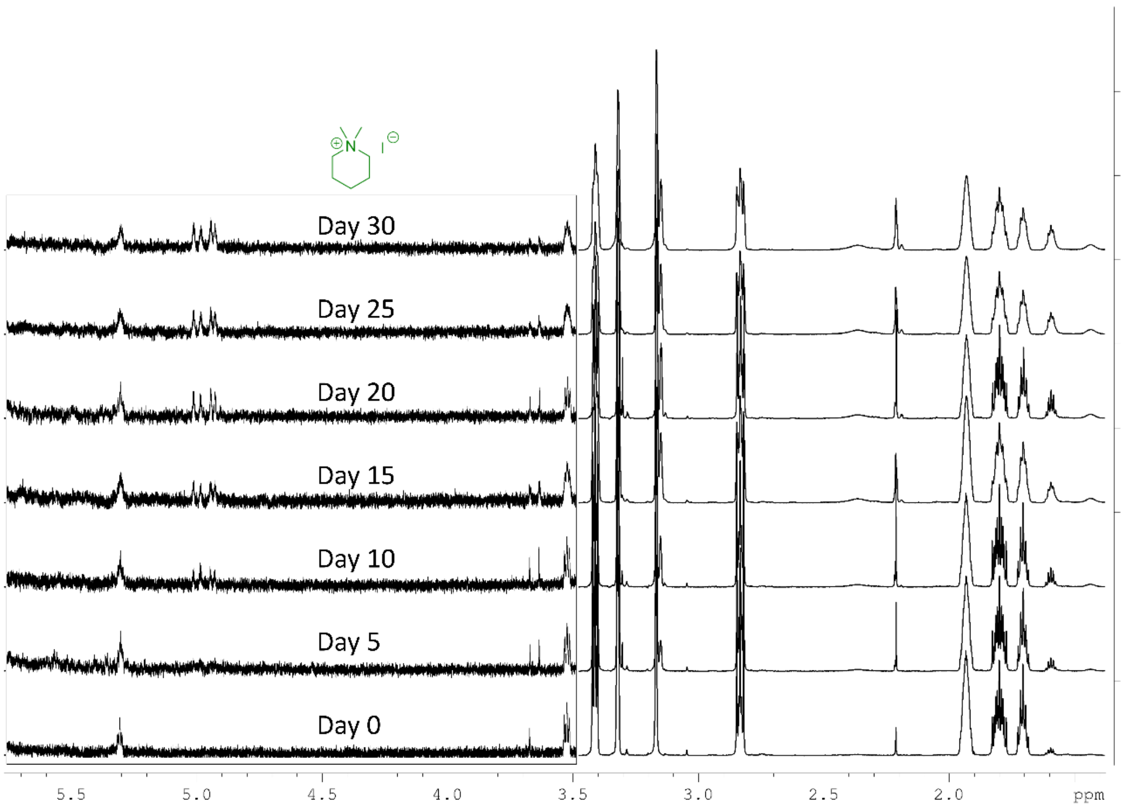

Supplementary Fig 31. Expanded, stacked ^{1}H NMR spectra (600 MHz) of 1,1-dimethylpiperidinium iodide (0.03 M) in 2M KOH (CD_3OD) with sodium 3-(trimethylsilyl)-1-propanesulfonate (0.03 M) internal standard. NMR tube was heated to 80 °C between samples, allowing to cool before the next spectrum was taken. The region in the box was increased in intensity to show the small amount of elimination product that was formed.

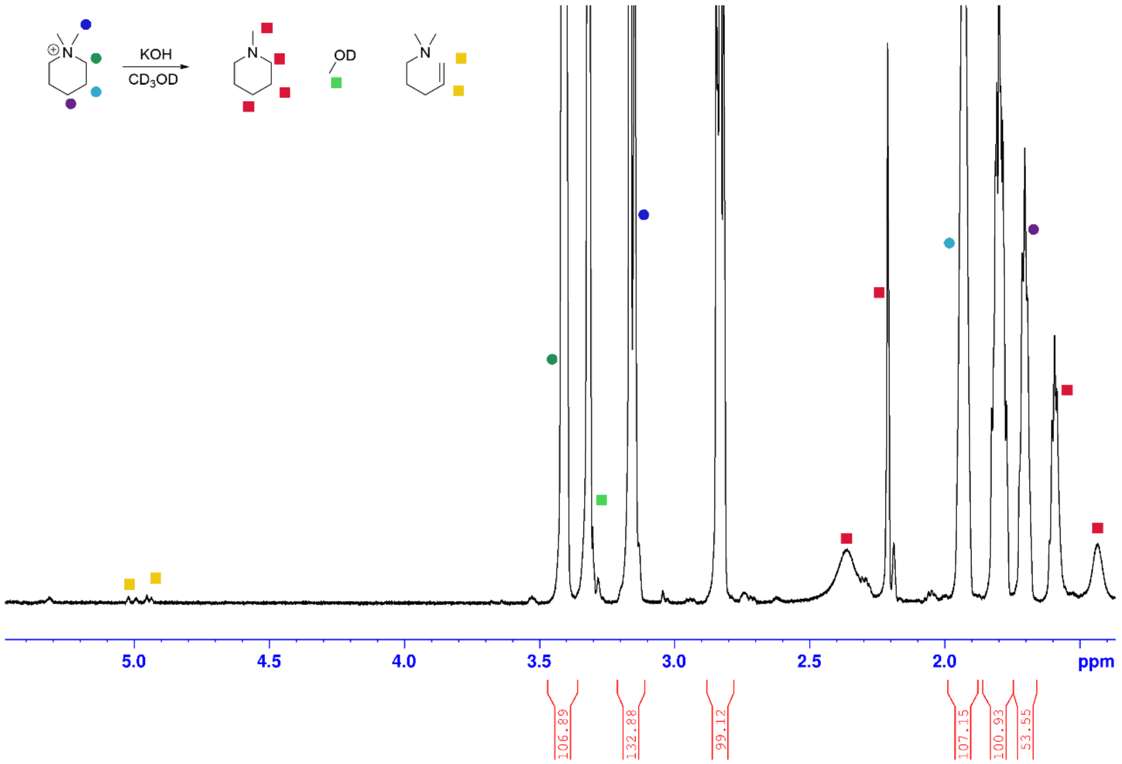

Supplementary Fig 32. Day 30 ^{1}H NMR spectra (600 MHz) of 1,1-dimethylpiperidinium iodide (0.03 M) in 2M KOH (CD_3OD) with sodium 3-(trimethylsilyl)-1-propanesulfonate (0.03 M) internal standard. NMR tube was heated to 80 °C for the 30 days. Overlay shows the remaining analyte peaks (colored circles) and the degradation product peaks (colored squares).

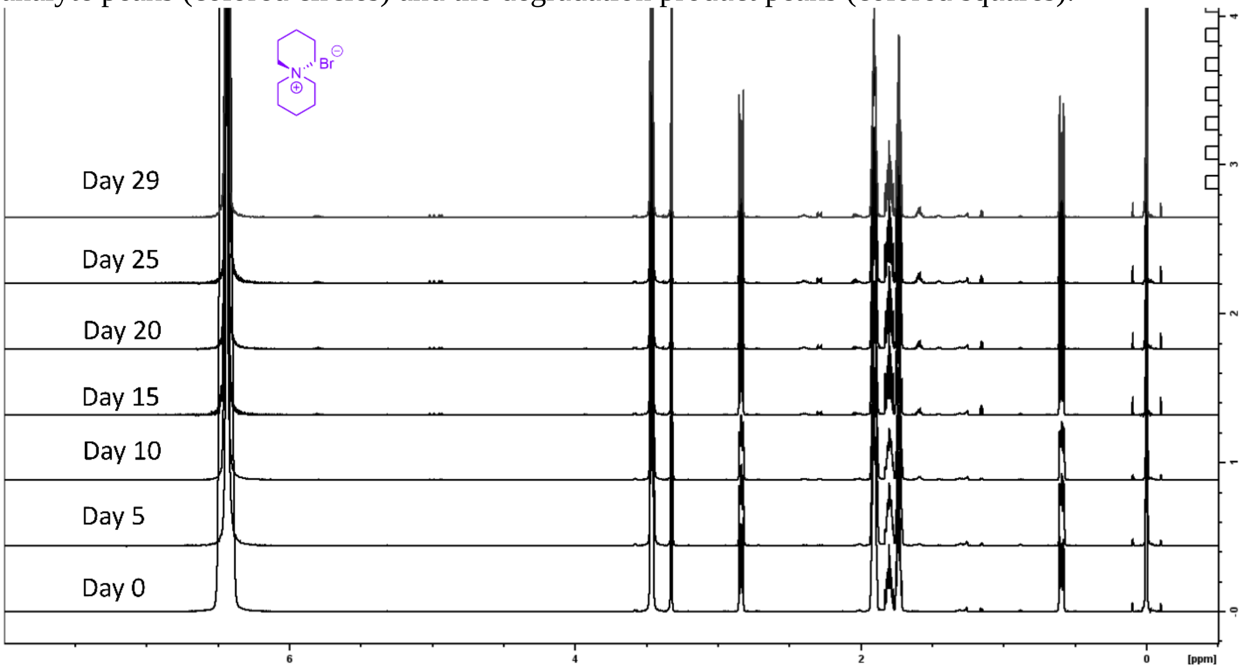

Supplementary Fig 33. Stacked ^{1}H NMR spectra (600 MHz) of 6-azaspiro[5.5]undecan-6-ium bromide (0.03 M) in 2M KOH (CD_3OD) with sodium 3-(trimethylsilyl)-1-propanesulfonate (0.03 M) internal standard. NMR tube was heated to 80 °C between samples, allowing to cool before the next spectrum was taken.

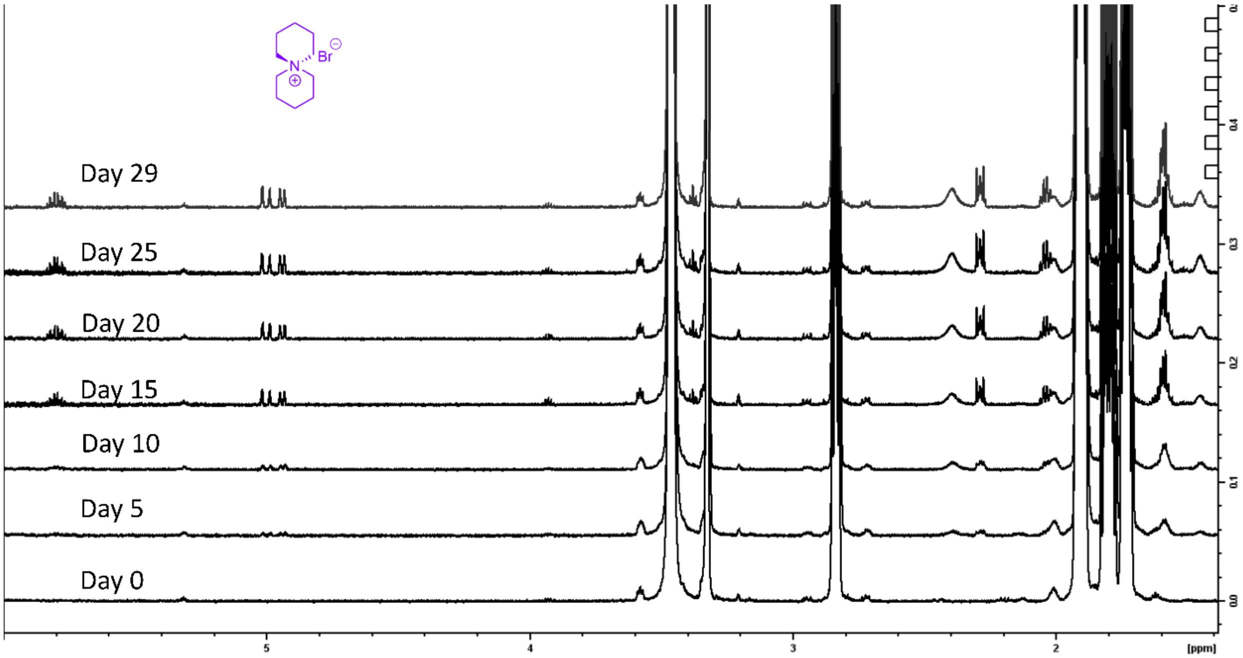

Supplementary Fig 34. Expanded, stacked ^{1}H NMR spectra (600 MHz) of 6-azaspiro[5.5]undecan-6-ium bromide (0.03 M) in 2M KOH (CD_3OD) with sodium 3-(trimethylsilyl)-1-propanesulfonate (0.03 M) internal standard. NMR tube was heated to 80 °C between samples, allowing to cool before the next spectrum was taken.

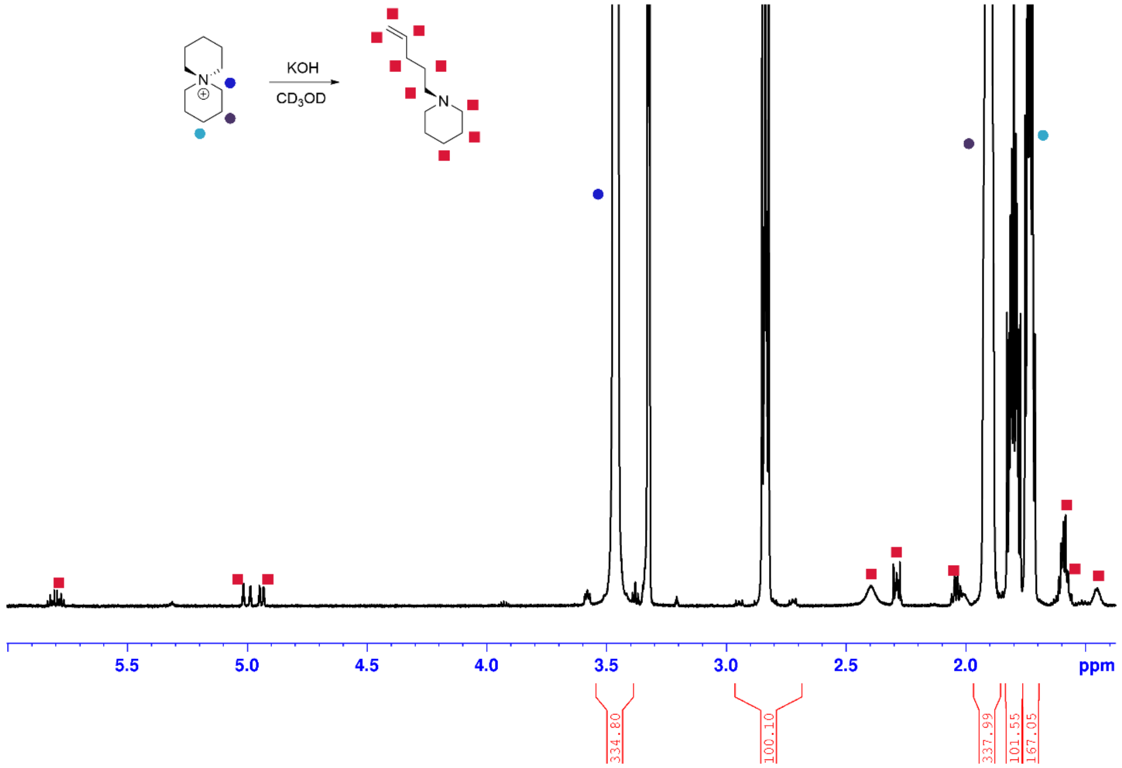

Supplementary Fig 35. Day 29 ^{1}H NMR spectra (600 MHz) of 6-azaspiro[5.5]undecan-6-ium bromide (0.03 M) in 2M KOH (CD_3OD) with sodium 3-(trimethylsilyl)-1-propanesulfonate

(0.03 M) internal standard. NMR tube was heated to 80 °C for the 29 days. Overlay shows the remaining analyte peaks (colored circles) and the degradation product peaks (colored squares).

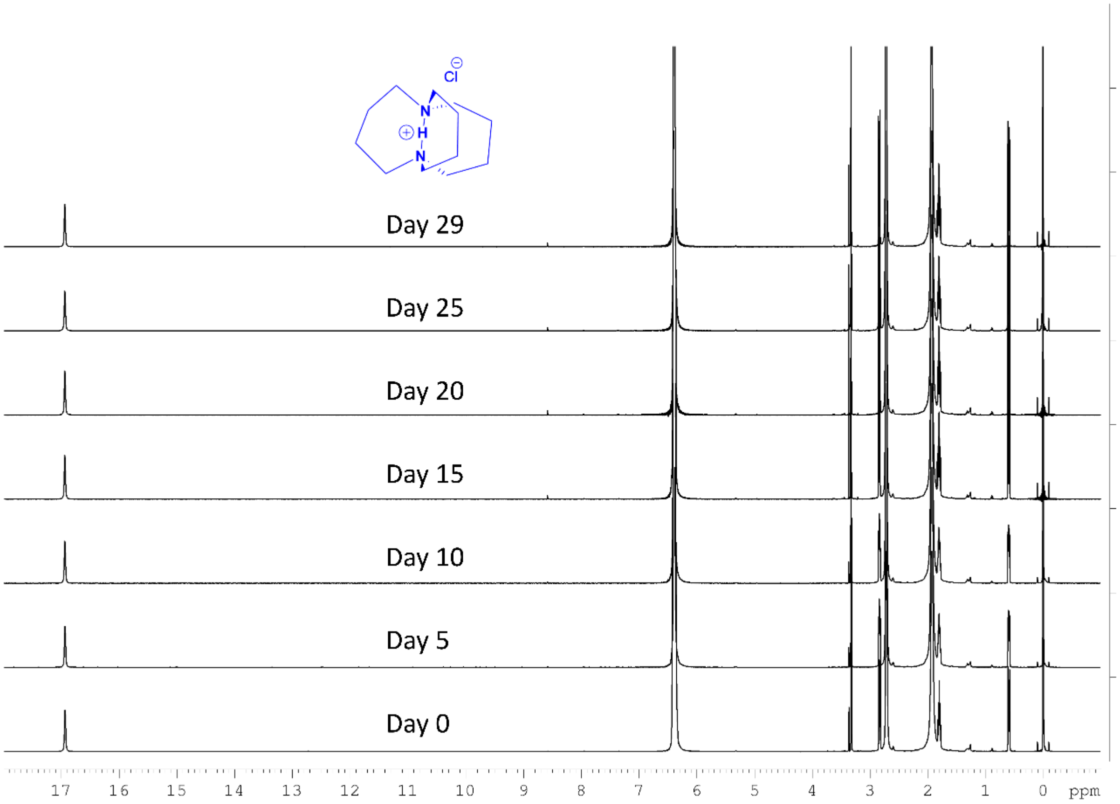

Supplementary Fig 36. Stacked ^{1}H NMR spectra (600 MHz) of 1,6-diazabicyclo[4.4.4]tetradecan-1,6-ium [inside proton] chloride (0.03 M) in 2M KOH (CD_3OD) with sodium 3-(trimethylsilyl)-1-propanesulfonate (0.03 M) internal standard. NMR tube was heated to 80 °C between samples, allowing to cool before the next spectrum was taken.

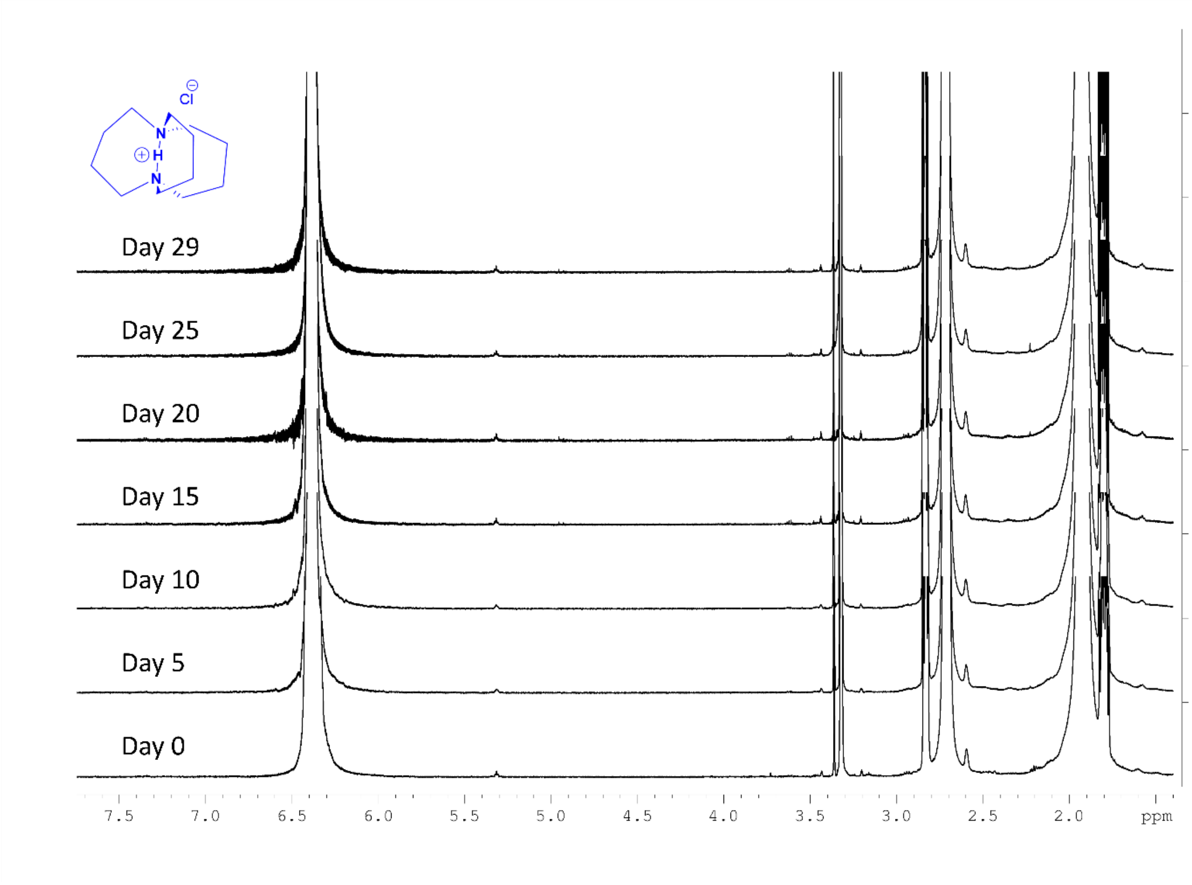

Supplementary Fig 37. Expanded, stacked ^{1}H NMR spectra (600 MHz) of 1,6-diazabicyclo[4.4.4]tetradecan-1,6-ium [inside proton] chloride (0.03 M) in 2M KOH (CD_3OD) with sodium 3-(trimethylsilyl)-1-propanesulfonate (0.03 M) internal standard. NMR tube was heated to 80 °C between samples, allowing to cool before the next spectrum was taken.

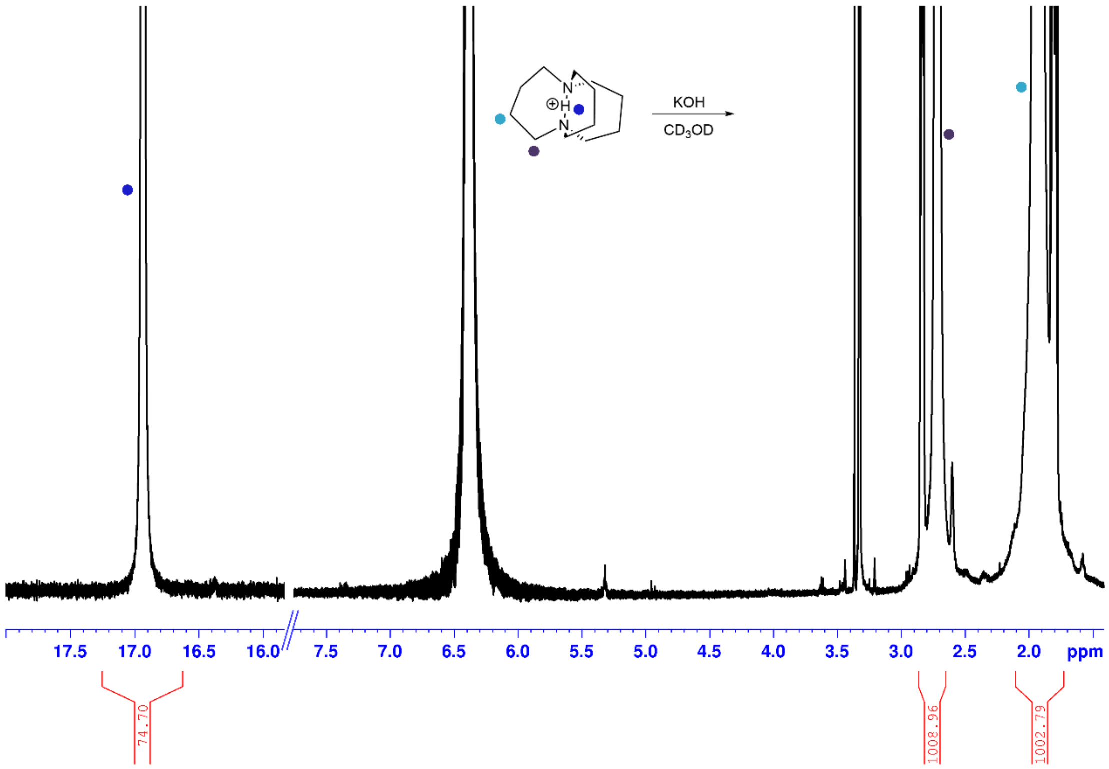

Supplementary Fig 38. Day 29 ^{1}H NMR spectra (600 MHz) of 1,6-diazabicyclo[4.4.4]tetradecan-1,6-ium [inside proton] chloride (0.03 M) in 2M KOH (CD_3OD) with sodium 3-(trimethylsilyl)-1-propanesulfonate (0.03 M) internal standard. NMR tube was heated to 80 °C for the 29 days. Overlay shows the remaining analyte peaks (colored circles), and degradation product peaks are not observed so could not be highlighted.

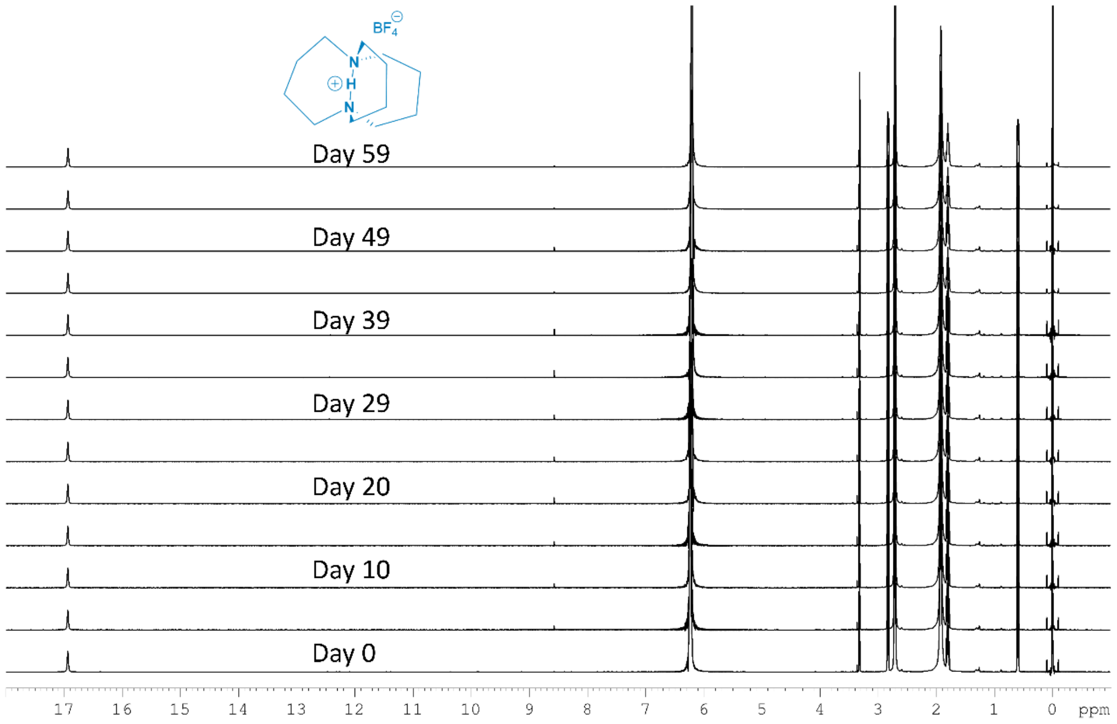

Supplementary Fig 39. Stacked ^{1}H NMR spectra (600 MHz) of 1,6-diazabicyclo[4.4.4]tetradecan-1,6-ium [inside proton] tetrafluoroborate (0.03 M) in 2M KOH (CD_3OD) with sodium 3-(trimethylsilyl)-1-propanesulfonate (0.03 M) internal standard. NMR tube was heated to 80 °C between samples, allowing to cool before the next spectrum was taken.

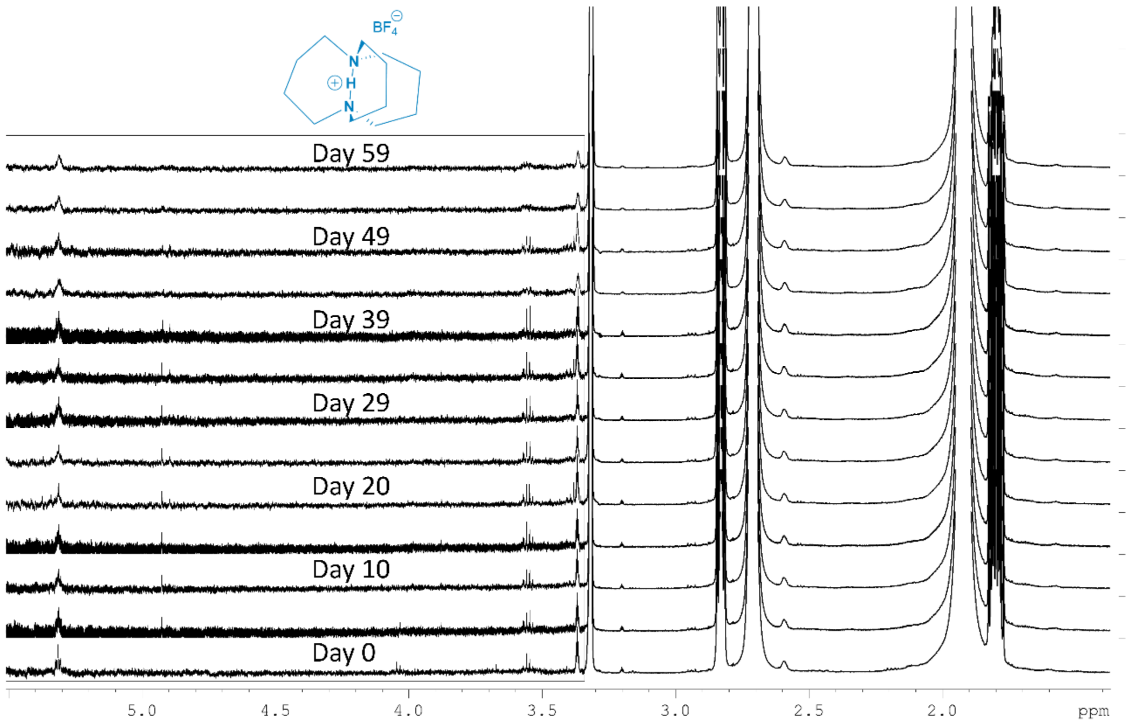

Supplementary Fig 40. Expanded, stacked ^{1}H NMR spectra (600 MHz) of 1,6-diazabicyclo[4.4.4]tetradecan-1,6-ium [inside proton] tetrafluoroborate (0.03 M) in 2M KOH (CD_3OD) with sodium 3-(trimethylsilyl)-1-propanesulfonate (0.03 M) internal standard. NMR tube was heated to 80 °C between samples, allowing to cool before the next spectrum was taken. The region in the box was increased in intensity to show the region where elimination degradation products should show up.

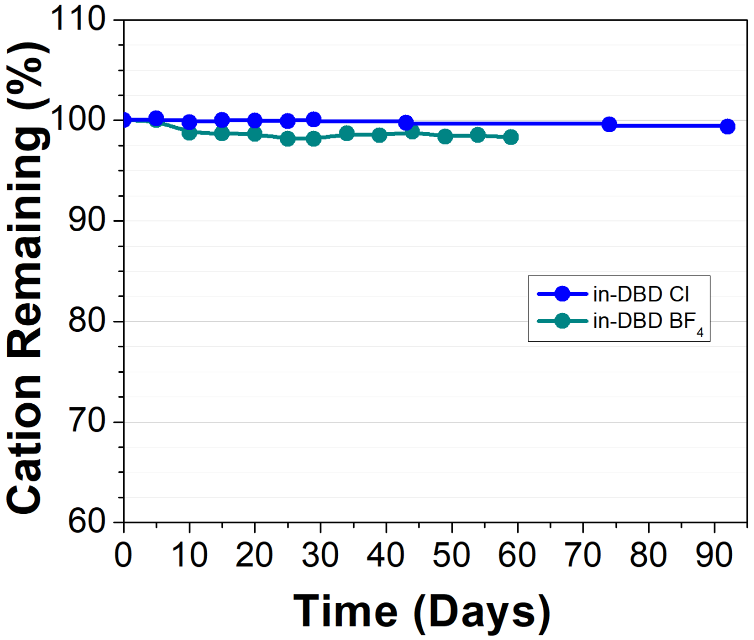

Supplementary Fig 41. Percent cation remaining of **in-DBD** in its chloride and BF_4^- ion-form.

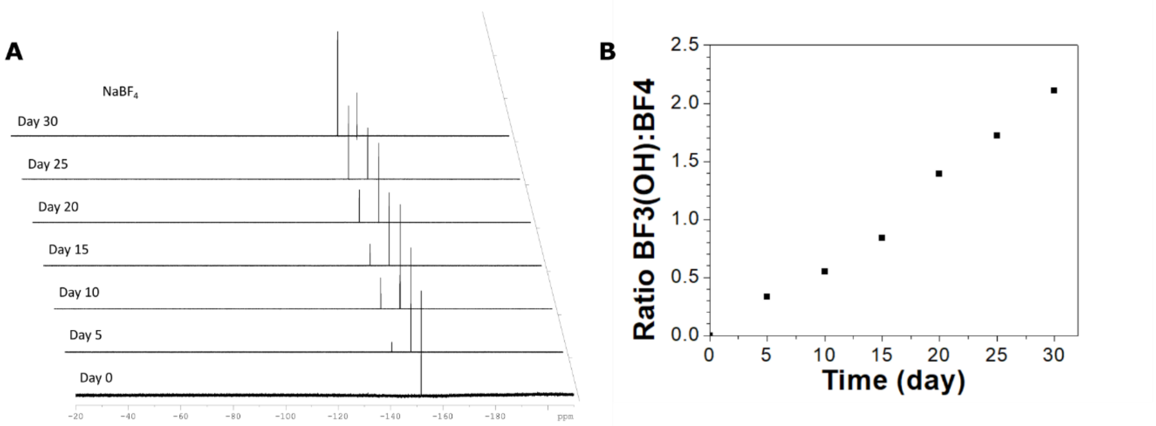

Supplementary Fig 42. (**A**) Stacked ^{19}F NMR spectra of sodium tetrafluoroborate (molarity) in 2M KOH (CD3OD) with sodium 3-(trimethylsilyl)-1-propanesulfonate (molarity) internal standard. NMR tube was heated to 80 °C between samples, allowing to cool before the next spectrum was taken. (**B**) Ratio of the integration of the $BF_3(OH)$ degradation product signal to the BF_4 signal.

To estimate a half-life of the cations in the NMR test conditions, the cation remaining vs time was plotted, and the data was fit to a linear curve with formula: $Y = 1 + b \bullet X$, where b is the slope. The half-life of the cation was calculated to be where $Y = 0.5$ (50% cation remaining) and X was calculated, in hours. The value was rounded to the nearest 10 hours. Over 30 days, a loss

of ~0.5% cation would have a half-life of roughly 100000 hours, which is the maximum lifetime this experiment can resolve.

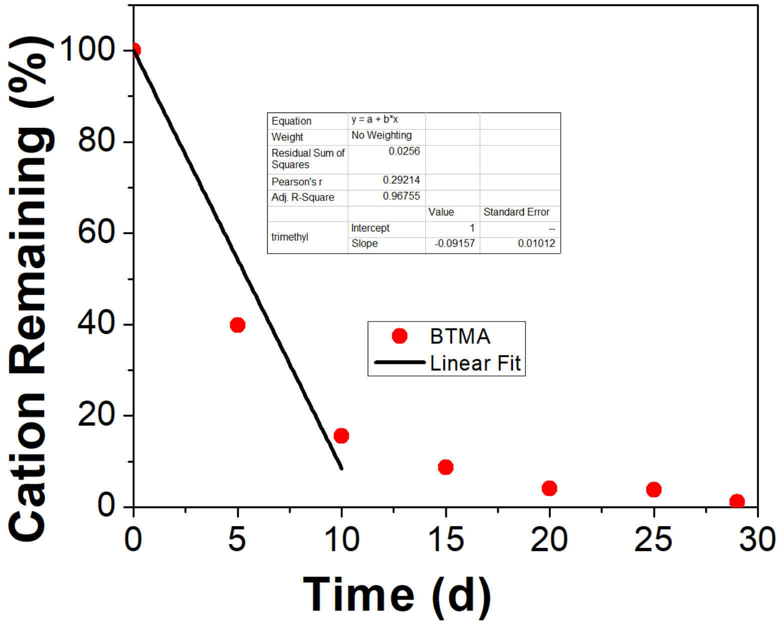

Supplementary Fig 43. NMR stability plot of cation remaining over time for benzyltrimethylammonium chloride (0.03 M) in 2M KOH (CD_3OD) relative to a sodium 3-(trimethylsilyl)-1-propanesulfonate (0.03 M) internal standard. The black line is a linear fit of the collected data, for the first 10 days.

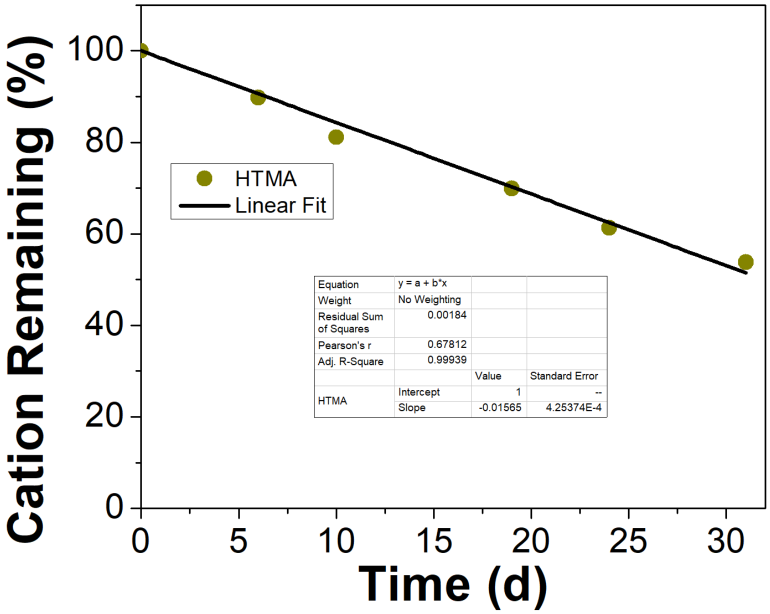

Supplementary Fig 44. NMR stability plot of cation remaining over time for hexadecyltrimethylammonium bromide (0.03 M) in 2M KOH (CD_3OD) relative to a sodium 3-(trimethylsilyl)-1-propanesulfonate (0.03 M) internal standard. The black line is a linear fit of the collected data.

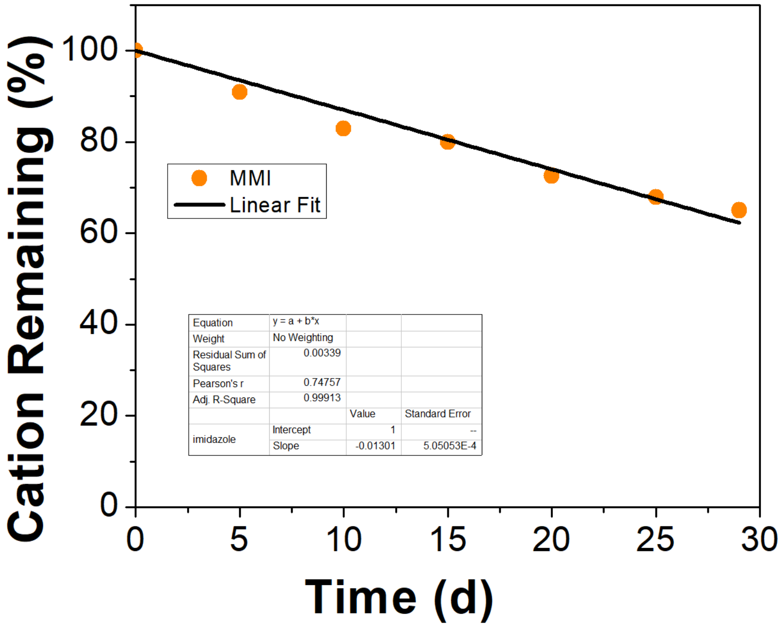

Supplementary Fig 45. NMR stability plot of cation remaining over time for 1,3-dimethyl-2-mesityl-4,5-diphenyl-1H-imidazol-3-ium iodide (0.03 M) in 2M KOH (CD_3OD) relative to a sodium 3-(trimethylsilyl)-1-propanesulfonate (0.03 M) internal standard. The black line is a linear fit of the collected data.

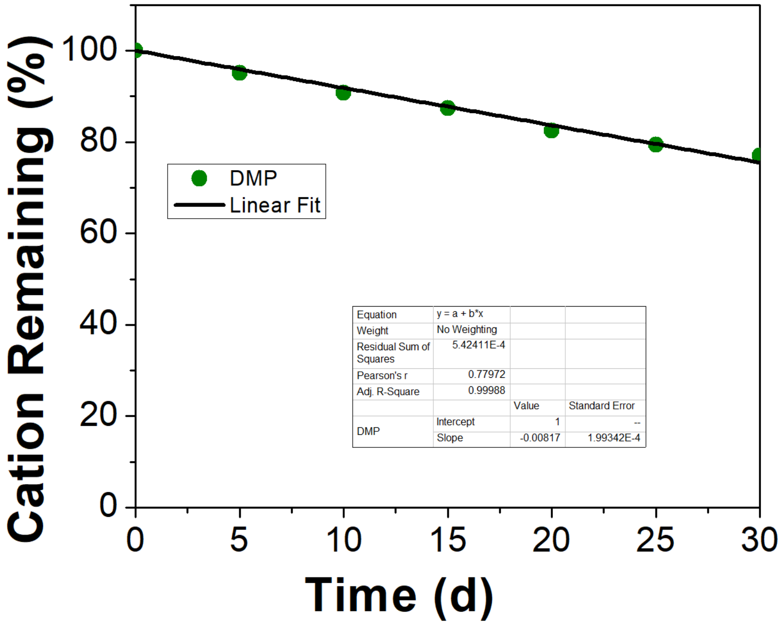

Supplementary Fig 46. NMR stability plot of cation remaining over time for 1,1-dimethylpiperidinium iodide (0.03 M) in 2M KOH (CD_3OD) relative to a sodium 3-(trimethylsilyl)-1-propanesulfonate (0.03 M) internal standard. The black line is a linear fit of the collected data.

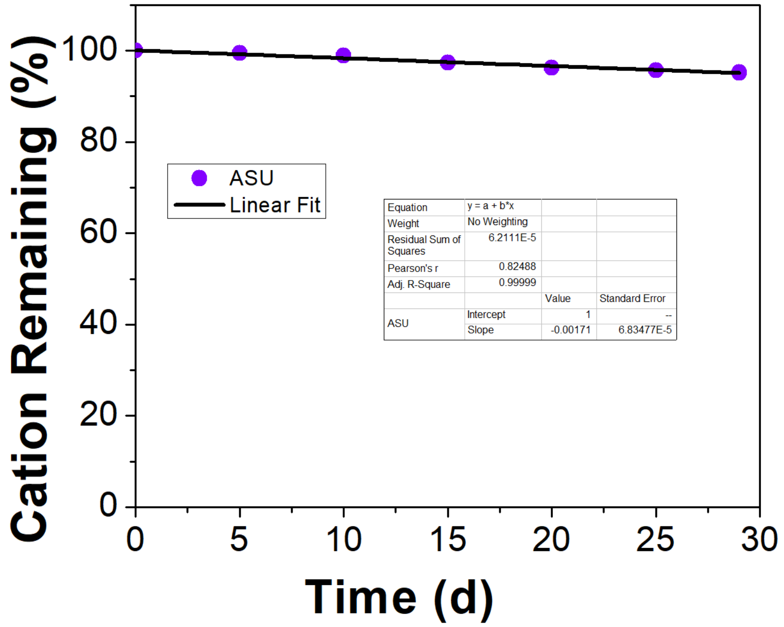

Supplementary Fig 47. NMR stability plot of cation remaining over time for 6-azaspiro[5.5]undecan-6-ium bromide (0.03 M) in 2M KOH (CD_3OD) relative to a sodium 3-(trimethylsilyl)-1-propanesulfonate (0.03 M) internal standard. The black line is a linear fit of the collected data.

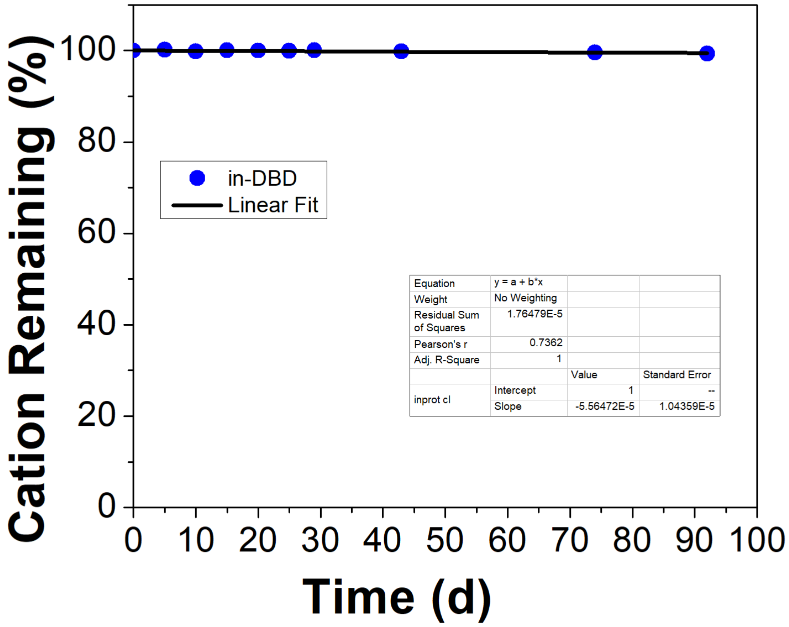

Supplementary Fig 48. NMR stability plot of cation remaining over time for 1,6-diazabicyclo[4.4.4]tetradecan-1,6-ium [inside proton] chloride (0.03 M) in 2M KOH (CD_3OD) relative to a sodium 3-(trimethylsilyl)-1-propanesulfonate (0.03 M) internal standard. The black line is a linear fit of the collected data.

<u>DVS degradation studies:</u>

DVS degradation tests were performed on the hydroxide-form of the studied ions. To ensure minimal carbonation of the ions, all manipulations were performed under a flow of argon.

Amberlyst A26-OH form ion-exchange resin was refreshed by adding new resin (as received) to a Schlenk flask, and degassed Milli-Q water was added. The mixture was sparged with argon for 1 hour, then KOH (85%) pellets were added under argon to the mixture to make ~ 1M KOH solution. The mixture was left under argon for ~ 2 hours, and regenerated 2 more times (each for 2 hours) with fresh degassed 1M KOH solution under argon, removing the old solution via syringe under argon. The resin was then washed with degassed Milli-Q water ×3 under argon, and then stored in a sealed Schlenk flask with excess degassed Milli-Q water under argon until use.

Under a flow of argon, a pasture-pipette with a small cotton pad (to hold the resin) was loaded with freshly refreshed Amberlyst A26-OH resin (1 g), and washed 3 times with degassed Milli-Q water. Under a flow of argon, 50 mg of cation in its respective halide salt form was dissolved in 1 mL of degassed Milli-Q water (or 1.5 mL of degassed 1:1 MeOH:H_2O mixture for MMI iodide and HTMA bromide). The solution was added to the resin under argon, allowing it to gravity drain into a clean glass vial purged with argon. The solution was then added back to the column 2 more times, each time collecting in a new argon-purged vial. The column was then rinsed with 0.5 mL of degassed Milli-Q water (or degassed 1:1 MeOH:H_2O), collecting in the

last vial. The final solution was topped with argon and vial closed, sealing with electrical tape (3M) until used (not stored for more than 1 hour).

The solution of organic cation-hydroxide salt in water (or 1:1 MeOH:H_2O for MMI and HTMA hydroxide) was added to the DVS crucible (Pt crucible, 0.5 mL) and quickly loaded into the DVS under an argon flow (200 SCCM). The remaining solution was then tested for pH of direct sample (all samples recorded pH of 12-13) and ionic conductivity (Omega CDH-7021) of sample diluted 8 times to ensure the majority of the anions were hydroxide anions.* The DVS sample was dried at 80 °C under an argon flow (200 SCCM total) at 55% RH (nominally 60% RH), until a stable mass was achieved for at least 1 hour. A step-program was used to alter the RH, maintaining the flow rate (200 SCCM) and oven temperature (80 °C), as shown below. Intermittent re-humidification of cations to a point with no degradation (stable reference RH, 55% RH) was performed at specific intervals; this is used as a conformation of degradation, visualized as lower water uptake under the same conditions due to loss of hygroscopic ionic species. 30% RH to 10% RH are considered critical conditions, so the RH was returned to the stable reference (55%) RH before and after these conditions to observe degradation-related decreases in hygroscopicity.

^{1}H NMR were collected on the remaining solution after exchange, to ensure no degradation on the column was observed, and the ^{1}H NMR was collected on the mass remaining after the DVS run was finished. The remaining mass is a combination of non-volatile degradation products, carbonated form salt, and un-exchanged halide form salt.

*A conductivity of 2.4 mS/cm is for pure hydroxide conductivity at ~ 0.012 M solution, so conductivities above this were taken to be a majority OH- form organic salts; 0.53 mS/cm is the pure HCO_3- conductivity so a majority carbonated sample could not show these conductivities seen. For example, after hydroxide exchange of **in-DBD**, a measured conductivity of 3.0 mS/cm at ~ 0.012M was recorded, showing the relative contributions of the cations and anions require mostly hydroxide as the mobile anion.

To determine degradation of the samples, the data was normalized to the average mass at the reference RH (55%) before degradation. The last 30 minutes of each RH value were taken and the data was fit to a linear slope ($y = mx+b$). The onset of degradation was determined as the RH where more than 0.02% mass loss was observed (from the fitted data) over the 30 minutes. Substantial degradation was defined by an RH where more than 0.1% mass loss was observed (from the fitted data) over the 30 minutes.

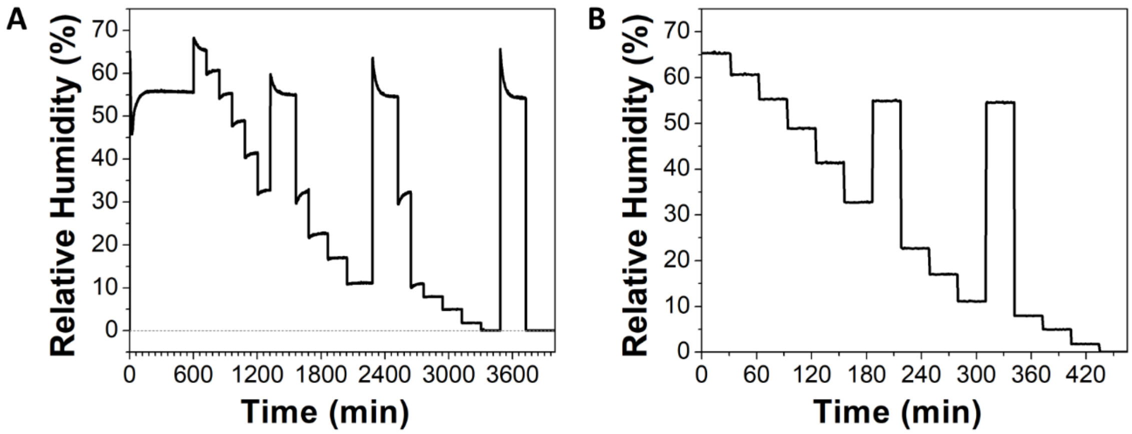

Supplementary Fig 49. DVS RH procedure for sweeping RH scans. (A) Raw relative humidity vs time of DVS studies and (B) the last 30 minutes of each new (or reference) RH. The temperature was maintained at 80 °C.

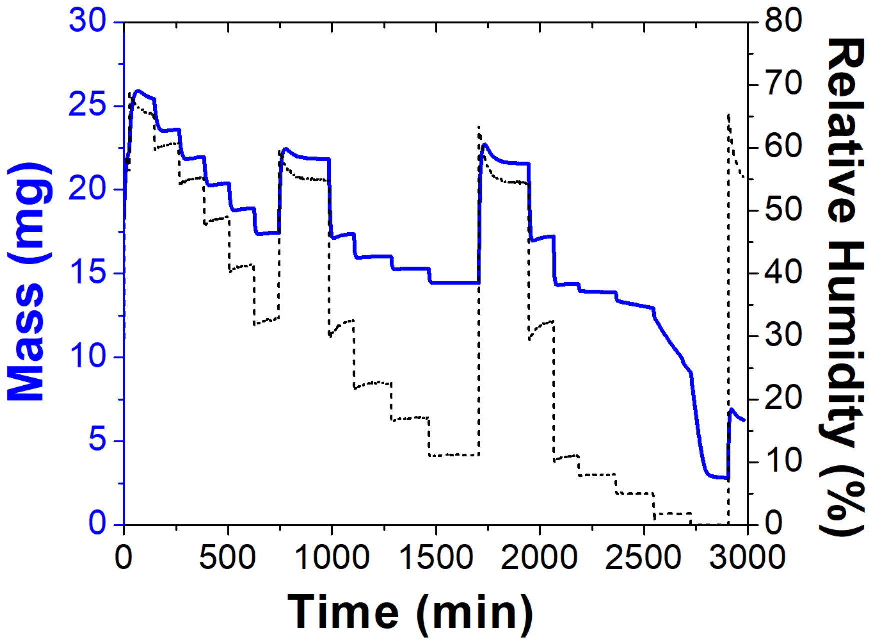

Supplementary Fig 50. Dynamic Vapor Sorption analysis of **in-DBD** hydroxide at decreasing relative humidity levels. The temperature was maintained at 80 °C throughout the experiment. The colored line is for the left, colored axis and the black dotted line is for the right, black axis.

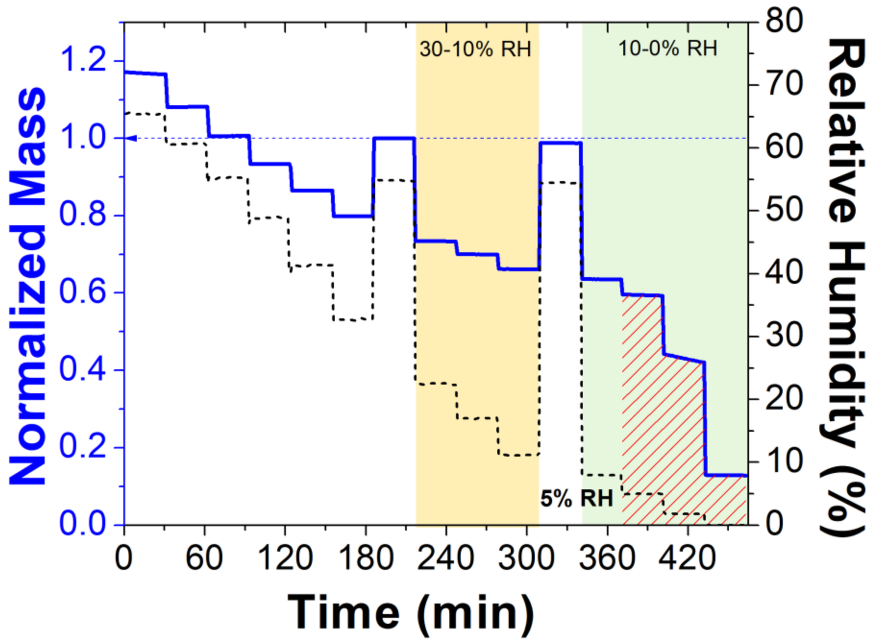

Supplementary Fig 51. Dynamic Vapor Sorption analysis of **in-DBD** hydroxide at decreasing relative humidity levels. The mass was normalized to the average mass after drying at 55% RH. Only the last 30 minutes of each step is displayed, which is taken as the time after equilibration of the RH. The temperature was maintained at 80 °C throughout the experiment. The colored line is for the left, colored axis and the black dotted line is for the right, black axis. 30% RH to 10% RH are considered critical conditions, so the RH was returned to the stable reference (55%) RH before and after these conditions to observe degradation-related decreases in hygroscopicity. The colored, dotted arrow represents the mass at the reference RH prior to degradation; yellow and green regions represent 30-10 and 10-0% RH conditions, respectively. Red shading represents the RH region where degradation occurs for each cation and the number beside is the RH where substantial degradation (>0.2% mass loss/h) is first observed upon lowering the RH.

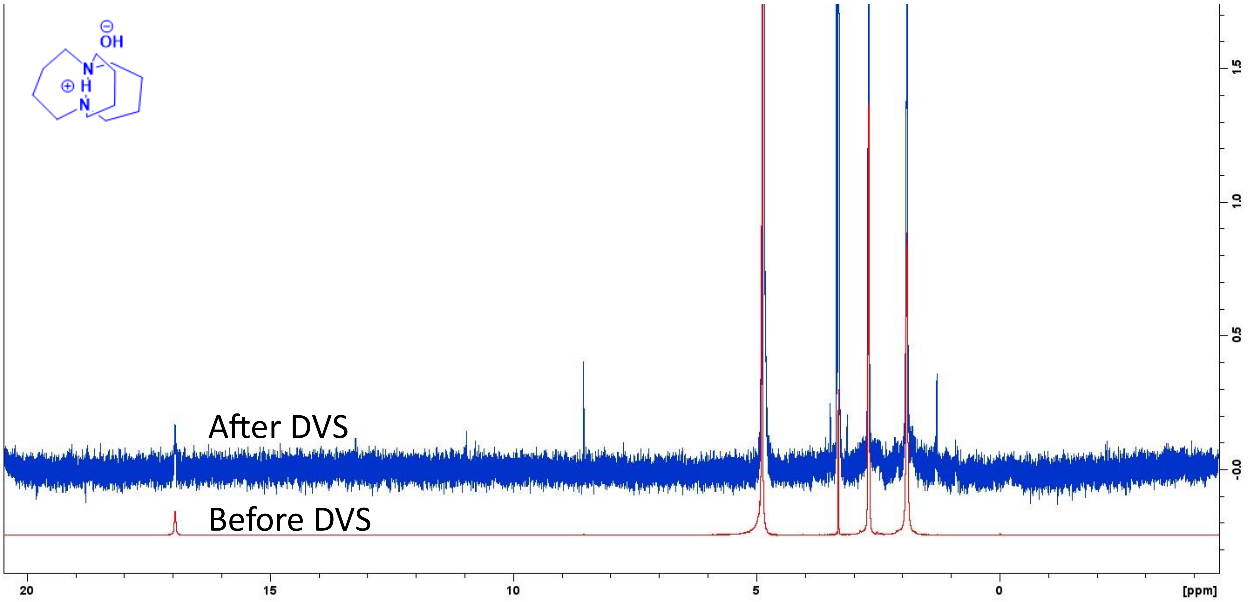

Supplementary Fig 52. Stacked ^{1}H NMR spectra (500 MHz) of **in-DBD** hydroxide before (red) and after (blue) the DVS experiment. The "before DVS" sample is of the hydroxide-exchanged cation, and allowed to carbonate in open air for several days before removing solvent to obtain the NMR spectra.

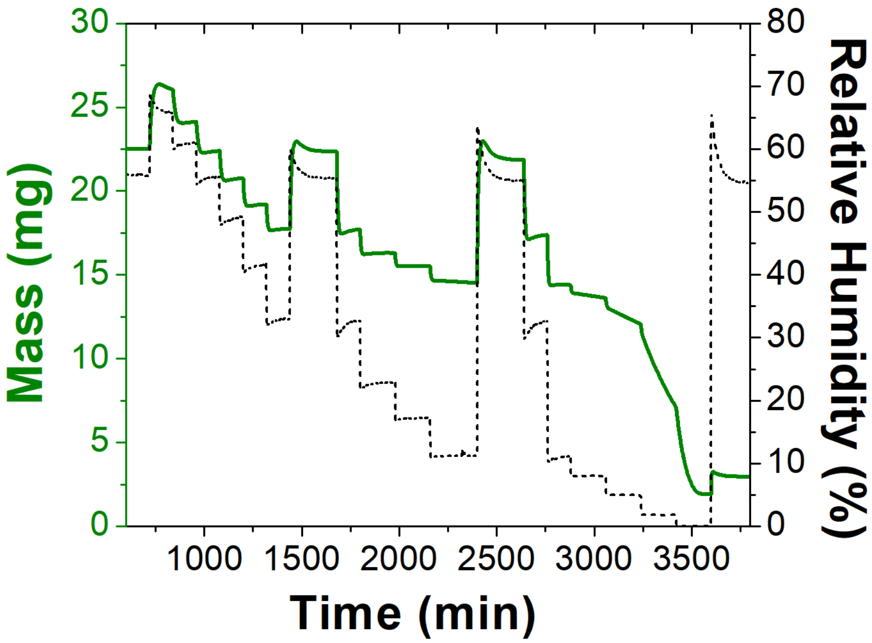

Supplementary Fig 53. Dynamic Vapor Sorption analysis of DMP hydroxide at decreasing relative humidity levels. The temperature was maintained at 80 °C throughout the experiment. The colored line is for the left, colored axis and the black dotted line is for the right, black axis.

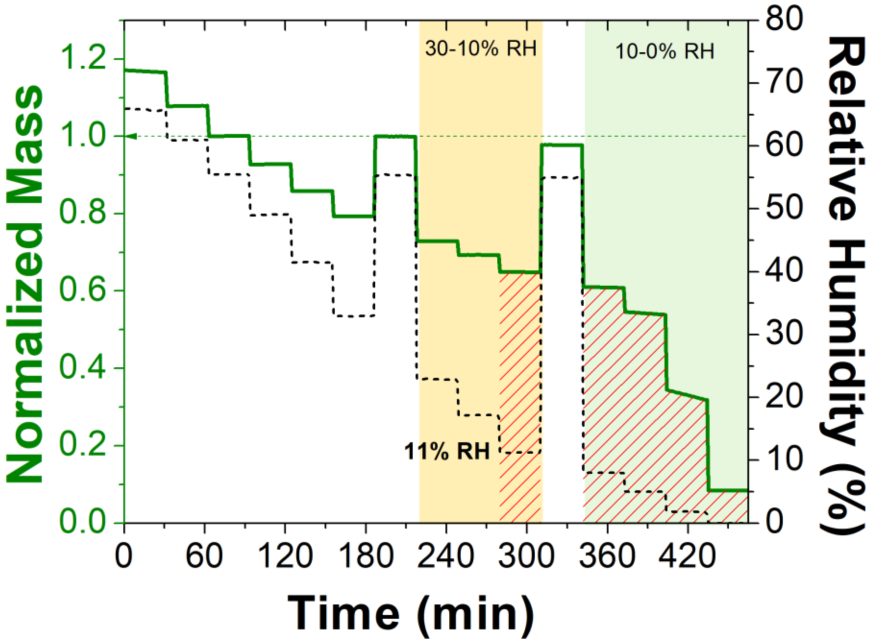

Supplementary Fig 54. Dynamic Vapor Sorption analysis of DMP hydroxide at decreasing relative humidity levels. The mass was normalized to the average mass after drying at 55% RH. Only the last 30 minutes of each step is displayed, which is taken as the time after equilibration of the RH. The temperature was maintained at 80 °C throughout the experiment. The colored line is for the left, colored axis and the black dotted line is for the right, black axis. 30% RH to 10% RH are considered critical conditions, so the RH was returned to the stable reference (55%) RH before and after these conditions to observe degradation-related decreases in hygroscopicity. The colored, dotted arrow represents the mass at the reference RH prior to degradation; yellow and green regions represent 30-10 and 10-0% RH conditions, respectively. Red shading represents the RH region where degradation occurs for each cation and the number beside is the RH where substantial degradation (>0.2% mass loss/h) is first observed upon lowering the RH.

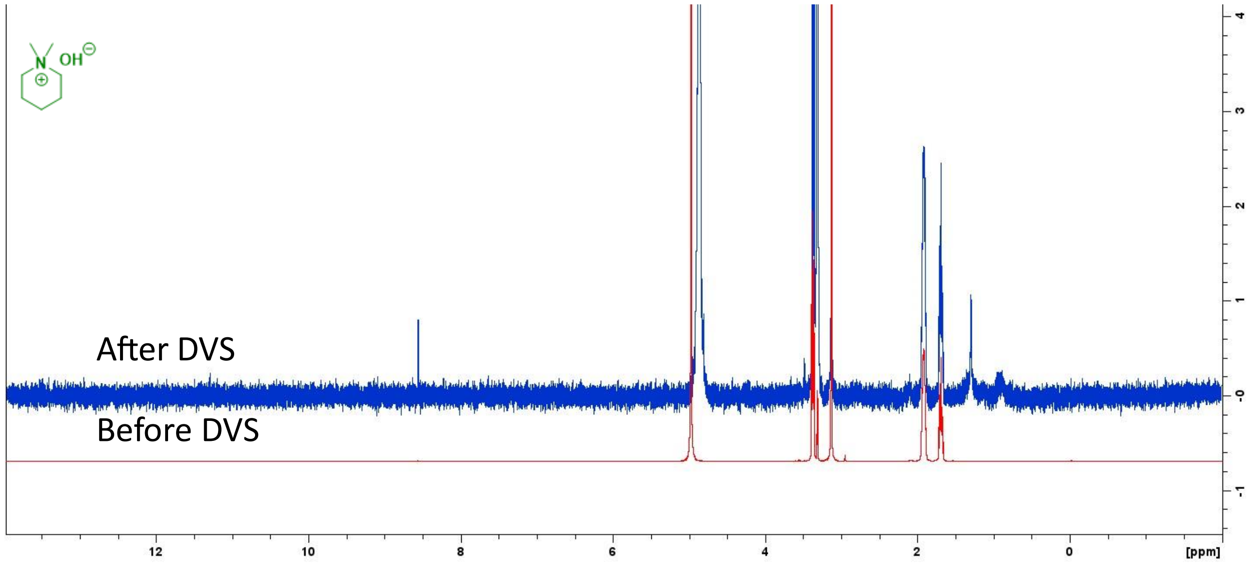

Supplementary Fig 55. Stacked ^{1}H NMR spectra (500 MHz) of DMP hydroxide before (red) and after (blue) the DVS experiment. The "before DVS" sample is of the hydroxide-exchanged cation, and allowed to carbonate in open air for several days before removing solvent to obtain the NMR spectra.

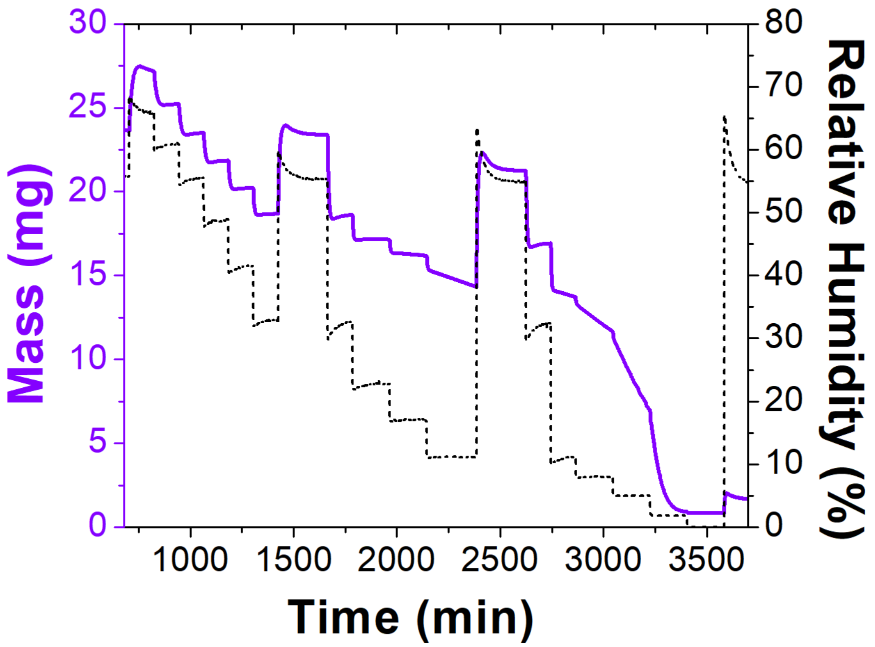

Supplementary Fig 56. Dynamic Vapor Sorption analysis of ASU hydroxide at decreasing relative humidity levels. The temperature was maintained at 80 °C throughout the experiment. The colored line is for the left, colored axis and the black dotted line is for the right, black axis.

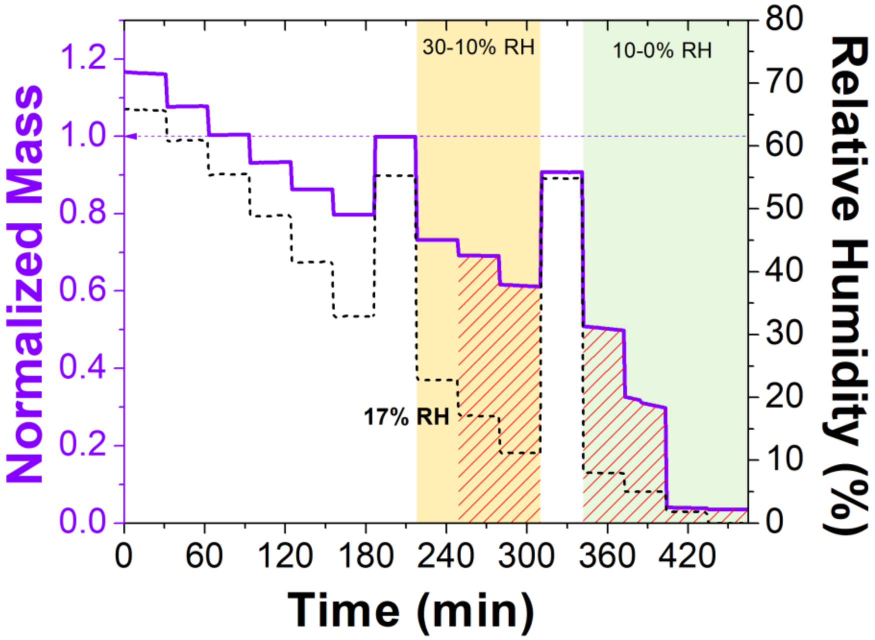

Supplementary Fig 57. Dynamic Vapor Sorption analysis of ASU hydroxide at decreasing relative humidity levels. The mass was normalized to the average mass after drying at 55% RH. Only the last 30 minutes of each step is displayed, which is taken as the time after equilibration of the RH. The temperature was maintained at 80 °C throughout the experiment. The colored line is for the left, colored axis and the black dotted line is for the right, black axis. 30% RH to 10% RH are considered critical conditions, so the RH was returned to the stable reference (55%) RH before and after these conditions to observe degradation-related decreases in hygroscopicity. The colored, dotted arrow represents the mass at the reference RH prior to degradation; yellow and green regions represent 30-10 and 10-0% RH conditions, respectively. Red shading represents the RH region where degradation occurs for each cation and the number beside is the RH where substantial degradation (>0.2% mass loss/h) is first observed upon lowering the RH.

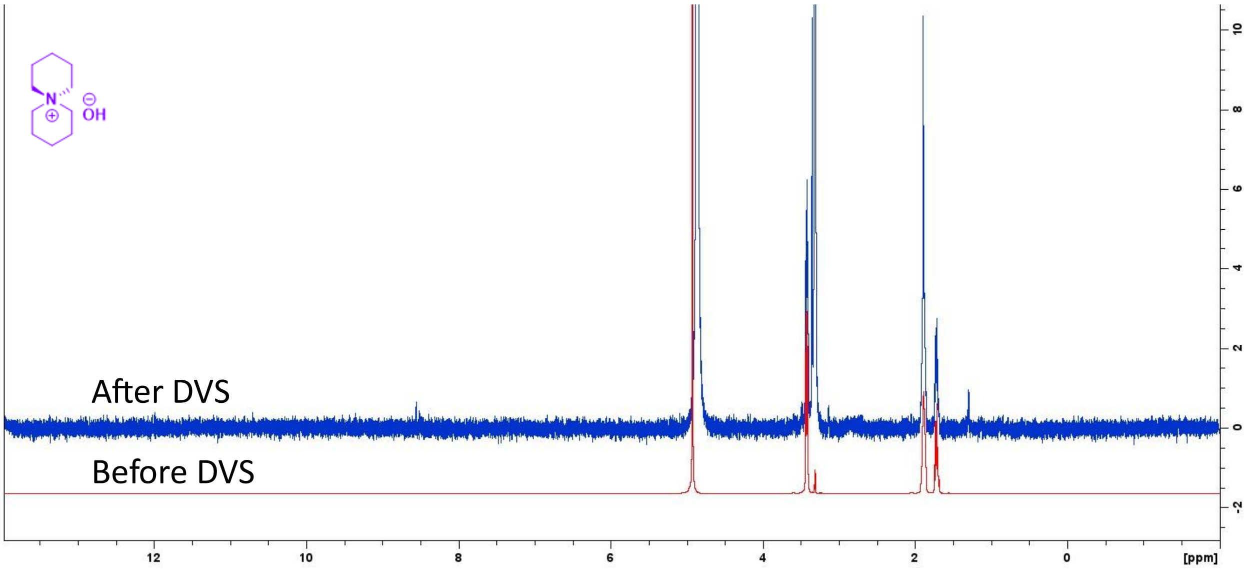

Supplementary Fig 58. Stacked ^{1}H NMR spectra (500 MHz) of ASU hydroxide before (red) and after (blue) the DVS experiment. The "before DVS" sample is of the hydroxide-exchanged cation, and allowed to carbonate in open air for several days before removing solvent to obtain the NMR spectra.

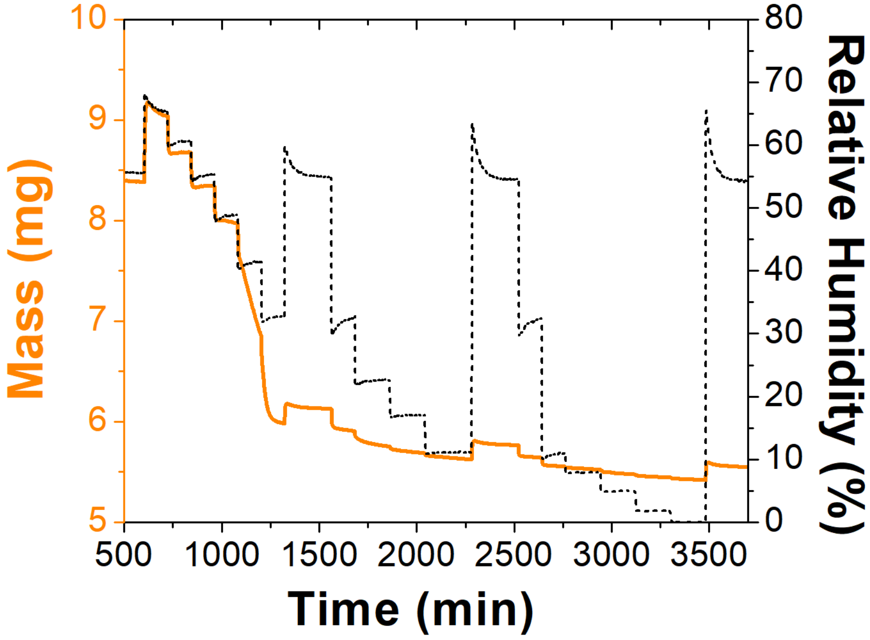

Supplementary Fig 59. Dynamic Vapor Sorption analysis of MMI hydroxide at decreasing relative humidity levels. The temperature was maintained at 80 °C throughout the experiment. The colored line is for the left, colored axis and the black dotted line is for the right, black axis.

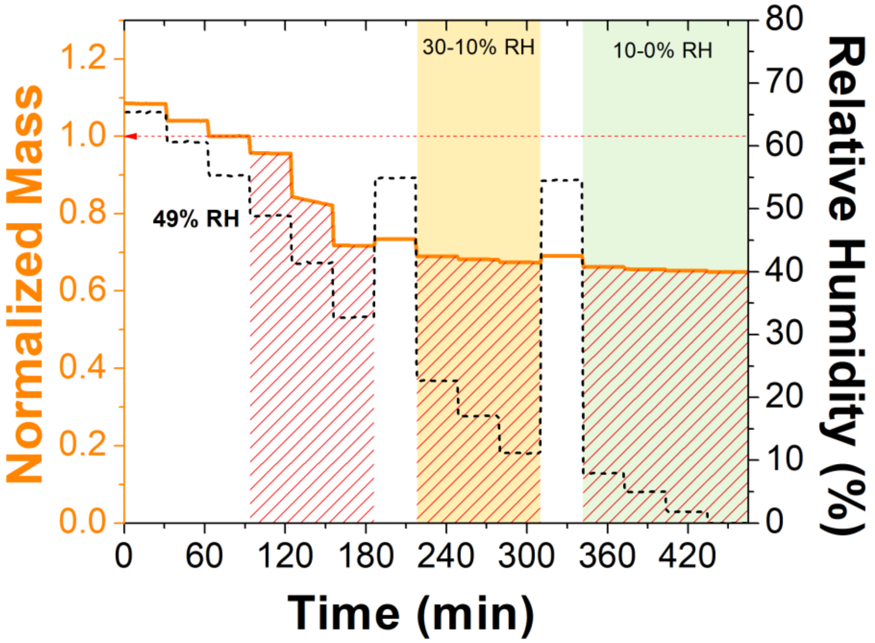

Supplementary Fig 60. Dynamic Vapor Sorption analysis of MMI hydroxide at decreasing relative humidity levels. The mass was normalized to the average mass after drying at 55% RH. Only the last 30 minutes of each step is displayed, which is taken as the time after equilibration of the RH. The temperature was maintained at 80 °C throughout the experiment. The colored line is for the left, colored axis and the black dotted line is for the right, black axis. 30% RH to 10% RH are considered critical conditions, so the RH was returned to the stable reference (55%) RH before and after these conditions to observe degradation-related decreases in hygroscopicity. The colored, dotted arrow represents the mass at the reference RH prior to degradation; yellow and green regions represent 30-10 and 10-0% RH conditions, respectively. Red shading represents the RH region where degradation occurs for each cation and the number beside is the RH where substantial degradation (>0.2% mass loss/h) is first observed upon lowering the RH.

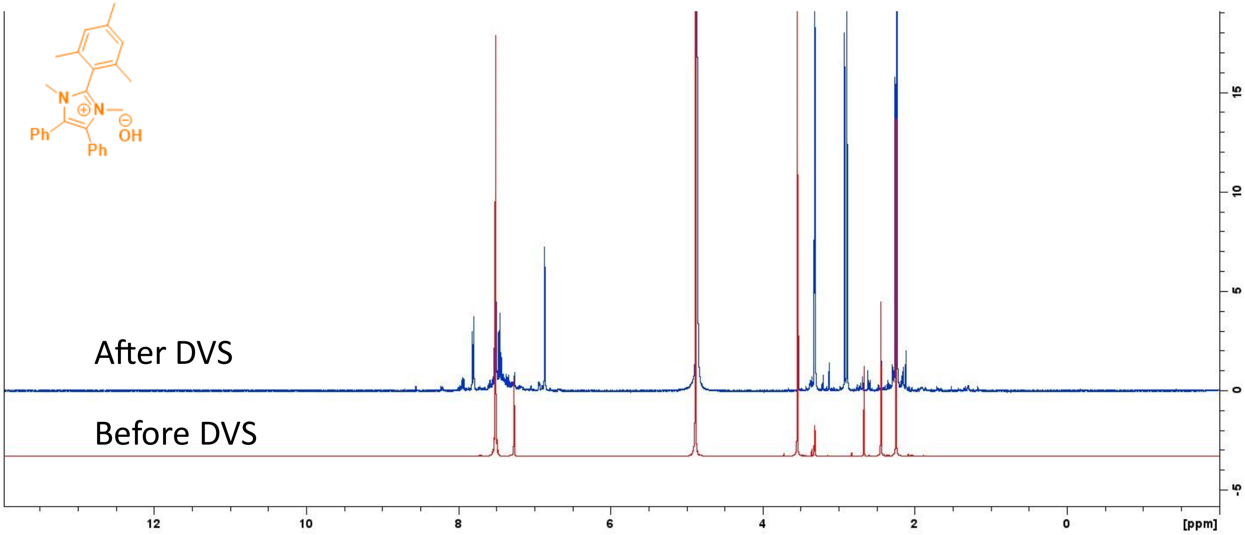

Supplementary Fig 61. Stacked ^{1}H NMR spectra (500 MHz) of MMI hydroxide before (red) and after (blue) the DVS experiment. The "before DVS" sample is of the hydroxide-exchanged cation, and allowed to carbonate in open air for several days before removing solvent to obtain the NMR spectra.

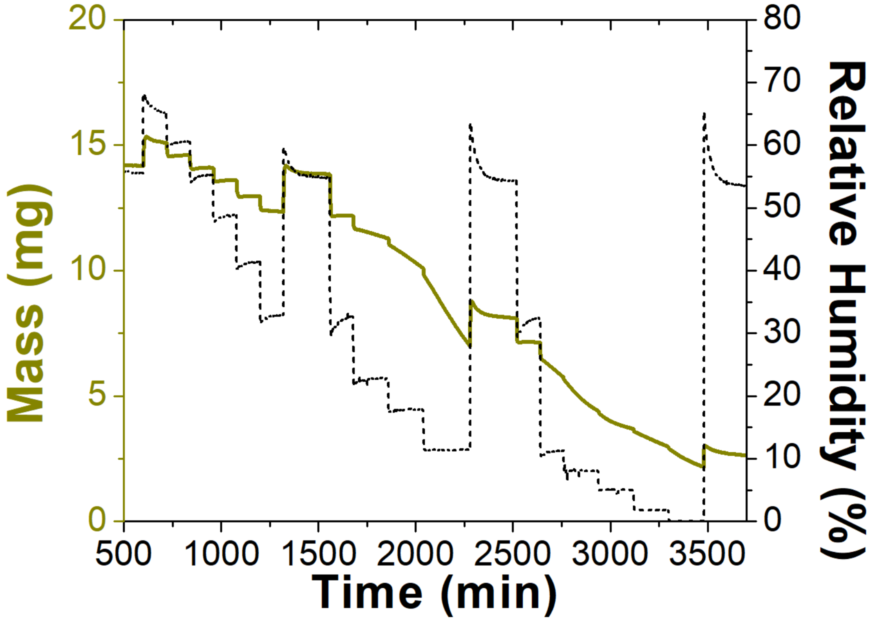

Supplementary Fig 62. Dynamic Vapor Sorption analysis of HTMA hydroxide at decreasing relative humidity levels. The temperature was maintained at 80 °C throughout the experiment. The colored line is for the left, colored axis and the black dotted line is for the right, black axis.

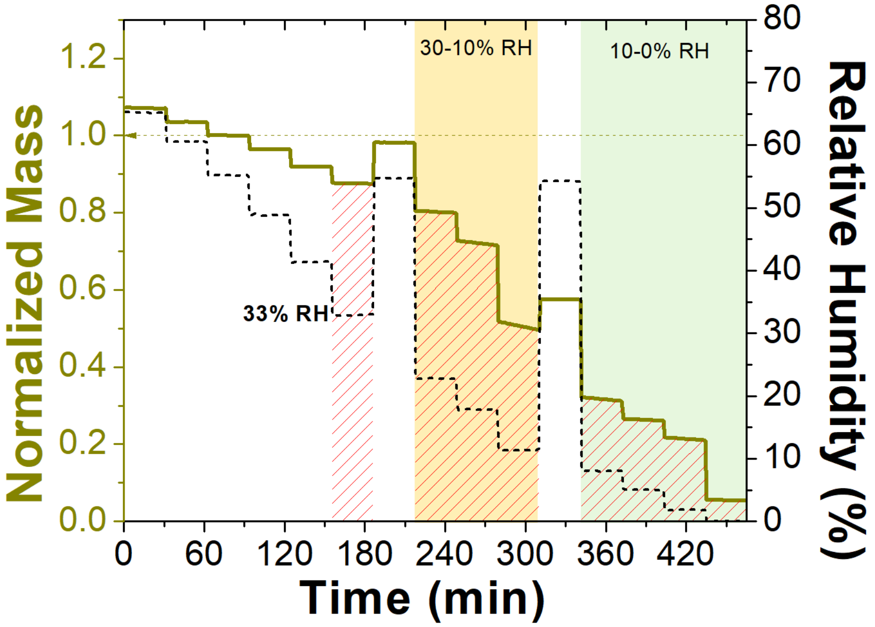

Supplementary Fig 63. Dynamic Vapor Sorption analysis of HTMA hydroxide at decreasing relative humidity levels. The mass was normalized to the average mass after drying at 55% RH. Only the last 30 minutes of each step is displayed, which is taken as the time after equilibration of the RH. The temperature was maintained at 80 °C throughout the experiment. The colored line is for the left, colored axis and the black dotted line is for the right, black axis. 30% RH to 10% RH are considered critical conditions, so the RH was returned to the stable reference (55%) RH before and after these conditions to observe degradation-related decreases in hygroscopicity. The colored, dotted arrow represents the mass at the reference RH prior to degradation; yellow and green regions represent 30-10 and 10-0% RH conditions, respectively. Red shading represents the RH region where degradation occurs for each cation and the number beside is the RH where substantial degradation (>0.2% mass loss/h) is first observed upon lowering the RH.

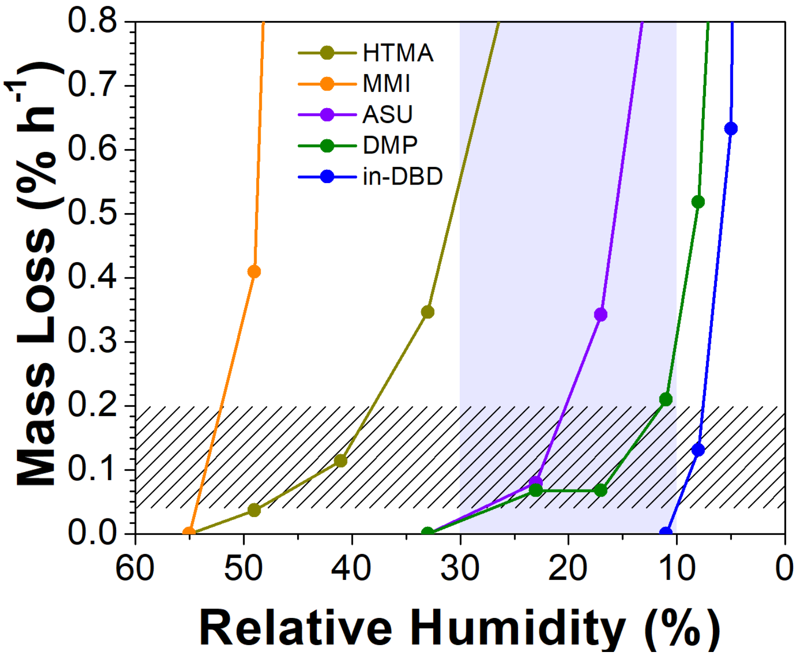

Supplementary Fig 64. Mass loss as a function of RH for the respective cation-hydroxide salts at 80 °C, measured from the slope of the last 30 minutes of each RH step. Mass was normalized to the stabilized mass at 55% RH. The hashed region is between minor (0.04% h^{-1}) and major (0.2% h^{-1}) mass loss. The pale blue backdrop shows 30-10 % RH.

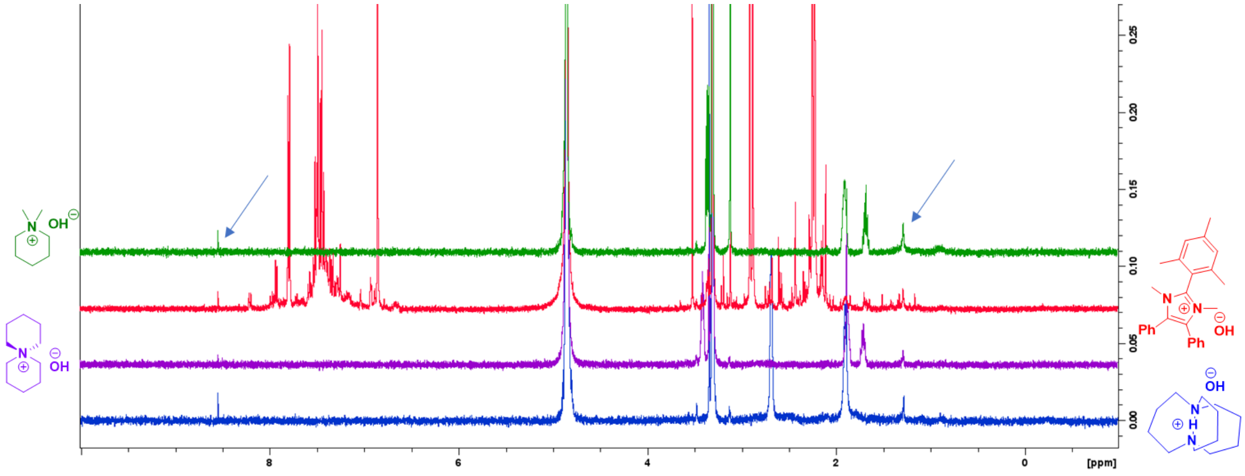

Supplementary Fig 65. Stacked ^1H NMR spectra (500 MHz) of the four cations hydroxides, taken after their respective DVS experiment and allowed to carbonate. Arrows point to common impurity/artifact arising from the experiment, not from degradation products.

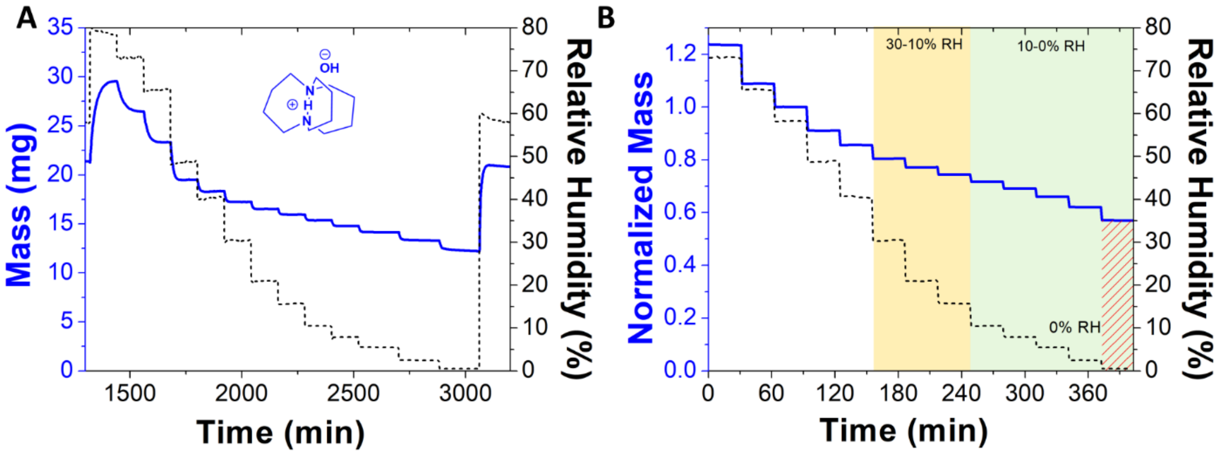

Supplementary Fig 66. (**A**) Dynamic Vapor Sorption analysis of **in-DBD** hydroxide at decreasing relative humidity levels at 60 °C. (**B**) The last 30 minutes of each RH step plotted. The yellow and green regions represent the 30-10 and 10-0% RH conditions, respectively. The red shading represents the RHs where degradation occurs, and the number beside is the first RH that substantial degradation was observed. The colored line is for the left, colored axis and the black dotted line is for the right, black axis.

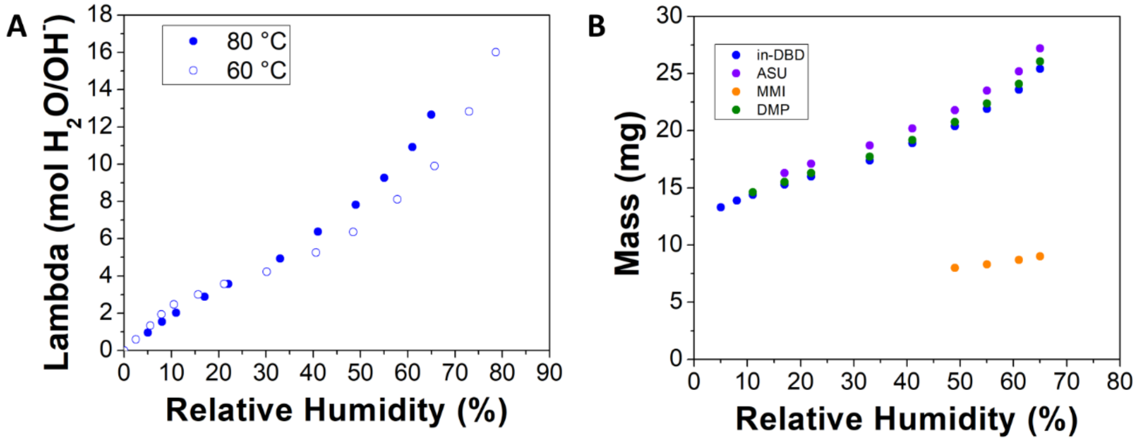

Supplementary Fig 67. (**A**) Humidity isotherms of **in-DBD** hydroxide at 60 °C and 80 °C, with estimations of hydration number from the slope to 0% RH. (**B**) Humidity isotherms of the four cation-hydroxides studied with the raw mass vs relative humidity (due to insufficient stability to estimate mass at 0% RH for all ions except **in-DBD**).

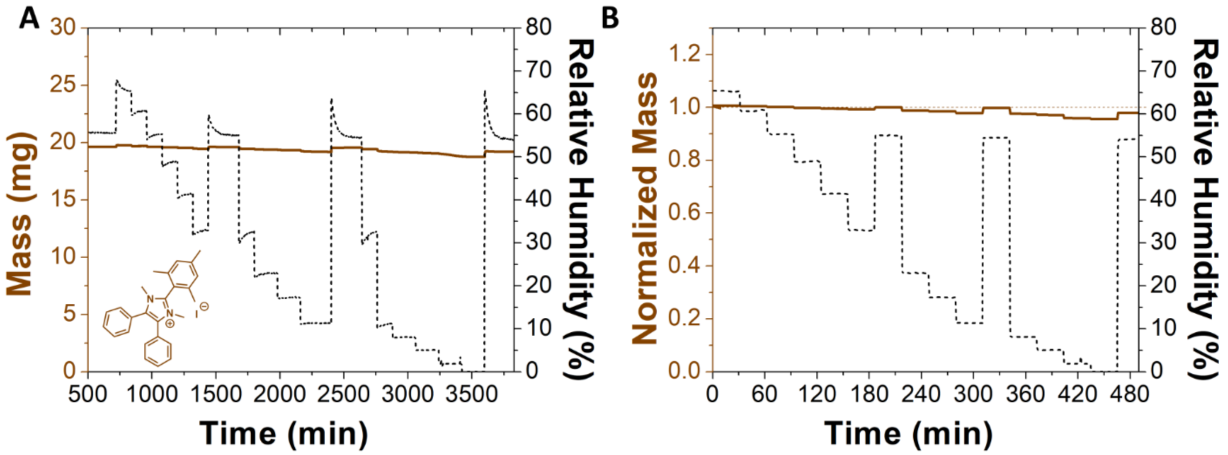

Supplementary Fig 68. (**A**) Dynamic Vapor Sorption control analysis of MMI iodide at decreasing relative humidity levels at 80 °C. (**B**) The last 30 minutes of each RH step plotted. The colored line is for the left, colored axis and the black dotted line is for the right, black axis.

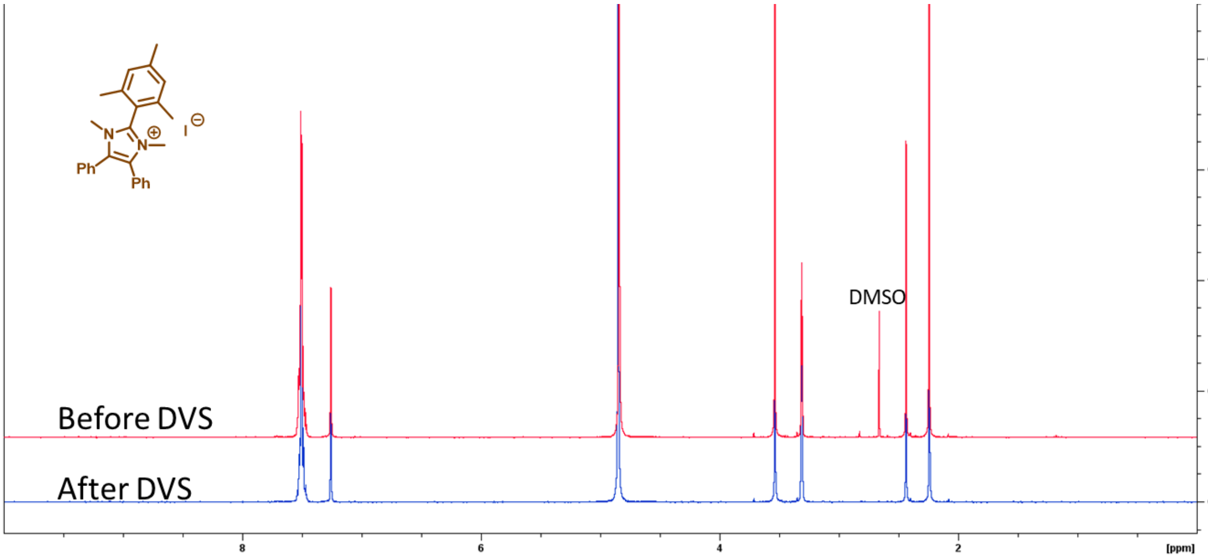

Supplementary Fig 69. Stacked ^{1}H NMR spectra (500 MHz) of MMI iodide before (red) and after (blue) the DVS experiment. *the small amount of residual DMSO was removed by the gas flow of the experiment, and is represented in the small amount of mass loss.

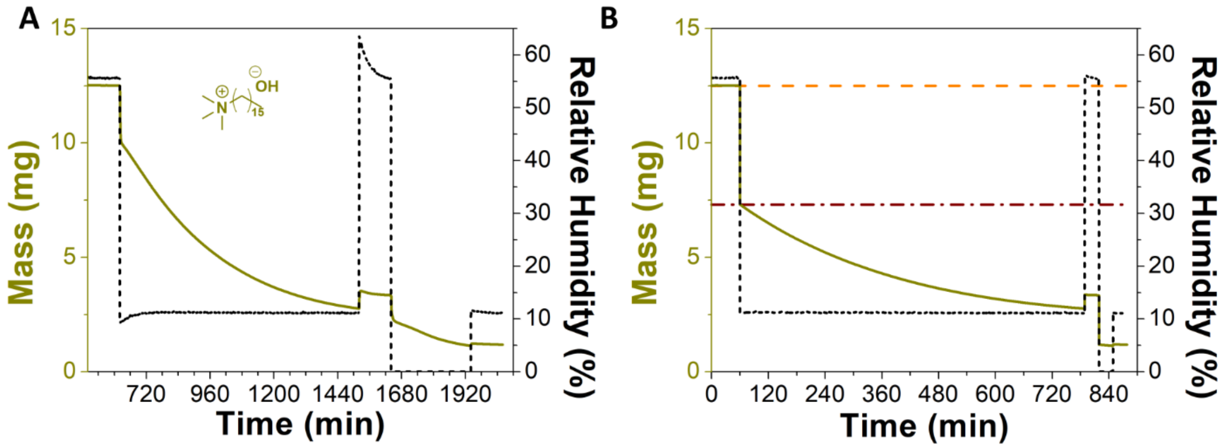

Supplementary Fig 70. DVS mass traces of HTMA hydroxide vs time at 80 °C. (A) experimental mass and RH trace over the course of the experiment, and (B) mass and RH traces of the data after the RH was stabilized for each measurement. The orange dotted line represents the reference (stable) 55% RH before degradation, and the burgundy dashed-dot line represents the initial mass at the test 11% RH. The colored line is for the left, colored axis and the black dotted line is for the right, black axis.

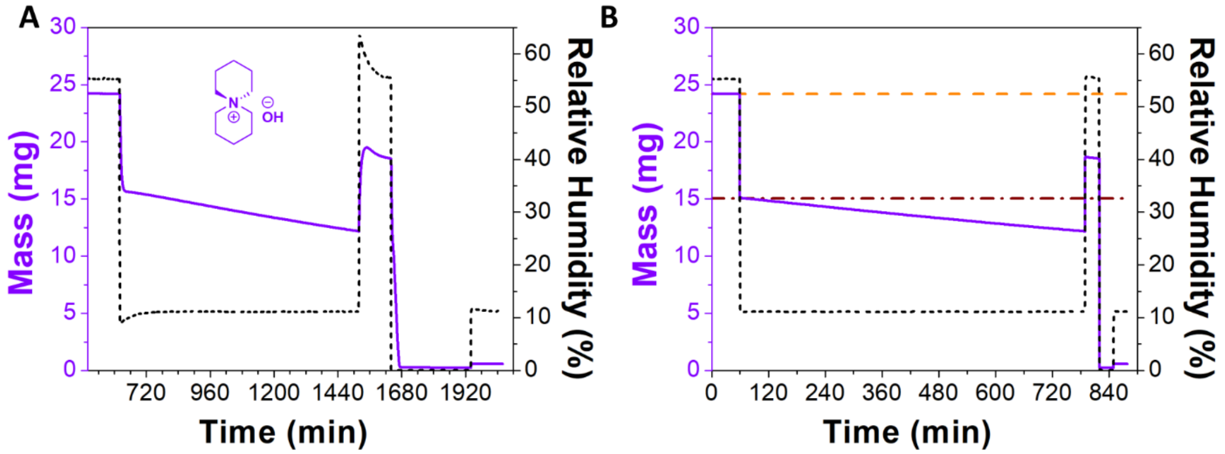

Supplementary Fig 71. DVS mass traces of ASU hydroxide vs time at 80 °C. (A) experimental mass and RH trace over the course of the experiment, and (B) mass and RH traces of the data after the RH was stabilized for each measurement. The orange dotted line represents the reference (stable) 55% RH before degradation, and the burgundy dashed-dot line represents the initial mass at the test 11% RH. The colored line is for the left, colored axis and the black dotted line is for the right, black axis.

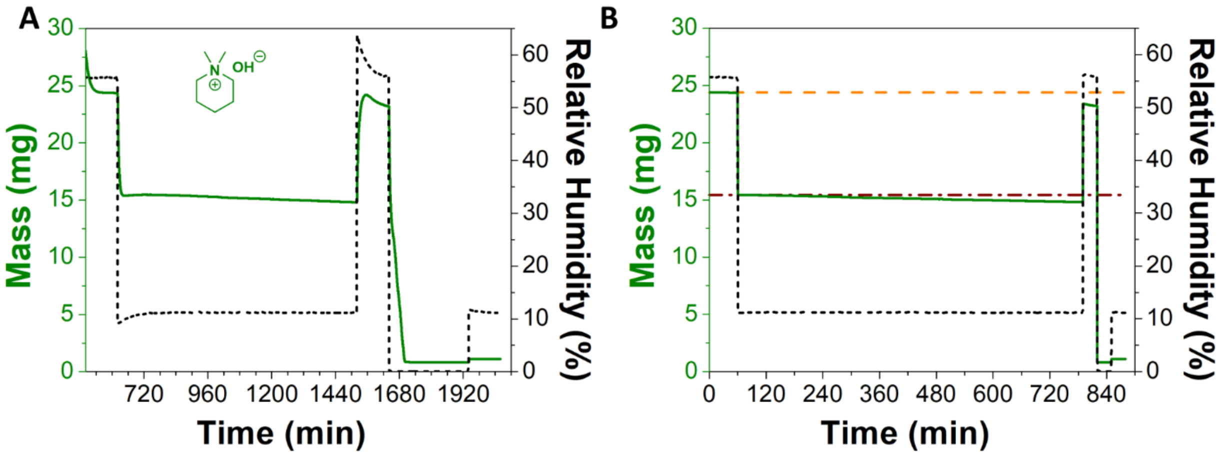

Supplementary Fig 72. DVS mass traces of DMP hydroxide vs time at 80 °C. (A) experimental mass and RH trace over the course of the experiment, and (B) mass and RH traces of the data after the RH was stabilized for each measurement. The orange dotted line represents the reference (stable) 55% RH before degradation, and the burgundy dashed-dot line represents the initial mass at the test 11% RH. The colored line is for the left, colored axis and the black dotted line is for the right, black axis.

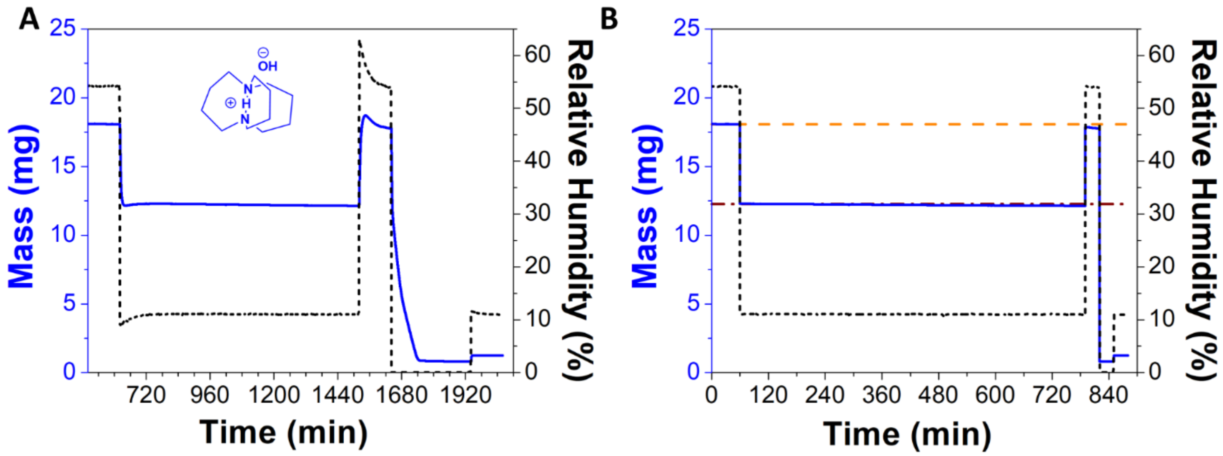

Supplementary Fig 73. DVS mass traces of in-DBD hydroxide vs time at 80 °C. (A) experimental mass and RH trace over the course of the experiment, and (B) mass and RH traces of the data after the RH was stabilized for each measurement. The orange dotted line represents the reference (stable) 55% RH before degradation, and the burgundy dashed-dot line represents the initial mass at the test 11% RH. The colored line is for the left, colored axis and the black dotted line is for the right, black axis.

To calculate the DVS half-life of the cations at 11% RH, the time after RH stabilization (time = 800 min) was set to time = 0. The mass after decomposition at RH = 0 and re-hydration to RH = 11% was considered the non-degradable mass and subtracted from the mass at each point in the degradation curve (at RH = 11 between t = 800 min and t = 1520 min). The mass at t = 0 minus the non-degradable mass, is considered the degradable mass*. The degradation curves were fit to a linear curve with formula: $Y = m_d + b \cdot X$, where b is the slope and m_d is the

degradable mass. The half-life of the cation was calculated to be where $Y = 0.5$ (50% cation remaining) and X was calculated, in hours. The value was rounded to the nearest 10 hours.

* Since the four cations studied (HTMA, ASU, DMP, in-DBD) all had 100% volatile degradation products, this assumption can be made and we caution others from making this assumption if the cations studied leave non-volatile degradation products. This is the reason MMI was not included in the calculation of a DVS half-life.

To capture the degradation product of in-DBD, the out-gas of the DVS degradation experiment was bubbled through a solution of 5% v/v acetic acid in water. The resulting solution was dried under reduced pressure and the residue was collected and characterized by NMR and MS.

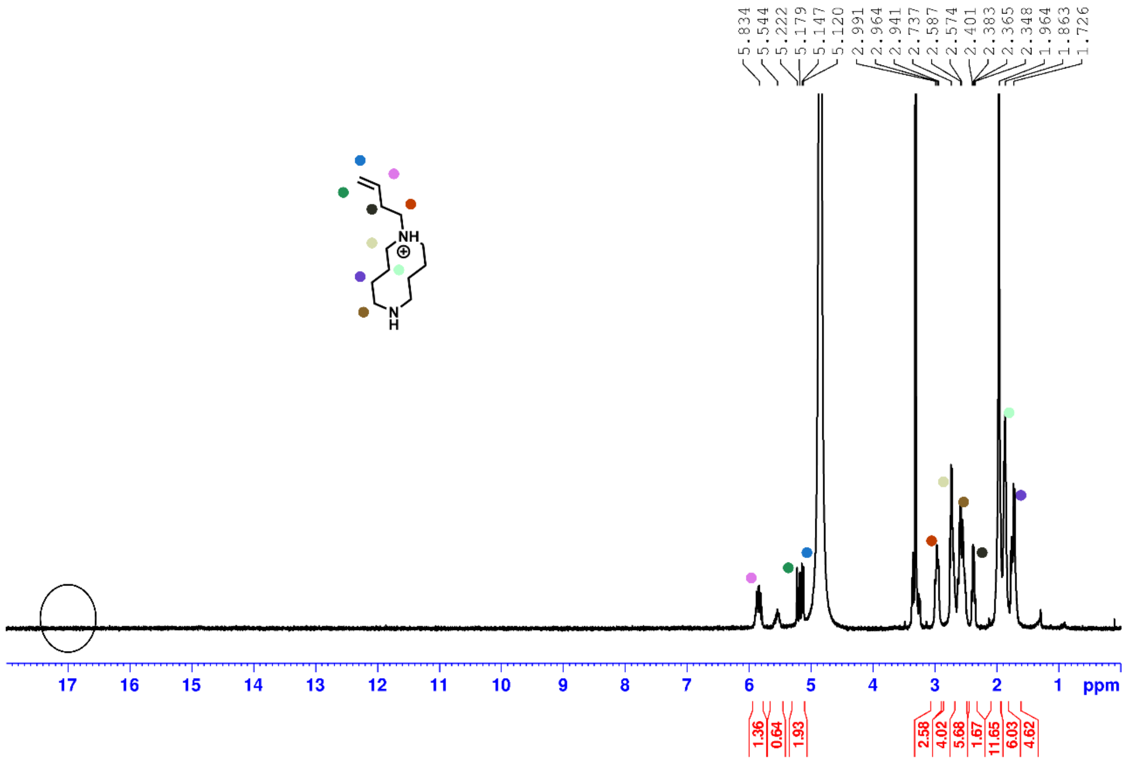

Supplementary Fig 74. ^{1}H NMR spectrum (500 MHz) of the collected DVS out-gas of **in-DBD** after complete degradation, captured in 5% v/v acetic acid solution. The only identified product protons are labeled; the poor integration quality is assumed to be a result of tautomerization of the protonated nitrogen(s), and/or inversion of the nitrogen center causing multiple chemical environments. The circle at 17 ppm is shown to highlight the lack of the caged proton in the degradation product, indicating complete degradation.

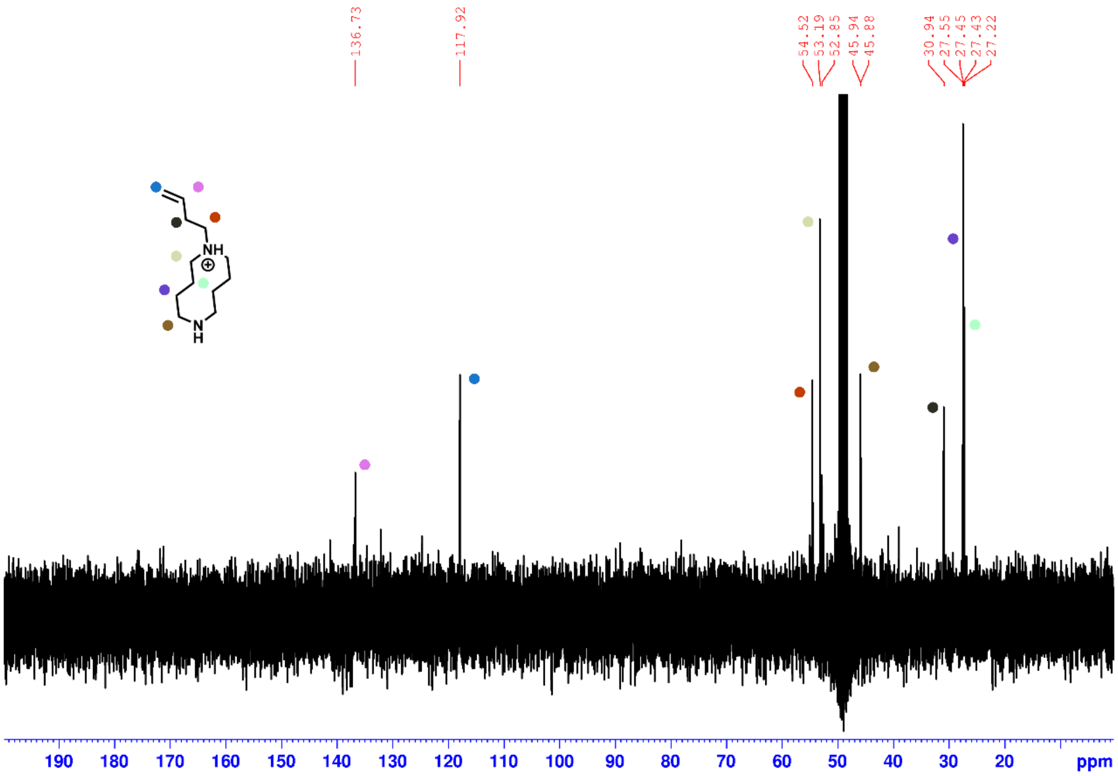

Supplementary Fig 75. ^{13}C NMR spectrum (101 MHz) of the collected DVS out-gas of **in-DBD** after complete degradation, captured in 5% v/v acetic acid solution. The only identified product carbons are labeled.

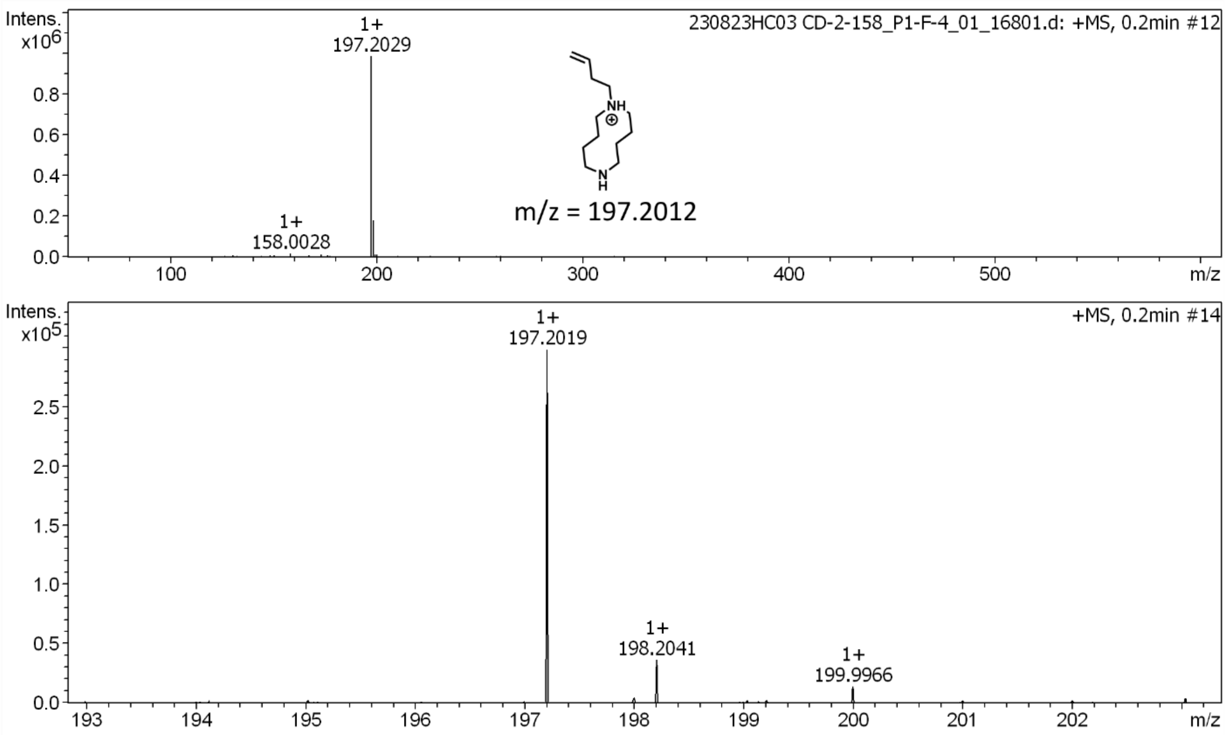

Supplementary Fig 76. Positive-ion mass spectrogram of the collected DVS out-gas of **in-DBD** after complete degradation, captured in 5% v/v acetic acid solution. The only identified product is shown, with its theoretical m/z.

DFT calculations:

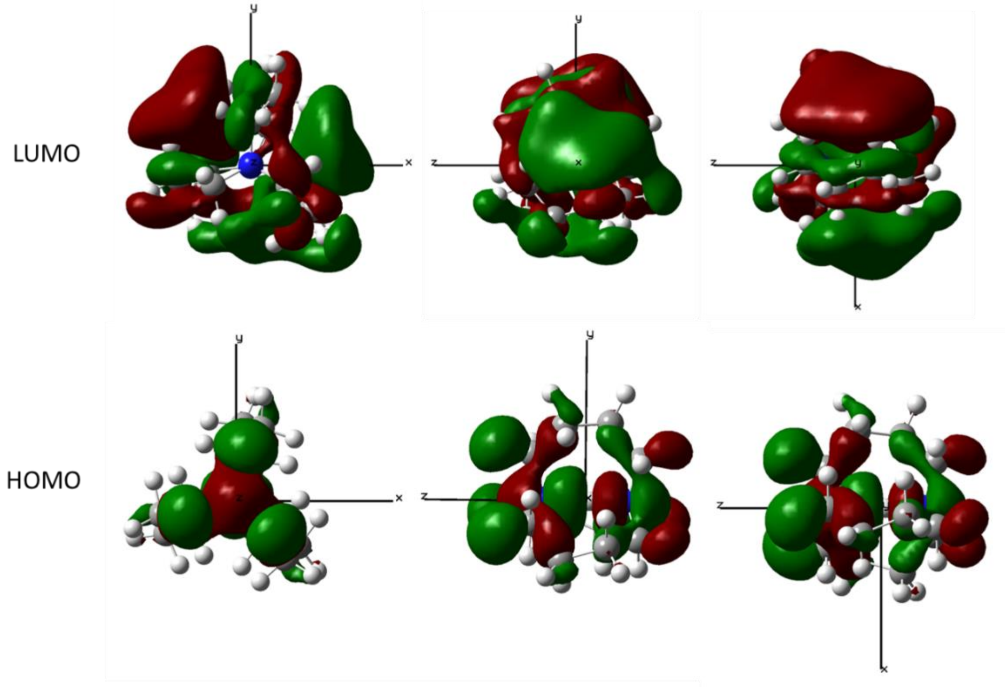

Supplementary Fig 77. Calculated orbital isosurfaces of the HOMO and LUMO of **in-DBD**. Calculations were performed at the ωB97XD/6-31g(d.p) level of theory.

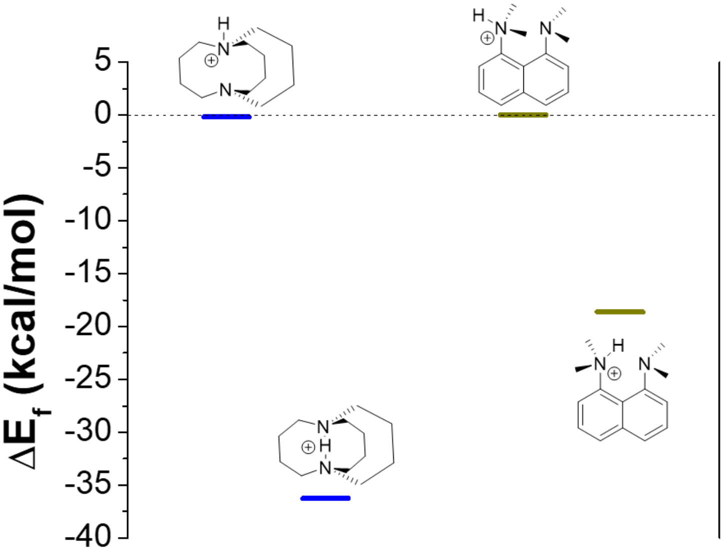

Supplementary Fig 78. Formation energy calculations of the relative energy of outside vs inside protonation for **in-DBD** (blue) and a typical proton sponge (green). Calculations were performed at the ωB97XD/6-31g(d.p) level of theory.

Supplementary Discussion

A blank NMR degradation test was performed with $NaBF_4$, to test the stability of the BF_4 anion to the NMR test conditions (2M KOH in CD_3OD at 80 °C). We observe a degradation of the BF_4 counterion similar to that reported for long hydrolysis[10] (Supplementary Fig 42). The fluctuations in the intensity of the **in-DBD** BF_4 form (Supplementary Fig 41) with no observed degradation products, are likely due to changes in the chemical environment from the loss of BF_4 over time.

A control experiment for DVS degradation studies was conducted showing that the hydroxide is the main form of degradation, where MMI iodide shows no degradation over the test conditions (Supplementary Fig 68, Supplementary Fig 69), whereas MMI hydroxide shows almost complete degradation (Supplementary Fig 59-Supplementary Fig 61).

Supplementary References

1. Alder, R. W., Casson, A. & Sessions, R. B. Inside- and outside-protonated ions from 1,6-diazabicyclo[4.4.4]tetradecane. *J. Am. Chem. Soc.* **101**, 3652–3653 (1978).
2. Robert, C. Cyclic Diacyl Diimides. II. 1,4-Phthalazinedione, 3,6-Pyridazinedione, and 4,5-Dihydro-3,6-pyridazinedione. *The Journal of Organic Chemistry* **24**, 1115–1118 (1962).
3. Stetter, H. & Spangenberger, H. Herstellung cyclischer Diamine des mittleren Ringgebietes durch Ringöffnung bicyclischer Verbindungen. *Chemische Berichte* **91**, 1982–1988 (1958).
4. Alder, R. W., Sessions, R. B., Bennet, A. J. & Moss, R. E. Reductive cleavage of propellane-type hydrazinium dications as a route to medium-sized ring bicyclic diamines with bridgehead nitrogen atoms. *J. Chem. Soc., Perkin Trans. 1* 603–609 (1982) doi:10.1039/P19820000603.
5. Chen, N. *et al.* Insight into the Alkaline Stability of N-Heterocyclic Ammonium Groups for Anion-Exchange Polyelectrolytes. *Angew. Chem. Int. Ed.* **60**, 19272–19280 (2021).
6. Fan, J. *et al.* Cationic Polyelectrolytes, Stable in 10 M KOHaq at 100 °C. *ACS Macro Lett.* **6**, 1089–1093 (2017).

7. Fan, J. *et al.* Poly(bis-arylimidazoliums) possessing high hydroxide ion exchange capacity and high alkaline stability. *Nat Commun* **10**, 2306 (2019).

8. Hugar, K. M., You, W. & Coates, G. W. Protocol for the Quantitative Assessment of Organic Cation Stability for Polymer Electrolytes. *ACS Energy Lett.* **4**, 1681–1686 (2019).

9. You, W. *et al.* Degradation of Organic Cations under Alkaline Conditions. *J. Org. Chem.* **86**, 254–263 (2021).

10. Freire, M. G., Neves, C. M. S. S., Marrucho, I. M., Coutinho, J. A. P. & Fernandes, A. M. Hydrolysis of Tetrafluoroborate and Hexafluorophosphate Counter Ions in Imidazolium-Based Ionic Liquids†. *J. Phys. Chem. A* **114**, 3744–3749 (2010).
